# Membrane Modulation of Super-Secreting “midi*Bacillus*” Expressing the Major *Staphylococcus aureus* Antigen – A Mass-Spectrometry-Based Absolute Quantification Approach

**DOI:** 10.3389/fbioe.2020.00143

**Published:** 2020-02-28

**Authors:** Minia Antelo-Varela, Rocío Aguilar Suárez, Jürgen Bartel, Margarita Bernal-Cabas, Tim Stobernack, Thomas Sura, Jan Maarten van Dijl, Sandra Maaß, Dörte Becher

**Affiliations:** ^1^Centre of Functional Genomics of Microbes, Department of Microbial Proteomics, Institute of Microbiology, University of Greifswald, Greifswald, Germany; ^2^Department of Medical Microbiology, University Medical Center Groningen, University of Groningen, Groningen, Netherlands

**Keywords:** shotgun-proteomics, SRM, biotechnology, membrane proteins, absolute protein quantification

## Abstract

*Bacillus subtilis* has been extensively used as a microbial cell factory for industrial enzymes due to its excellent capacities for protein secretion and large-scale fermentation. This bacterium is also an attractive host for biopharmaceutical production. However, the secretion potential of this organism is not fully utilized yet, mostly due to a limited understanding of critical rearrangements in the membrane proteome upon high-level protein secretion. Recently, it was shown that bottlenecks in heterologous protein secretion can be resolved by genome minimization. Here, we present for the first time absolute membrane protein concentrations of a genome-reduced *B. subtilis* strain (“midi*Bacillus*”) expressing the immunodominant *Staphylococcus aureus* antigen A (IsaA). We quantitatively characterize the membrane proteome adaptation of midi*Bacillus* during production stress on the level of molecules per cell for more than 400 membrane proteins, including determination of protein concentrations for ∼61% of the predicted transporters. We demonstrate that ∼30% of proteins with unknown functions display a significant increase in abundance, confirming the crucial role of membrane proteins in vital biological processes. In addition, our results show an increase of proteins dedicated to translational processes in response to IsaA induction. For the first time reported, we provide accumulation rates of a heterologous protein, demonstrating that midi*Bacillus* secretes 2.41 molecules of IsaA per minute. Despite the successful secretion of this protein, it was found that there is still some IsaA accumulation occurring in the cytosol and membrane fraction, leading to a severe secretion stress response, and a clear adjustment of the cell’s array of transporters. This quantitative dataset offers unprecedented insights into bioproduction stress responses in a synthetic microbial cell.

## Introduction

In 1974, Wacław Szybalski provided a contemporary interpretation of synthetic biology and prophesied that biology would eventually evolve from a descriptive discipline to a re(designing) one (Szybalski, 1974). The almost half century of research ensuing these assertions demonstrated their accuracy. The knowledge on the inventory of biological functions that microbial cells are able to integrate into their physiological and metabolic circuits has substantially increased, thereby paving the way for synthetic biology (Elowitz and Leibler, 2000; Gardner et al., 2000; Ro et al., 2006; Agapakis et al., 2010; Gibson et al., 2010; Hutchison et al., 2016; Fredens et al., 2019; Venetz et al., 2019). While four decades ago cell engineering mostly relied on random mutagenesis followed by screening processes (Parekh et al., 2000), the rapid development of high-throughput –omics approaches have altered this tendency. This leads to the development of systems metabolic engineering, a discipline that integrates metabolic engineering with systems and synthetic biology, with the ultimate goal of delivering highly sophisticated microbial cell factories (Lee et al., 2005, 2012; Chen et al., 2010; Makino et al., 2011). The proteome is an essential part of the endeavors to uncover the systematic properties of biological systems as proteins represent the central players in the complex metabolic and adaptational network (Aggarwal and Lee, 2003). While relative protein quantification is sufficient for a comparison of protein abundances between samples, these data do not meet the requirements for mathematical modeling in systems biology. Instead, in order to decipher physiological cell responses upon the onset of stress, absolute proteomic data are required. Accordingly, the past few years witnessed a general effort to produce suitable absolute protein data, mainly owed to the accomplishments of MS-based proteomics (Malmström et al., 2009; Maaß et al., 2011,Maaβ et al., 2014; Muntel et al., 2014; Wiśniewski and Rakus, 2014). However, some traits remain a challenge for this discipline. When it comes to absolute numbers of membrane proteins, few if any data are available, mainly due to the low abundance and high hydrophobicity of this subset of proteins. Nonetheless, knowledge on the absolute abundance of membrane proteins is essential, due to their involvement in essential biological processes. One of these essential mechanisms is protein secretion, a process that is nowadays heavily exploited in the biotechnology industry as it greatly facilitates the downstream processing of proteins. Importantly, secreted proteins can be produced in massive amounts to the extent that it leads to “bioproduction stress.” Thus, defining the changes in the membrane proteome that follow the overproduction of secreted proteins during bioproduction stress is of major relevance for the biotechnological sector (van Dijl and Hecker, 2013).

*Bacillus subtilis* has been extensively used as a microbial cell factory for industrial enzymes and biopharmaceuticals production (Schallmey et al., 2004; Westers et al., 2004; Zweers et al., 2008). This bacterium is also an attractive host for heterologous protein production due to its excellent fermentation and high product yield capacities (van Dijl and Hecker, 2013). Hence, a minimal strain of *B. subtilis* was engineered (Reuß et al., 2017) for heterologous production of “difficult proteins” natively secreted by *Staphylococcus aureus*, Aguilar and colleagues demonstrated that these proteins were successfully produced in the genome-reduced, but not in the wild-type strain (Suárez et al., 2019). Nonetheless, information on how the membrane proteome abundances was altered upon the production of this heterologous protein remained unknown.

Recently, we have published a study describing a method which simultaneously combines the comprehensiveness of shotgun-MS and the accuracy of targeted-MS, allowing the calculation of absolute membrane protein abundances in a living organism (Antelo-Varela et al., 2019). In the study presented here, we provide absolute membrane protein concentrations of the genome-reduced *B. subtilis* strain (midi*Bacillus*) IIG-Bs27-47-24 expressing IsaA (Suárez et al., 2019). To engineer this strain, 1401 genes were systematically deleted from the parental strain *B. subtilis* 168, which represents a genome reduction of 30.95% (Reuß et al., 2017). Notably, the midi*Bacillus* lacks the genes for eight major secreted *Bacillus* proteases, which were previously identified as one of the main bottlenecks for heterologous protein production (Pohl and Harwood, 2010; Krishnappa et al., 2013).

We quantitatively characterize the membrane proteome adaptation of midi*Bacillus* during production stress on the level of molecules per cell for more than 400 membrane proteins, which includes the determination of protein concentrations of ∼61% of the predicted transporters of this strain (Reuß et al., 2017). Furthermore, we determined protein allocation between main processes of the cell membrane during exponential growth, including protein translocation. This comprehensive dataset might be implemented in a mathematical model dedicated to fine-tune metabolic pathways, defining approaches to minimize stress and increase protein production.

## Materials and Methods

### Strain Construction

The bacterial strains and plasmids used in this study are listed in Supplementary Table S1. The s*paRK* genes were chromosomally integrated in the *amyE* locus of strain IIG-Bs27-47-24 by transformation of competent cells with plasmid pNZ8900. The “super-competence” of *Bacillus* was induced with mannitol as described by Rahmer et al. (2015). Subsequently, the strain IIG-Bs27-47-24 carrying the *spaRK* genes was transformed with the plasmid pRAG3:IsaA for the inducible expression of IsaA, as previously described (Suárez et al., 2019). Moreover, secretion of IsaA was directed into the growth medium by N-terminal fusion to the signal peptide of the xylanase XynA (SP_XynA_) of *B. subtilis* (Gilbert et al., 2017).

### Subtilin Production

For subtilin production, *B. subtilis* ATCC 6633 was grown overnight in LB medium and samples were harvested by centrifugation (10,000 × *g* for 10 min at 4°C). The free-cell supernatant containing subtilin was collected and subsequently incubated at 80°C for 10 min, prior to freezing at −20°C.

### Growth Conditions and Protein Preparation

For all proteomics analysis, bacteria were grown in LB medium. Exponentially growing cells (optical density at 600 nm [OD_600_] of 0.9) were induced with 1% subtilin (v/v), and samples were taken 120 min after the onset of induction. Bacteria grown without subtilin were collected at the same time point and used as control. For every experiment, three independent biological replicates were analyzed. 30 mL of bacterial cell culture was harvested by centrifugation (10,000 × *g* for 15 min at 4°C), and cell pellets were washed three times with TE buffer (20 mM Tris, 10 mM EDTA, pH 7.5). The corresponding supernatants were filtered and stored at −80°C for further preparation of the extracellular protein fraction. Cells were mechanically disrupted using the FastPrep24 instrument (MPBiomedicals). Cell debris was removed by centrifugation (20,000 × *g* for 10 min at 4°C), and the recovered supernatant was designated as whole cell extract. Protein concentration of these extracts was determined by a Bradford-based assay (Bradford, 1976).

### Membrane Enrichment

An aliquot of the whole cell extract with a protein content of 5 mg was used as starting material for membrane preparation. This lysate was filled up to 1.5 mL TE buffer and subjected to ultracentrifugation (100,000 × *g* at 4°C). The supernatant was designated as cytosolic fraction and the pellet was detached from the bottom by adding 0.75 mL high salt buffer (10 mM EDTA, 1 M NaCl, 20 mM Tris-HCl, pH 7.5) and incubation in an ultrasonic bath for 5 min at room temperature. This was followed by pipetting the suspension up and down until the pellet was homogenized. The pipette was then rinsed with 0.75 mL high salt buffer and the solution was incubated in a rotator at 8000 r/min and 4°C for 30 min, followed by ultracentrifugation under the same conditions as above. Pellet resuspension and ultracentrifugation were then performed with alkaline carbonate solution (10 mM EDTA, 100 mM Na_2_CO_3_, 100 mM NaCl, pH 11), and in a final step with TEAB (50 mM). The pellet containing the final crude membrane extract was resuspended in 70 μL 6 M urea/2 M thiourea.

### Sample Preparation for MS Analysis

For shotgun-based absolute quantification, 10 μg of crude membrane and cytosolic extract were used for protein digestion using the S-Trap protocol according to the manufacturer (ProtiFi). The cytosolic fraction was also used for quantification of the IsaA protein in this particular sub proteome. UPS2 proteins (Sigma–Aldrich–Merck) were added in a 1:4 ratio (2.5 μg). For LC/MS analysis, 4 μg of peptide mixture per biological replicate was desalted using C18–Zip Tips (Merck Millipore). Peptide concentration was determined using the Pierce Quantitative Colorimetric Peptide Assay (Thermo Fisher Scientific). Preparation of whole cell and membrane extracts for targeted-MS followed the same digestion protocol as described above, except for the addition of UPS2 standards. Instead, samples were spiked with heavy peptides of the anchor proteins used in this study–QcrA and YwbN–to a final amount of 5 pmol. A detailed list of used peptides and their optimized transitions is available in Supplementary Table S2.

### Preparation of Extracellular Protein Fraction for IsaA Quantification

Extracellular samples have been prepared according to Bonn et al. (2014). Briefly, the volume of culture supernatant correspondent to 10 μg, plus 2.5 μg of UPS2 standards were incubated with 12.5 μL of primed StrataClean beads (Agilent). Beads were subsequently precipitated by centrifugation, washed, resuspended in 1 mL TE buffer, dried, and resolved in 20 μL reducing SDS sample buffer [125 mM Tris-HCl pH 6.8, 20% (v/v) glycerol, 4% (w/v) SDS, 3.75% (v/v) β-mercaptoethanol, 0.04% (w/v) bromophenol blue]. Samples were incubated at 98°C for 10 min and subsequently the respective proteins were separated by SDS-PAGE. The electrophoretic run was performed at 150 V for approximately 15 min to ensure that the proteins were released from the strata beads. After fixation and staining, one piece of the gel corresponding to the extracellular protein extract was excised and tryptically digested as previously described (Bonn et al., 2014). Peptide concentration was determined using the Pierce Quantitative Colorimetric Peptide Assay (Thermo Fisher Scientific). For LC/MS analysis, 1 μg of peptide mixture per biological replicate was desalted using C18–Zip Tips (Merck Millipore).

### Determination of Cell Size

Bacterial cell size was determined at the harvesting point for both conditions–control and induction [1% subtilin (v/v)]. For this purpose, midi*Bacillus* cells were photographed with a size scale using a light microscope (Leica DM2500 LED) coupled to a digital camera (Leica DMC2900). Length and width of the cells were determined with the help of ImageJ software (Abràmofff et al., 2005) as described elsewhere (Maaß et al., 2011). In summary, 180 midi*Bacillus* cells were imaged for each condition allowing for calculation of cell size distribution and standard deviation. Surface area of cells was calculated assuming a cylinder and two hemispheres for the rod-shaped *Bacillus* cells. A table showing the average sizes of all measured midi*Bacillus* cells per condition is available in Supplementary Table S3.

### SDS-PAGE and Western Blotting for IsaA Visualization

Protein samples were separated by SDS-PAGE as described previously (Eymann et al., 2004). Before loading, whole cell and crude membrane extract samples were corrected for protein concentration in order to ensure equal sample loading for a quantitative signal interpretation. Proteins separated by SDS-PAGE were blotted onto a nitrocellulose membrane (GE Healthcare). Subsequent immunodetection of bound proteins was performed with the human monoclonal antibody 1D9 against IsaA (van den Berg et al., 2015). For visualization of antibody binding, the 1D9 antibodies were labeled with IRDye 800CW (LiCor Biosciences). Fluorescence was recorded at 800 nm with an Odyssey Infrared Imaging System (LiCor Biosciences). Three independent biological replicates were analyzed for each tested condition.

### Shotgun MS Analysis

Digested protein mixtures of cytosol (2 μg), membrane (2 μg), and extracellular (1 μg) fraction were separated on an Easy nLC 1200 coupled online to an Orbitrap Elite mass spectrometer (Thermo Fisher Scientific). In-house self-packed columns [i.d. 100 μm, o.d. 360 μm, length 200 mm; packed with 3.0 μm Dr. Maisch Reprosil C18 reversed-phase material (ReproSil-Pur 120 C18-AQ)] were first loaded with 12 μL of buffer A [0.1% (v/v) acetic acid] at a maximum pressure of 400 bar and subsequently eluted. Elution of peptides took place with a non-linear 166 min gradient from 1 to 99% buffer B [0.1% (v/v) acetic acid in 95% (v/v) acetonitrile] at a constant flow rate of 300 nL/min. Spectra for the eluting peptides were recorded in the Orbitrap at a resolution of *R* = 60,000 with lockmass correction activated. After acquisition of the Full-MS spectra, up to 20 dependent scans (MS/MS) according to precursor intensity were performed in the linear ion trap after collision-induced dissociation (CID) fragmentation.

### Targeted MS Analysis

Purified synthetic peptides with heavy arginine and lysine were obtained from Thermo Fisher Scientific. For each protein, four peptides were designed and three were used for quantification (Supplementary Table S4). For SRM-method development, all feasible transitions of QcrA and YwbN were monitored to keep four to six transitions per peptide with the highest peak areas. After selection, collision energies were optimized for each of these transitions and the final transition list was generated (Supplementary Table S2). In order to check for the dynamic range of the AQUA peptides, known amounts of QcrA and YwbN (0.001–10 pmol on column) were used to generate a calibration curve. Linear regression and *r*^2^ were calculated. Based on the calibration curve, 5 pmol of each heavy labeled peptide was added to three biological replicates of the digested whole cell and membrane extract samples for each tested condition–control and 1% subtilin induction–enabling the calculation of absolute amounts of target peptides (Maaß et al., 2011). Digested protein mixtures (2 μg) were separated on an Easy nLC 1000 (Thermo Fisher Scientific) coupled to a triple quadrupole mass spectrometer (TSQ Vantage, Thermo Scientific) operated in nano-electrospray mode. Peptide separation was carried out using in-house self-packed columns [i.d. 100 μm, o.d. 360 μm, length 200 mm; packed with 3.0 μm Dr. Maisch Reprosil C18 reversed-phase material (ReproSil-Pur 120 C18-AQ)] by applying a non-linear 81 min gradient from 1 to 99% buffer B (0.1% v/v acetic acid in acetonitrile) at a constant flow rate of 300 nL/min. For ionization, 2400 V spray voltage and 240°C capillary temperature were used. The selectivity for both Q1 and Q3 was set to 0.7 Da (FWHM). The collision gas pressure of Q2 was set at 1.2 mTorr. TSQ Vantage was operated in SRM mode.

### LC/MS Data Analysis of Shotgun MS and Global Absolute Quantification of Membrane Proteins

For shotgun proteomics, three different proteome fractions were analyzed: cytosol, membrane, and extracellular. Nevertheless, cytosolic and extracellular fractions were only used to quantify absolute abundances of IsaA, whereas for the membrane fraction, the entire dataset was analyzed. Thus, as the employed method for absolute protein quantification was exclusively designed for the analysis of the hydrophobic fraction (Antelo-Varela et al., 2019), the absolute abundances presented here focus on the membrane proteins of *B. subtilis*. For data processing and protein identification, raw data were imported into MaxQuant (1.6.3.3) (Tyanova et al., 2016a) analyzed with an Andromeda search engine (Cox et al., 2011), and processed via the iBAQ algorithm (Schwanhäusser et al., 2011). Database search was carried out against a reversed *B. subtilis* IIG-Bs27-47-24 database (Reuß et al., 2017) with manually added UPS2 protein sequences, IsaA, SpaR, and SpaK sequences and with common contaminants added by MaxQuant. The database search was performed with the following parameters: peptide tolerance: 4.5 ppm; min fragment ions matches per peptide: 1; match between runs was enabled with default settings; primary digest reagent: trypsin; missed cleavages: 2; fixed modification: carbamidomethyl C (+ 57.0215); and variable modifications: oxidation M (+ 15.9949), acetylation N, and K (+ 42.0106). Results were filtered for 1% FDR on spectrum, peptide, and protein levels. All identification and quantitation data are summarized in Supplementary Table S5. The MS proteomics data have been deposited to the ProteomeXchange Consortium via the PRIDE (Deutsch et al., 2017; Perez-Riverol et al., 2019) partner repository with the dataset identifier PXD015496. Proteins were only considered for further analysis if quantified in three out of three biological replicates.

A permutation-based FDR approach implemented in the Perseus platform was used to calculate significantly changed membrane protein abundances during overproduction (Tyanova et al., 2016b). The following parameters were used: number of randomizations: 250; FDR: 0.05; S0: 0.1.

### LC/MS Data Analysis of Targeted MS and Absolute Quantification of Reference Peptides

For targeted MS analysis, we analyzed three independent biological replicates per tested condition for two different fractions: whole cell and membrane extract. Raw files were processed using Skyline 4.2 (MacCoss Lab Software; MacLean et al., 2010). A peptide ratio of native and heavy species was based on four to six transitions that were averaged. Based on the added amount of heavy peptides, the absolute quantity of native proteins could be calculated (Maaß et al., 2011).

## Results

### Calculation of SRM-Derived Enrichment and Correction Factors

**FIGURE 1:**
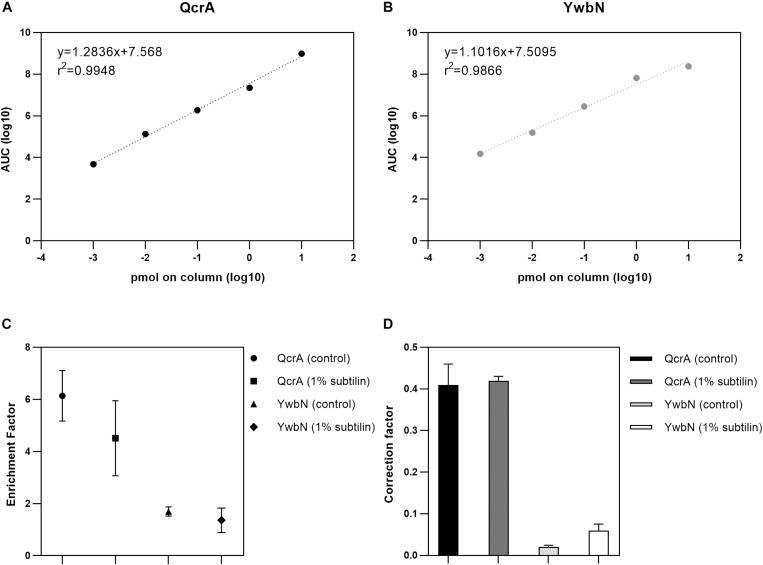
Results of absolute quantification by targeted MS. **(A)** Linear regression of the five-point calibration of QcrA. Slope, intercept, and *r*^2^ are depicted. *X*-axis shows the log_10_-transformed peptide concentration of the corresponding dilution and *y*-axis shows the log_10_-transformed area under the curve (AUC) derived from SRM. **(B)** Linear regression of the five-point calibration of YwbN. Slope, intercept, and *r*^2^ are depicted. *X*-axis shows the log_10_-transformed peptide concentration of the corresponding dilution and *y*-axis shows the log_10_-transformed area under the curve (AUC) derived from SRM. **(C)** Digested whole cell and membrane extract were measured and QcrA and YwbN amounts calculated. Geometric forms depict enrichment factor for both anchor proteins and for both physiological conditions, with the respective error bars for three replicates. **(D)** Absolute molar amounts of shotgun and targeted approaches were used to calculate a correction factor derived from both these approaches. Bar plot depicts correction factor for both anchor proteins and for both tested physiological conditions.

A crucial point to determine absolute membrane protein abundances relies on accurate calculation of an enrichment factor, as the membrane-enriched protein fraction does not reflect the cell’s membrane proteome in its native state. Furthermore, it is indispensable to apply a correction factor to the shotgun-MS derived data, as the UPS2 proteins do not necessarily replicate the unique physicochemical properties of membrane proteins (Antelo-Varela et al., 2019). Thus, in this study, we used two anchor proteins–QcrA and YwbN–serving two different functions. The Rieske-iron subunit membrane protein QcrA was used to calculate both the enrichment and correction factors for absolute quantification of membrane proteins, whereas the secreted Dyp-type peroxidase YwbN served as control, demonstrating that the applied method provides reliable absolute abundance values exclusively for membrane proteins (Jongbloed et al., 2004; Goosens et al., 2013). In order to check for the dynamic range of QcrA and YwbN, a calibration curve spanning five orders of magnitude was generated and a linear regression was calculated. Our results show that we are able to accurately quantify these proteins across the whole dynamic range with an *r*^2^ of 0.9948 and 0.9866 for QcrA and YwbN, respectively (Figures 1A,B, respectively).

To determine the enrichment factor between whole cell extract and enriched membrane protein sample, a ratio between the absolute molar amounts of the membrane protein QcrA in the membrane and the whole cell extracts was calculated resulting in values of 6.14 and 4.51 for control and 1% subtilin induction conditions, respectively (Figure 1C). Even though there is slightly lower enrichment for the induced fraction, the difference is not significant (*p* = 0.63, paired *t*-test), and might simply be a reflection of biological variance. The same calculations were performed for YwbN by determining the ratio between membrane and whole cell extract molar amounts, resulting in values of 1.70 and 1.36 for control and 1% subtilin induction conditions, respectively (Figure 1C). This fitted our expectations, as the enrichment factor is considerably lower for not membrane-associated, YwbN.

In addition to the enrichment factor, a correction factor was determined by calculating the ratio between the absolute molar amounts of QcrA obtained by SRM and shotgun-MS. We calculated a median ratio of 0.41 and 0.42 for control and induction conditions, respectively (Figure 1D). This shows that UPS2-based absolute quantification provides a slight overestimation of total protein abundances, which is in accordance with the recently published study on global membrane protein quantification (Antelo-Varela et al., 2019). This overestimation may be due to different physicochemical properties between this human set of proteins and the bacterial membrane proteome. However, this overestimation is not affected by different physiological conditions as the correction factor is similar for both control and production stress conditions. Just like for QcrA, we also determined the correction factor for YwbN by calculating a ratio between absolute molar amounts of SRM and shotgun-derived data. This provided a median ratio of 0.02 and 0.06, for control and induced conditions, respectively (Figure 1D). This comprises an overestimation of one order of magnitude, again demonstrating that the employed method for absolute protein quantification is designed to meet the requirements of hydrophobic proteins, but not their soluble counterparts.

Both the correction and enrichment factor of QcrA were used to calibrate the data derived from the shotgun approach as previously described (Antelo-Varela et al., 2019).

### Absolute Quantification of Membrane Proteins

**FIGURE 2:**
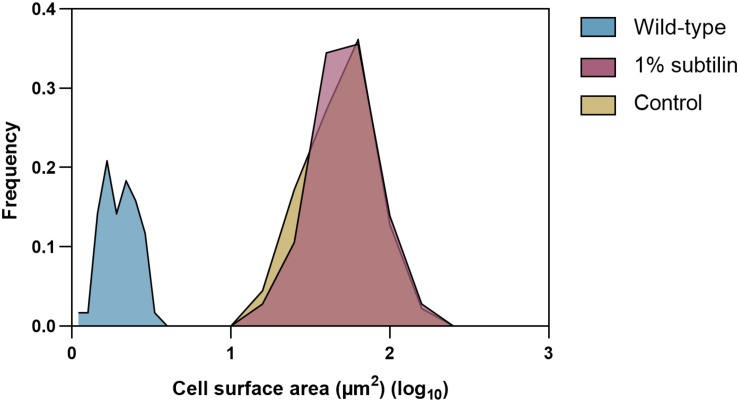
Cell size distribution of the 180 measured *midiBacillus* cells for each tested condition and 120 measured *B. subtilis* parental strain. The *y*-axis shows how frequently a given size appears in the dataset (μm^2^) and the *x*-axis represents the log value of the calculated surface area (μm^2^) of *midiBacillus* and parental strain *B. subtilis*. Blue curve represents the distribution of the parental strain grown in minimal media (Antelo-Varela et al., 2019), and yellow and red show the size distribution of *midiBacillus* during control and induced conditions, respectively.

From the biotechnological perspective, knowledge of protein cell density on cell surface is of the foremost importance, as it is crucial to optimize and enhance microbial cell factories. Hence, in this study, we provide absolute membrane protein concentrations per surface area. Determination of protein copy numbers per cell surface area was possible after preparation of a cell count calibrated sample. In order to do so, the same disruption method as developed by Maaß et al. (2011) was employed, since it has proven to provide disruption efficiencies better than 99% for *B. subtilis*. Moreover, the average size of midi*Bacillus* cells for the two tested physiological conditions was determined using a light microscope. We observed that cells of this strain tend to form long filaments of non-separated cells, which seems to be in accordance with previous observations (Reuß et al., 2017). A table showing the average sizes of all measured midi*Bacillus* cells per condition is available in Supplementary Table S3. Our measurements showed that cell sizes follow a normal distribution thus, allowing to confidently calculate the cell surface area (Figure 2).

Absolute protein abundances of midi*Bacillus* were calculated by linear regression of the log-transformed iBAQ intensities against known log-transformed absolute molar amounts of the spiked-in UPS2 standards (Schwanhäusser et al., 2011) (Supplementary Figure S1). The absolute protein molar amounts of shotgun-MS derived quantification were subsequently calibrated by applying both the correction and enrichment factor derived from the SRM approach as described above. In order to provide reliable data, only proteins quantified in three out of three biological replicates were considered for further analysis. In this study, we were able to quantify 448 membrane proteins, of which 22 proteins are exclusively quantified in one of the physiological conditions (Table 1 and Supplementary Table S5). Absolute protein amounts per microgram of crude membrane extract, protein concentrations, copy numbers per cell surface area, and molecules per cell for all membrane proteins quantified are available in Supplementary Table S5. From the 448 membrane proteins, 187 contain less than three predicted TMDs and 239 have three or more predicted TMD, according to HMMTOP2.0 (Tusnády and Simon, 2001). From this quantified fraction, we verified that 150 proteins significantly changed their abundance in response to IsaA induction (Supplementary Table S6).

**TABLE 1:** Determined protein amounts (in molecules per surface area) of ON/OFF proteins in control and induction conditions.

			Molecules/μm^2^	
			
Protein	Function	MW [kDa]	Control	1% Subtilin
YfhA	Acquisition of iron	35.955	0.0010	
YufO	Uptake of guanosine	56.299	0.0005	
YufQ	Uptake of guanosine	33.738	0.0003	
YvsH	Uptake of lysine	50.258	0.0015	
*Nar*I	Nitrate respiration	25.296	0.0049	
NarH	Nitrate reductase (γ subunit)	55.472	0.0010	
NarG	Nitrate reductase (α subunit)	139.1	0.0022	
NarK	Nitrite extrusion	42.925	0.0110	
YxzE	Unknown	6.8355	0.0127	
HutM	Histidine uptake	51.624	0.0032	
YbfB	Unknown	45.235	0.0003	
KapB	Control of sporulation initiation	14.668		0.0007
YlmA	Unknown	29.699		0.0007
YwoG	Unknown	43.08		0.0008
YcgR	Unknown	32.49		0.0009
Des	Phospholipid desaturase	40.708		0.0013
YkpB	Unknown	33.572		0.0019
YhaJ	Unknown	19.64		0.0020
YfmJ	Unknown	36.662		0.0038
YwqA	Unknown	106.03		0.0041
Pss	Biosynthesis of phospholipids	19.613		0.0159
LiaH	Resistance against oxidative stress	25.698		0.0167

*Absolute amounts for all quantified proteins can be found in Supplementary Table S5.*

During exponential growth, concentrations of proteins span from 0.0004 molecules/μm^2^ for the two-component sensor kinase KinA to about 2 molecules/μm^2^ for the ATP synthase AtpF. Due to unexpected results, we had a closer inspection of proteins presenting very low copy numbers and found that the vast majority are involved in the transport of very specific substrates (OpuCA, OpuCC), have unknown function (YcbM, YlmA), or are part of two-component sensor kinase complexes, usually related to sporulation (KapB, KinA, KinE) (Supplementary Table S5).

This study has also enabled the calculation of stoichiometries for the highly conserved Sec translocation system responsible for the secretion of IsaA (Supplementary Table S7). This showed that accessory components SecDF and SpoIIIJ (MisCA) are present in about the same amounts as the main translocation channel component SecY in midi*Bacillus*, which is in agreement with the previously published data for *Escherichia coli* (Papanastasiou et al., 2012). On the other hand, the SecE channel component was not detected and SecG was detected in fourfold lower amounts than SecY. The latter could be due to the fact that SecG of Gram-positive bacteria may be poorly retained in the channel and released into the medium (García-Pérez et al., 2018). Even though the results from this study are not entirely in accordance with the ratios reported for *E. coli*, it should be mentioned that they agree with the ratios reported by recently published data exclusively dedicated to the determination of absolute membrane protein abundances in *B. subtilis* (Antelo-Varela et al., 2019).

### Heterologous Production of IsaA

**FIGURE 3:**
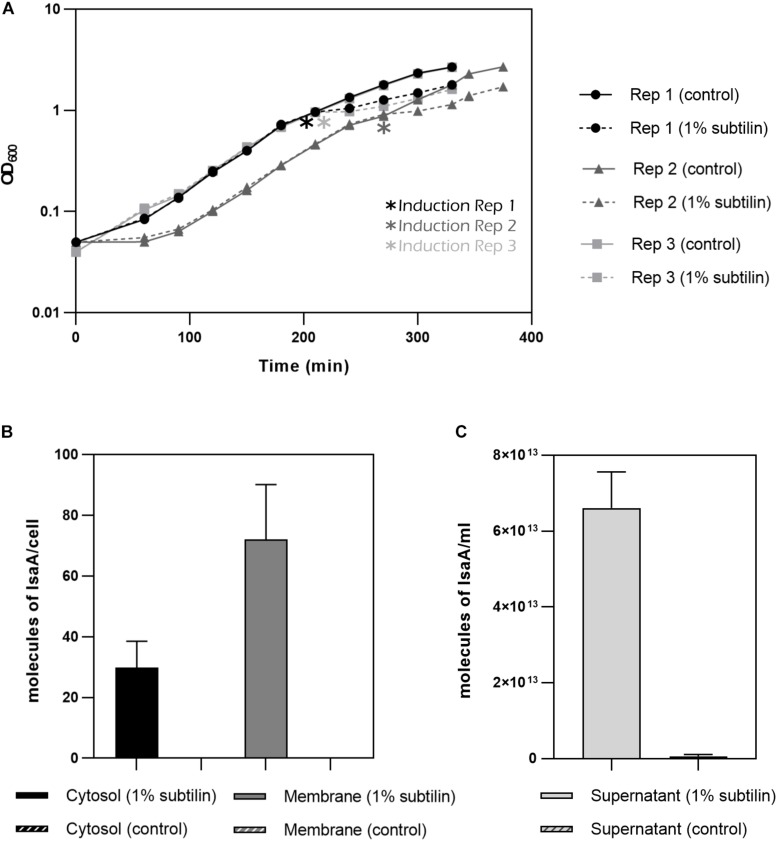
IsaA production by *midiBacillus*. **(A)** Growth curves for the three biological replicates until harvesting point. Asterisks indicate moment of induction. Filled lines represent the strains grown under control condition and discontinued lines represent the strains in which 1% subtilin was added. Geometric shapes represent different biological replicates (circle–*B. subtilis* IIG-Bs27-47-24 replicate 1; triangle–*B. subtilis* IIG-Bs27-47-24 replicate 2; square–*B. subtilis* IIG-Bs27-47-24 replicate 3). **(B)** Bar plot depicting number of molecules/cell of IsaA for cytosolic and membrane fraction (filled bar represents induced condition and striped plot represents control condition). No IsaA was quantified during control conditions. Standard deviation between biological replicates is depicted per each condition. **(C)** Bar plot depicting molecules of IsaA/mL for extracellular fraction (filled bar represents induced condition whereas striped bar represents control condition). Almost no IsaA was found for control conditions. Standard deviation between biological replicates is depicted per each condition.

The aim of this study is to understand the changes happening in the membrane proteome in response to the secretion of a heterologous protein. Thus, to induce production of IsaA, 1% subtilin was added to exponentially growing cells and the effect of induction was assessed 2 h after the onset of stress (Figure 3A). We have determined absolute abundances of IsaA in three different fractions of the cell: cytosol, membrane and extracellular, by converting iBAQ intensities in relation to those of the UPS2 standards. Even though the method applied in this study is tailored for quantification of hydrophobic proteins, the UPS2 strategy has already proven to be accurate for cytosolic proteins (Schwanhäusser et al., 2011). For accurate determination of IsaA in the extracellular fraction, UPS2 were added prior to StrataClean bead binding in order to ensure correction for putative sample loss during the experimental workflow.

For the cytosolic fraction, we verified that midi*Bacillus* accumulated around 30 molecules of IsaA per cell during overproduction conditions. This amount increases for more than double in the membrane fraction with 72 molecules of IsaA per cell (Figure 3B). Finally, for the extracellular fraction, we quantified 6.61E^+13^ molecules/mL (∼3 mg IsaA/mL or ∼12 molecules of IsaA/cell) for the induced and 6.33E^+11^ molecules/mL (0.3 mg IsaA/mL or 0.21 molecules of IsaA/cell) in the control samples (Figure 3C). Additionally, we calculated the secretion rate for IsaA, and determined that the Sec machinery secretes, on average, 2.41 molecules of IsaA per minute upon induction. Under control conditions, the Sec translocon secretes only 0.05 molecules of IsaA per minute, demonstrating the successful induction of IsaA translocation in midi*Bacillus*.

Comparing the results of secreted IsaA to other examples of protein products that are successfully produced in *B. subtilis*, we verified that midi*Bacillus* is capable of secreting well within the margins of biotechnological products (Westers et al., 2004). In this experiment, we produced 3 mg/L of IsaA to the cultivation medium, which is three times more than what is industrially produced for IFN-alpha 2, which is in the range of 0.5–1 mg/L (Palva et al., 1983).

### General Membrane Proteome Adaptation to Heterologous Protein Production

The membrane proteome is crucial for cellular homeostasis and life in general. This is shown by the wide range of vital processes played by membrane proteins, such as energy transduction, phospholipid biosynthesis, cell wall biogenesis, cell division, and protein translocation (Zweers et al., 2008). Naturally, the overproduction of heterologous proteins will disrupt the membrane proteome balance, as these proteins will have to endure abnormal levels of secretion.

Our data show a significant increase in the total number of membrane protein molecules/cell during induced secretion of IsaA in comparison to control conditions (*p* = 0.0004, paired *t*-test). To validate if the number of molecules/cell dedicated to translational functions increased during induction, we analyzed the cytosolic fraction and found that∼4% more molecules are dedicated to this particular cell function. We were able to accurately quantify ∼50% of proteins predicted to exhibit translational functions and, of these, ∼23% significantly increase their abundance (data not shown). This supports the observation that the translational machinery is boosted upon IsaA induction, resulting in an increase in the total number of molecules/cell. Hence, in order to be able to compare the changes happening within the membrane fraction between production and control conditions, the number of molecules/surface area (μm^2^) was normalized by the total amount of molecules/μm^2^ of the corresponding replicate.

**FIGURE 4:**
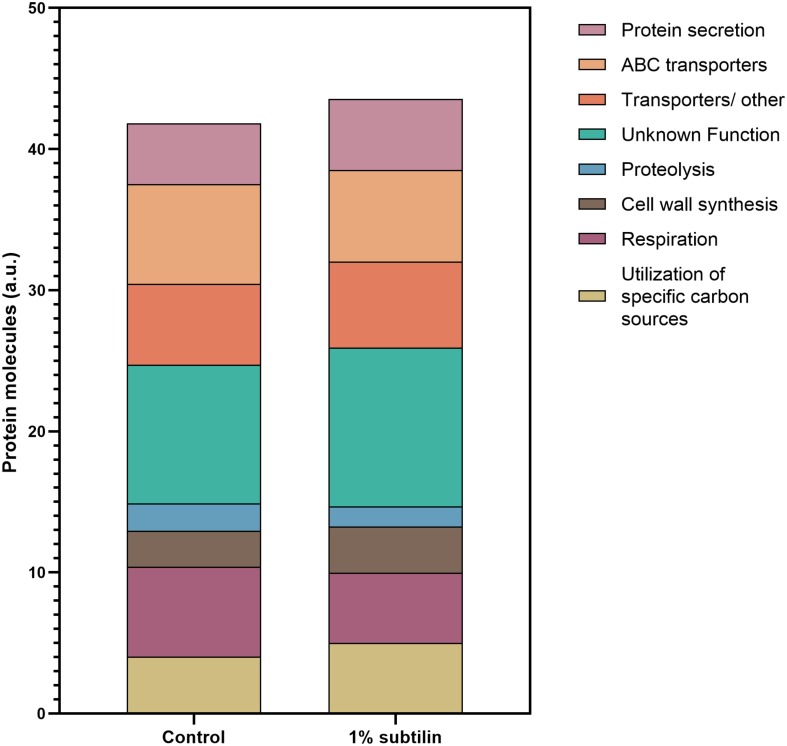
Assignment of membrane protein copy number per cell surface to a specific cellular function. Data were assigned to a specific function according to SubtiWiki (Mäder et al., 2012) gene categorization. Data are clustered in one single hierarchical level. Depicted on the graph are the eight most relevant functions for the interpretation of this study. Different functions are depicted in different colors. *Y*-axis represents the number of protein molecules (artificial units) dedicated to a specific cell functions, whereas the *x*-axis shows the two tested conditions, control and 1% subtilin induction.

**FIGURE 5:**
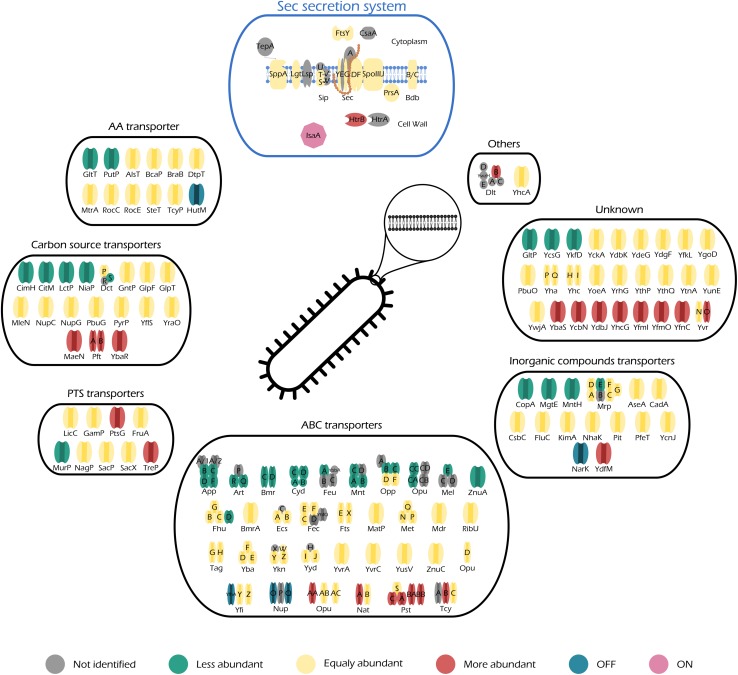
Illustration of all quantified transporters of *midiBacillus* and their respective changes during production stress. Transporters are separated in different categories according to SubtiWiki (Mäder et al., 2012) gene categorization. This figure also includes a schematic representation of the principal secretion system of *Bacillus subtilis*–Sec. Color code is depicted below the figure and it illustrates the changes of the transporter relatively to control conditions.

Our results show that the most abundant group of membrane proteins does not have a characterized function, with ∼10% of the quantified protein molecules being assigned to this functional category, with a clear increase in the induced physiological state (Figure 4 and Supplementary Figures S2, S3). This is presented with more detail in Table 1, where most ON proteins–only expressed after induction–have an unknown function. Additionally, we found that ∼ 30% of the proteins with an unknown function show a significant increase in abundance (Figure 5–unknown), which comprises an overall increase of ∼1% in the number of molecules/μm^2^ (Figure 4).

Our results show a general increase of ∼1% in the number of molecules/μm^2^ involved in cell wall synthesis (Figure 4 and Supplementary Figures S2, S3). Particularly, our data show a specific increase in the abundance of PBPs (Supplementary Table S6). For instance, we found that PonA, a class A penicillin-binding protein 1A/1B, contributing to cell elongation and division, significantly increases in abundance during secretion stress (2.2-fold change, 0.1 molecules/μm^2^ during induction conditions) (Supplementary Table S6). This underlines the essential role of PonA in maintaining cell wall homeostasis and PG integrity during environmental insults, such as abnormal secretion rates. Moreover, our data show that PbpD, also belonging to the PBPs class A functional family, acting as glucosyltransferase/transpeptidase, significantly increases during IsaA induction (2.01-fold change, 0.1 molecules/μm^2^ during induction conditions) (Supplementary Table S6). A previously published study showed that the molecular chaperone PrsA is required for the stability of several PBPs, including PBP4, as the proteome analysis suggested that this protein was one of the main PrsA-dependent proteins in the membrane (Hyyryläinen et al., 2010). Therefore, it might be possible that the increase in abundance of PbpD is due to the increased chaperone activity of PrsA, and/or it might be a response to exacerbated protein secretion, leading to a natural need of the cell to maintain PG homeostasis. Besides the general response of the proteins involved in PG homeostasis, there is also a significant increase in abundance of proteins responsible for LTA synthesis (Supplementary Table S6). LTA is a polymer linked to the membrane by a lipid anchor and it was shown to play a crucial role in bacterial growth and physiology, cation homeostasis, and cell division (Percy and Gründling, 2014). Our results show a significant increase in three of the four paralogs of the *S. aureus* LTA synthase described in *B. subtilis*–LtaS (2.52-fold change, 0.04 molecules/μm^2^), YfnI (4.81-fold change, 0.06 molecules/μm^2^), and YvgJ (4.17-fold change, 0.004 molecules/μm^2^) (Supplementary Table S6). Besides the amount of crosslinking of the thick PG layer of the cell wall, which determines the size of the holes in the PG network, charge density may also play a significant role in the efficiency of secretion. The charge of the cell wall is mediated by the degree of D-alanyl esterification of wall teichoic acid (WTA) and LTA, encoded by the *dlt* operon (Neuhaus and Baddiley, 2003). Our data show a significant increase in abundance of DltB (2.48-fold change, 0.03 molecules/μm^2^ during induction), a protein described to be under the regulation of cell surface stress sigma factor σ^M^ (Eiamphungporn and Helmann, 2008) (Supplementary Table S6). The same result has also been reported by Hyyryläinen et al. (2005), when investigating the response of *B. subtilis* to secretion stress. This group has also reported a decreased level of proteolysis during production. This result is in accordance with the observations of our study, as we verify a decrease of ∼0.6% in the number of molecules/μm^2^ dedicated to this cell function. It is expected that this decrease will most likely affect the rate of turnover of the cell wall (Hyyryläinen et al., 2005).

Another trend evidenced by the results of this study is the increase of ∼0.8% in the number of protein molecules/μm^2^ dedicated to the utilization of specific carbon sources upon IsaA induction. This might be a result of the “metabolic burden” resultant of heterologous protein production (Figure 4 and Supplementary Figures S2, S3). Naturally, the introduction of foreign DNA in the host organism leads to changes in the core metabolism, as a certain amount of cellular energy is required to maintain the newly introduced DNA (Glick, 1995). Moreover, the higher the rate of production, the greater is the amount of energy required to maintain this production within the host cell. This might also result in a rearrangement of the metabolic needs, hence forcing the host organism to seek for alternative sources of carbon and suppress the ones that are detrimental during secretion.

The results of this study also show that there is a significant decrease in the number of molecules dedicated to cell respiration during induction conditions (Figure 4 and Supplementary Figures S2, S3). We observe that proteins constituting the aerobic respiratory chain of *B. subtilis* significantly decrease their abundance, while proteins belonging to the nitrate reductase supercomplex are absent in secretion stress condition. For instance, during induction, the cell contains 0.07 molecules/μm^2^ of QcrA (−2.97-fold change, 0.07 molecules/μm^2^ upon induction), which is in the same range as the cytochrome-c oxidase cluster–CtaC (−4.07-fold change, 0.05 molecules/μm^2^ upon induction), CtaD (−3.52-fold change, 0.03 molecules/μm^2^ upon induction), CtaE (−3.07-fold change, 0.05 molecules/μm^2^ upon induction), and CtaF (−3.24-fold change, 0.02 molecules/μm^2^ upon induction) (Table 2).

**TABLE 2:** Regulation and function of quantified proteins involved in respiration.

Protein	Function	Regulation	-LOG(*P*-value)	Difference
CccA	Cytochrome c550	↓	1.29	–0.25
CccB	Cytochrome c551	=	0.38	–0.07
CtaC	Cytochrome-c oxidase (subunit II)	↓	4.07	–0.76
CtaD	Cytochrome-c oxidase (subunit I)	↓	3.52	–0.77
CtaE	Cytochrome-c oxidase (subunit III)	↓	3.07	–0.65
CtaF	Cytochrome-c oxidase (subunit IV)	↓	3.24	–0.84
CtaG	Formation of functional cytochrome C-oxidase (caa3)	↓	3.04	–0.71
CydA	Cytochrome bd ubiquinol oxidase (subunit I)	↓	3.28	–0.87
CydB	Cytochrome bd ubiquinol oxidase (subunit II)	↓	3.52	–0.82
CydC	ABC transporter required for expression of cytochrome bd	↓	3.81	–0.97
CydD	ABC transporter required for expression of cytochrome bd	↓	3.18	–0.71
NarG	Nitrate reductase (α subunit)	OFF		
NarH	Nitrate reductase (β subunit)	OFF		
*Nar*I	Nitrate reductase (γ subunit)	OFF		
NarK	Nitrite extrusion protein	OFF		
Ndh	NADH dehydrogenase	↑	2.31	0.31
QcrA	Rieske factor, menaquinol:cytochrome c oxidoreductase (iron-sulfur subunit)	↓	2.97	–0.49
QcrB	Menaquinol:cytochrome c oxidoreductase (cytochrome b subunit)	↓	2.23	–0.27
QcrC	Menaquinol:cytochrome c oxidoreductase (cytochrome b/c subunit)	↓	2.72	–0.29
QoxA	Cytochrome aa3 quinol oxidase (subunit II)	=	0.62	–0.05
QoxB	Cytochrome aa3 quinol oxidase (subunit I)	=	0.29	–0.05
QoxD	Cytochrome aa3 quinol oxidase (subunit IV)	=	0.57	0.17
QoxD	Cytochrome aa3 quinol oxidase (subunit III)	=	0.04	0.03
ResA	Cytochrome c biogenesis	↓	3.42	–0.26
ResB	Cytochrome c biogenesis	↓	2.90	–0.25
ResC	Cytochrome c biogenesis	↓	1.83	–0.25
Sco	Maturation of cytochrome c oxidase caa3	↓	2.77	–0.37
YojN	Unknown	↑	3.39	0.39

*Absolute amounts for all quantified proteins and regulation of significantly changed abundances can be found in Supplementary Tables S5, S6, respectively.*

### The Response of Midi*Bacillus* Transporter Machinery to Production Stress

Protein translocation is an essential mechanism that establishes the movement of proteins onto the surface or into the extracellular milieu, thereby ensuring cell survival. The majority of bacterial proteins directed to cross the cell membrane are exported via the highly conserved Sec-dependent secretion pathway (Tjalsma et al., 2004). Upon induction of IsaA translocation via the Sec system, we found that the number of molecules per surface area dedicated to protein secretion increased by approximately 1% (Figure 4 and Supplementary Figures S2, S3), a somehow anticipated physiological response.

Protein secretion via the Sec pathway can be separated in three main stages: targeting, translocation, and folding and release (Tjalsma et al., 2004). This is a finely tuned process in which several constituents are involved. The translocation machinery consists of Sec A (motor protein), a heterotrimetric SecYEG complex (pore), SecDF (chimeric protein) (Bolhuis et al., 1998; van Wely et al., 2001; Tsirigotaki et al., 2016), YrbF (homolog of *E. coli* YajC) (Chen et al., 2015), and SpoIIIJ/YqjG (homolog of *E. coli* YidC) (Saller et al., 2011). SecA is localized in the cytosolic fraction, thereby not being considered for this study. As for the rest of the Sec translocation complex, our data show that the number of molecules that constitute each individual part of the complex does not significantly increase their abundance (0.19, 0.46, 0.71, 3.30, and 0.76 molecules/μm^2^ for SecG, SecY, SecDF, YrbF, and SpoIIJ, respectively) (Figure 5–Sec secretion system). Hence, even though induction of IsaA production accounts for an increase of 1% in the number of molecules dedicated to protein secretion via the Sec machinery (Supplementary Table S5), it does not lead to a significant increase in any particular component of the Sec translocation machinery.

Moreover, our results show an increase in abundance of the quality control protease HtrB (2.13-fold change, 0.01 molecules/μm^2^) (Figure 5–Sec secretion system and Supplementary Table S6), which is not considered to be significant as the HtrB has a standard deviation above 31% upon induction. The quality control proteases HtrA (not quantified in this study) and HtrB have the potential to assist in the folding or, when folding is not possible, degradation of misfolded proteins. They thereby ensure cell survival, as accumulation of misfolded protein in the cell envelope is lethal. Hence, our results provide evidence that IsaA induction leads the cells to activate the quality control machinery, hinting that these might be trying to cope with severe secretion stress. Nonetheless, we would like to remain that HtrB has a higher standard deviation and therefore we are careful when making biological assumptions. Another evidence supporting this observation is given by the accumulation of IsaA in the membrane fraction (Figures 3B, 5–IsaA), which would naturally trigger a secretion stress response in induced cells. In addition, a study performed by Hyyryläinen et al. (2005) showed that severe secretion stress lead to the significant induction of, not only *htrA* and *htrB*, but also *liaIHG*. Our results also show that LiaH is one of the “ON” proteins during IsaA induction (Table 1), corroborating the role of this protein in managing severe secretion stress.

The abundance patterns of the transporters encoded in midi*Bacillus* suggest that cells cope with the effects of secretion/folding stress caused by IsaA induction by compensatory up- or down-regulation of genes. For instance, our results show that a big part of the proteins annotated as ABC transporters are either OFF during IsaA induction, or they significantly decrease their abundance (Figure 5–ABC transporters), which corresponds to an overall decrease of ∼0.6% in the number of molecules dedicated to this cell function (Figure 4).

## Discussion

Minimal strains have emerged as one of the most attractive hosts for heterologous protein production as these can be tailored to harbor and produce virtually any protein of interest. However, in order to take advantage of the full capacity of such strains, it is essential to understand how protein abundances are modulated upon secretion. This applies especially to membrane proteins, as they constitute the major players in mediating the transport of secretory proteins. In this work, for the first time reported, we provide absolute membrane protein concentrations of a minimal strain of *B. subtilis* expressing the *S. aureus* major antigen–IsaA.

In this study, we were able to accurately quantify almost half of the predicted membrane proteome of midi*Bacillus*, which comprises a higher coverage than previous studies targeting the membrane proteome of the parental strain 168 (Dreisbach et al., 2008; Hahne et al., 2010). This is most likely due to the usage of faster and more sensitive mass spectrometers, more specific protocols for membrane proteome analysis, and the fact that midi*Bacillus* is expressing a higher percentage of its membrane proteome as it was designed to not harbor “unneeded” genes (Reuß et al., 2017).

Our data have shown that some membrane proteins are present in very low copy numbers (<1 molecules/μm^2^), suggesting that these proteins might be present in only a small subpopulation of the cells and are only expressed under very specific physiological conditions. This behavior has already been reported in literature as a way for the bacterial community to optimize its resources by differentiating into distinct cell types, having numerous metabolic processes activated at the same time, but not in all the same cells (Lopez et al., 2009). This is a phenomenon described as bistability (Dubnau and Losick, 2006). For instance, during the exponential growth phase, only a fraction of the cells was shown to express *sigD*, the gene for the sigma factor necessary for flagellar production, which results in heterogeneity in motility (Kearns and Losick, 2005). Also, when comparing our results with previously published data on global absolute protein quantification, we observed that there were striking differences between global protein concentrations (Maaß et al., 2011; Maaβ et al., 2014; Muntel et al., 2014; Antelo-Varela et al., 2019). Nevertheless, it should be noticed that this is the first global quantification study performed on a genome-reduced strain, as opposed to previous studies in which the parental wild-type strain of *B. subtilis* was used, making comparisons difficult. In addition, midi*Bacillus* has a 20 times larger surface area than its parental strain (40.9 and 1.8 μm^2^, respectively) (Antelo-Varela et al., 2019) (Supplementary Table S3). This might be a consequence of using a rich medium as opposed to a chemically defined one, or a deregulation in cell growth as a consequence of genome reduction (Reuß et al., 2017). For instance, when comparing the number of molecules/μm^2^ of the ATP synthase AtpF with the recently published study for absolute membrane protein quantification, we observe that wild-type *B. subtilis* produces ∼168 molecules/μm^2^, whereas midi*Bacillus* produces 1.6 molecules/μm^2^. This means that, in total, wild-type and midi*Bacillus* produce 302 and 65 molecules/cell of AtpF, respectively. Hence, it seems that the number of molecules per surface area is not proportional to cell size, as midi*Bacillus* maintains a relatively stable level of protein copy numbers. In a direct proportionality case, a strain with a 40.9 μm^2^ area would have instead 7.4 molecules/μm^2^ of AtpF.

Besides calculating absolute membrane protein molecules per cell surface area, our method also enabled to calculate accumulation rates of IsaA in the growth medium. Even though the Sec secretion apparatus of midi*Bacillus* is successfully secreting 2.41 molecules of IsaA/minute, there is still a considerable amount of IsaA accumulating in the membrane fraction–72 molecules/cell (Figure 3B). This accumulation of IsaA could be a hint of aberrant translocation by the Sec secretion machinery and/or unsuccessful processing of the signal peptide by signal peptidase (Bolhuis et al., 1999a). Moreover, it has been reported that precursors of IsaA tend to accumulate in the cell in a similar strain to the one used in this study (Suárez et al., 2019). Hence, one could expect that production of IsaA would improve by modulating the signal peptide to obtain an efficient signal peptidase cleavage site, as previously reported (von Heijne and Abrahmsèn, 1989; von Heijne, 1998). Furthermore, an increased expression of signal peptidases could also enhance the capacity of the secretion machinery (Tjalsma et al., 1998).

In our experiment, we have used 1% subtilin as it is the same concentration employed in the study that inspired our research. Nonetheless, we suggest that different concentrations of this inducer are worth exploring, as IsaA secretion could be improved by adjusting subtilin concentration.

Our results show reduced abundance levels of numerous proteins during IsaA production, which can be interpreted as an attempt of the cell to cope with the harmful effects of secretion stress. This is the case for proteins involved in respiration. Membrane-bound respiratory complexes constitute a major part of the membrane proteome. Thus, their regulation and biogenesis may impose a burden on the membrane, especially when stress is already in place. In fact, a study performed in *E. coli* has examined the connection between Cpx, the counterpart of the *B. subtilis* CssRS two-component regulatory system, with respiratory complexes (Hyyryläinen et al., 2001). Results of this study have shown that the Cpx response directly represses the transcription of these respiratory complexes conferring adaptation to stresses that compromise membrane homeostasis (Guest et al., 2017). This is confirmed by the present study, as our results also show that IsaA (72 molecules/cell) is being accumulated in the membrane fraction of midi*Bacillus* (Figure 3B). This will most likely alter the membrane equilibrium leading to decreased abundances of respiratory complexes.

Another result verifying that midi*Bacillus* is being subject to secretion stress is given by the significant increase of DltB, a protein involved in maintaining cell wall homeostasis by D-alanylation of teichoic acids. Nonetheless, there is evidence supporting the idea that inactivation of *dltB* and *dltD* (not quantified in our data) leads to increased secretion of specific proteins (Hyyryläinen et al., 2000). It has been inferred that the absence of D-alanylation leads to a localized increase in cation concentration at the membrane–wall interface (Hughes et al., 1973), a beneficial condition for protein folding. Hence, this would be a very interesting feature to explore in strains engineered for secretion of heterologous proteins, such as the one used in this study.

In addition, modulation of disulfide bond formation might be an additional strategy for higher yields in heterologous protein production, as this reaction is one the most important processes for the activity and stability of many exported proteins. However, little is known about disulfide bond formation in Gram positive bacteria (Reardon-Robinson and Ton-That, 2016), which turns optimization of this parameter into a quite cumbersome task for *B. subtilis*. However, a previously published study has suggested that the absence of the thiol-oxidoreductases BdbB or BdbC proteins resulted in significantly lower level of secretion of the two disulfide bonds containing alkaline phosphatase (PhoA) of *E. coli*, suggesting a role of these proteins in promoting extra-cytoplasmic protein folding (Bolhuis et al., 1999b).

Apparently, induced cells are dealing with severe secretion stress by rearranging their array of transporters in order to maintain a viable cell structure, one that permits a viable growth and survival. Moreover, one should bear in mind that midi*Bacillus* cells already show some growth deficiencies and present a bigger surface area. Hence, one might speculate that these phenotypes resultant of vast genome reduction might hamper the correct placement of integral membrane proteins in the cell surface, as the spatial orientation might be somehow disrupted. All these features should be considered when engineering microbial cell factories. Even though midi*Bacillus* is already quite efficient in secreting IsaA to the growth medium (Suárez et al., 2019), the results from our study show that there is still some room for improvement, as there is still some heterologous protein being accumulated in the cell membrane, causing the secretion stress response machinery to be induced.

## Conclusion

Biology is, without a doubt, one of the most complex fields of science as the slightest alteration has the potential to become catastrophic for the homeostasis of a biological system.

A minimal bacterial strain is highly attractive for the biotechnological sector, as all its remaining biological functions will be uniquely dedicated to the maintenance and production of high yields of heterologous proteins. However, what is the impact on the cell? How does the membrane proteome adapt upon overproduction of a “strange” protein?

In this study, we answer this question by applying a highly accurate method for absolute membrane protein quantification at the level of molecules/μm^2^. We were able to detect the physiological changes ensuing heterologous protein production, offering a clear visualization of alterations in protein patterns upon the onset of induction. Our results show that, even though IsaA production is being successfully induced, part of this protein is being accumulated in the membrane fraction, leading to a severe secretion stress response, and a reprogramming of the cell’s translocation machinery. Due to the exactness, the data described in this study can be implemented in predictive mathematical models aiming to understand the consequences of secretion stress and define the processes to be modulated. These data will be of the foremost importance to develop new-generation secretion systems for the biotechnological industry.

## Data Availability Statement

The datasets generated for this study have been deposited in the ProteomeXchange Consortium via the PRIDE partner repository with the dataset identifier PXD015496.

## Author Contributions

MA-V, SM, and DB conceived and designed the experiments. MA-V analyzed the data and wrote the manuscript. RA engineered the strain used in this study. MA-V, JB, MB-C, and TiS performed experiments. ThS performed the sample measurement. JD, SM, and DB supervised the project. SM, JD, and DB provided all necessary corrections. All authors have read and approved the manuscript.

## Conflict of Interest

The authors declare that the research was conducted in the absence of any commercial or financial relationships that could be construed as a potential conflict of interest.

## Abbreviations

AUC: area under the curve
FDR: false discovery rate
iBAQ algorithm: intensity-based absolute quantification algorithm
IsaA: immunodominant *Staphylococcus aureus* antigen A
LB medium: Lysogeny Broth medium
LC/MS: liquid chromatography–mass spectrometry
LTA: lipoteichoic acid
MS: mass spectrometry
PBPs: penicillin binding proteins
PG: peptidoglycan
SDS: sodium dodecyl sulfate
SDS-PAGE: SDS-polyacrylamide gel electrophoresis
SRM: selected reaction monitoring
TE buffer: Tris EDTA buffer
TEAB buffer: tetraethylammonium bromide buffer
TMD: transmembrane domain
UPS2: Universal Proteomics Standard.

## References

[B1] Abràmofff M. D. Magalhães P. J. Ram S. J. (2005). Image processing with ImageJ Part II. *Biophotonics Int.* 11 36–43. 10.1117/1.3589100 21721809

[B2] Agapakis C. M. Ducat D. C. Boyle P. M. Wintermute E. H. Way J. C. Silver P. A. (2010). Insulation of a synthetic hydrogen metabolism circuit in bacteria. *J. Biol. Eng.* 4:3. 10.1186/1754-1611-4-3 20184755 PMC2847965

[B3] Aggarwal K. Lee K. H. (2003). Functional genomics and proteomics as a foundation for systems biology. *Brief. Funct. Genomics Proteomics* 2 175–184. 10.1093/bfgp/2.3.175 15239921

[B4] Antelo-Varela M. Bartel J. Quesada-Ganuza A. Appel K. Bernal-Cabas M. Sura T. (2019). Ariadne’s thread in the analytical labyrinth of membrane proteins: integration of targeted and shotgun proteomics for global absolute quantification of membrane proteins. *Anal. Chem.* 91 11972–11980. 10.1021/acs.analchem.9b02869 31424929

[B5] Bolhuis A. Broekhuizen C. P. Sorokin A. van Roosmalen M. L. Venema G. Bron S. (1998). SecDF of *Bacillus subtilis*, a molecular siamese twin required for the efficient secretion of proteins^∗^. *J. Biol. Chem.* 273 21217–21224. 10.1074/jbc.273.33.21217 9694879

[B6] Bolhuis A. Tjalsma H. Smith H. E. De Jong A. Meima R. Venema G. (1999a). Evaluation of bottleneck in the late stages of protein secretion in *Bacillus subtilis*. *Appl. Environ. Microbiol.* 65 2934–2941. 10388686 10.1128/aem.65.7.2934-2941.1999 PMC91439

[B7] Bolhuis A. Venema G. Quax W. J. Bron S. van Dijl J. M. (1999b). Functional analysis of paralogous thiol-disulfide oxidoreductases in *Bacillus subtilis*^∗^. *J. Biol. Chem.* 274 24531–24538. 10.1074/jbc.274.35.24531 10455116

[B8] Bonn F. Bartel J. Büttner K. Hecker M. Otto A. Becher D. (2014). Picking vanished proteins from the void: How to collect and ship/share extremely dilute proteins in a reproducible and highly efficient manner. *Anal. Chem.* 86 7421–7427. 10.1021/ac501189j 24987932

[B9] Bradford M. M. (1976). A rapid and sensitive method for the quantitation of microgram quantities of protein utilizing the principle of protein-dye binding. *Anal. Biochem.* 72 248–254. 10.1016/0003-2697(76)90527-3 942051

[B10] Chen J. Fu G. Gai Y. Zheng P. Zhang D. Wen J. (2015). Combinatorial Sec pathway analysis for improved heterologous protein secretion in *Bacillus subtilis*: identification of bottlenecks by systematic gene overexpression. *Microb. Cell Fact.* 14:92. 10.1186/s12934-015-0282-9 26112883 PMC4482152

[B11] Chen Z. Wilmanns M. Zeng A. P. (2010). Structural synthetic biotechnology: from molecular structure to predictable design for industrial strain development. *Trends Biotechnol.* 28 534–542. 10.1016/j.tibtech.2010.07.004 20727604

[B12] Cox J. Neuhauser N. Michalski A. Scheltema R. A. Olsen J. V. Mann M. (2011). Andromeda: a peptide search engine integrated into the MaxQuant environment. *J. Proteome Res.* 10 1794–1805. 10.1021/pr101065j 21254760

[B13] Deutsch E. W. Csordas A. Sun Z. Jarnuczak A. Perez-Riverol Y. Ternent T. (2017). The ProteomeXchange consortium in 2017?: supporting the cultural change in proteomics public data deposition. *Nucleic Acids Res.* 45 1100–1106. 10.1093/nar/gkw936 27924013 PMC5210636

[B14] Dreisbach A. Otto A. Becher D. Hammer E. Teumer A. Gouw J. W. (2008). Monitoring of changes in the membrane proteome during stationary phase adaptation of *Bacillus subtilis* using in vivo labeling techniques. *Proteomics* 8 2062–2076. 10.1002/pmic.200701081 18491319

[B15] Dubnau D. Losick R. (2006). Bistability in bacteria. *Mol. Microbiol.* 61 564–572. 10.1111/j.1365-2958.2006.05249.x 16879639

[B16] Eiamphungporn W. Helmann J. (2008). The *Bacillus subtilis* σM regulon and its contribution to cell envelope stress responses. *Mol. Microbiol.* 67 830–848. 10.1038/jid.2014.371 18179421 PMC3025603

[B17] Elowitz M. B. Leibler S. (2000). A synthetic oscillatory network repressilator. *Nature* 403 335–338. 10.1038/35002125 10659856

[B18] Eymann C. Dreisbach A. Albrecht D. Bernhardt J. Becher D. Gentner S. (2004). A comprehensive proteome map of growing *Bacillus subtilis* cells. *Proteomics* 4 2849–2876. 10.1002/pmic.200400907 15378759

[B19] Fredens J. Wang K. de la Torre D. Funke L. F. H. Robertson W. E. Christova Y. (2019). Total synthesis of *Escherichia coli* with a recoded genome. *Nature* 569 514–518. 10.1038/s41586-019-1192-5 31092918 PMC7039709

[B20] García-Pérez A. N. de Jong A. Junker S. Becher D. Chlebowicz M. A. Duipmans J. C. (2018). From the wound to the bench: exoproteome interplay between wound-colonizing *Staphylococcus aureus* strains and co-existing bacteria. *Virulence* 9 363–378. 10.1080/21505594.2017.1395129 29233035 PMC5955179

[B21] Gardner T. S. Cantor C. R. Collins J. J. (2000). Construction of a genetic toggle switch in *Escherichia coli*. *Nature* 403 339–342. 10.1038/35002131 10659857

[B22] Gibson D. G. Glass J. I. Lartigue C. Noskov V. N. Algire M. A. Benders G. A. (2010). Creation of a bacterial cell controlled by a chemically synthesized genome. *Science* 329 52–56. 10.1126/science.1190719 20488990

[B23] Gilbert C. Howarth M. Harwood C. R. Ellis T. (2017). Extracellular self-assembly of functional and tunable protein conjugates from *Bacillus subtilis*. *ACS Synth. Biol.* 6 957–967. 10.1021/acssynbio.6b00292 28230977

[B24] Glick B. (1995). Metabolic load and heterologous gene expression. *Biotechnol. Adv.* 13 247–261. 10.1016/0734-9750(95)00004-a 14537822

[B25] Goosens V. J. Otto A. Glasner C. Monteferrante C. C. Van Der Ploeg R. Hecker M. (2013). Novel twin-arginine translocation pathway-dependent phenotypes of *Bacillus subtilis* unveiled by quantitative proteomics. *J. Proteome Res.* 12 796–807. 10.1021/pr300866f 23256564

[B26] Guest R. L. Wang J. Wong J. L. Raivio T. L. (2017). A bacterial stress response regulates respiratory protein complexes to control envelope stress adaptation. *J. Bacteriol.* 199:e00153-17. 10.1016/j.jneumeth.2010.07.015 28760851 PMC5637174

[B27] Hahne H. Mäder U. Otto A. Bonn F. Steil L. Bremer E. (2010). A comprehensive proteomics and transcriptomics analysis of *Bacillus subtilis* salt stress adaptation. *J. Bacteriol.* 192 870–882. 10.1128/JB.01106-09 19948795 PMC2812467

[B28] Hughes A. H. Hancock I. C. Baddiley J. (1973). The function of teichoic acids in cation control in bacterial membranes. *Biochem. J.* 132 83–93. 10.1042/bj1320083 4722902 PMC1177562

[B29] Hutchison C. A. Chuang R. Y. Noskov V. N. Assad-Garcia N. Deerinck T. J. Ellisman M. H. (2016). Design and synthesis of a minimal bacterial genome. *Science* 351:aad6253. 10.1126/science.aad6253 27013737

[B30] Hyyryläinen H.-L. Bolhuis A. Darmon E. Muukkonen L. Koski P. Vitikainen M. (2001). A novel two-component regulatory system in *Bacillus subtilis* for the survival of severe secretion stress. *Mol. Microbiol.* 41 1159–1172. 10.1046/j.1365-2958.2001.02576.x 11555295

[B31] Hyyryläinen H. L. Marciniak B. C. Dahncke K. Pietiäinen M. Courtin P. Vitikainen M. (2010). Penicillin-binding protein folding is dependent on the PrsA peptidyl-prolyl cis-trans isomerase in *Bacillus subtilis*. *Mol. Microbiol.* 77 108–127. 10.1111/j.1365-2958.2010.07188.x 20487272

[B32] Hyyryläinen H. L. Sarvas M. Kontinen V. P. (2005). Transcriptome analysis of the secretion stress response of *Bacillus subtilis*. *Appl. Microbiol. Biotechnol.* 67 389–396. 10.1007/s00253-005-1898-1 15856219

[B33] Hyyryläinen H. L. Vitikainen M. Thwaite J. Wu H. Sarvas M. Harwood C. R. (2000). D-alanine substitution of teichoic acids as a modulator of protein folding and stability at the cytoplasmic membrane/cell wall interface of *Bacillus subtilis*. *J. Biol. Chem.* 275 26696–26703. 10.1074/jbc.M003804200 10871614

[B34] Jongbloed J. D. H. Grieger U. Antelmann H. Hecker M. Nijland R. Bron S. (2004). Two minimal Tat translocases in *Bacillus*. *Mol. Microbiol.* 54 1319–1325. 10.1111/j.1365-2958.2004.04341.x 15554971

[B35] Kearns D. B. Losick R. (2005). Cell population heterogeneity during growth of *Bacillus subtilis*. *Genes Dev.* 19 3083–3094. 10.1101/gad.1373905.mediated 16357223 PMC1315410

[B36] Krishnappa L. Dreisbach A. Otto A. Goosens V. J. Cranenburgh R. M. Harwood C. R. (2013). Extracytoplasmic proteases determining the cleavage and release of secreted proteins, lipoproteins, and membrane proteins in *Bacillus subtilis*. *J. Proteome Res.* 12 4101–4110. 10.1021/pr400433h 23937099

[B37] Lee S. Y. Lee D.-Y. Kim T. Y. (2005). Systems biotechnology for strain improvement. *Trends Biotechnol.* 23 349–358. 10.1016/j.tibtech.2005.05.003 15923052

[B38] Lee S. Y. Mattanovich D. Villaverde A. (2012). Systems metabolic engineering, industrial biotechnology and microbial cell factories. *Microb. Cell Fact.* 11:156. 10.1186/1475-2859-11-156 23232052 PMC3539922

[B39] Lopez D. Vlamakis H. Kolter R. (2009). Generation of multiple cell types in *Bacillus subtilis*. *FEMS Microbiol. Rev.* 33 152–163. 10.1111/j.1574-6976.2008.00148.x 19054118

[B40] Maaß S. Sievers S. Zühlke D. Kuzinski J. Sappa P. K. Muntel J. (2011). Efficient, global-scale quantification of absolute protein amounts by integration of targeted mass spectrometry and two-dimensional gel-based proteomics. *Anal. Chem.* 83 2677–2684. 10.1021/ac1031836 21395229

[B41] Maaβ S. Wachlin G. Bernhardt J. Eymann C. Fromion V. Riedel K. (2014). Highly precise quantification of protein molecules per cell during stress and starvation responses in *Bacillus subtilis*. *Mol. Cell. Proteomics* 13 2260–2276. 10.1074/mcp.M113.035741 24878497 PMC4159648

[B42] MacLean B. Tomazela D. M. Shulman N. Chambers M. Finney G. L. Frewen B. (2010). Skyline: an open source document editor for creating and analyzing targeted proteomics experiments. *Bioinformatics* 26 966–968. 10.1093/bioinformatics/btq054 20147306 PMC2844992

[B43] Mäder U. Schmeisky A. G. Flórez L. A. Stülke J. (2012). SubtiWiki – A comprehensive community resource for the model organism *Bacillus subtilis*. *Nucleic Acids Res.* 40 1278–1287. 10.1093/nar/gkr923 22096228 PMC3245094

[B44] Makino T. Skretas G. Georgiou G. (2011). Strain engineering for improved expression of recombinant proteins in bacteria. *Microb. Cell Fact.* 10:32. 10.1186/1475-2859-10-32 21569582 PMC3120638

[B45] Malmström J. Beck M. Schmidt A. Lange V. Deutsch E. W. Aebersold R. (2009). Proteome-wide cellular protein concentrations of the human pathogen *Leptospira interrogans*. *Nature* 460 762–765. 10.1038/nature08184 19606093 PMC2723184

[B46] Muntel J. Fromion V. Goelzer A. Maaβ S. Mäder U. Büttner K. (2014). Comprehensive absolute quantification of the cytosolic proteome of *Bacillus subtilis* by data independent, parallel fragmentation in liquid chromatography/mass spectrometry (LC/MSE). *Mol. Cell. Proteomics* 13 1008–1019. 10.1074/mcp.M113.032631 24696501 PMC3977180

[B47] Neuhaus F. C. Baddiley J. (2003). A continuum of anionic charge: structures and functions of D-alanyl-teichoic acids in gram-positive bacteria. *Microbiol. Mol. Biol. Rev.* 67 686–723. 10.1128/mmbr.67.4.686-723.2003 14665680 PMC309049

[B48] Palva I. Lehtovaara P. Kääriäinen L. Sibakov M. Cantell K. Schein C. H. (1983). Secretion of interferon by *Bacillus subtilis*. *Gene* 22 229–235. 10.7868/s0026365614040119 6307823

[B49] Papanastasiou M. Aivaliotis M. Karamanou S. Koukaki M. Sardis M. F. Orfanoudaki G. (2012). The *Escherichia coli* peripheral inner membrane proteome. *Mol. Cell. Proteomics* 12 599–610. 10.1074/mcp.m112.024711 23230279 PMC3591654

[B50] Parekh S. Vinci V. A. Strobel R. J. (2000). Improvement of microbial strains and fermentation processes. *Appl. Microbiol. Biotechnol.* 54 287–301. 10.1007/s002530000403 11030563

[B51] Percy M. G. Gründling A. (2014). Lipoteichoic acid synthesis and function in gram-positive bacteria. *Annu. Rev. Microbiol.* 68 81–100. 10.1146/annurev-micro-091213-112949 24819367

[B52] Perez-Riverol Y. Csordas A. Bai J. Bernal-Llinares M. Hewapathirana S. Kundu D. J. (2019). The PRIDE database and related tools and resources in 2019: Improving support for quantification data. *Nucleic Acids Res.* 47 D442–D450. 10.1093/nar/gky1106 30395289 PMC6323896

[B53] Pohl S. Harwood C. R. (2010). Heterologous protein secretion by *Bacillus* species from the cradle to the grave. *Adv. Appl. Microbiol.* 73 21–25. 10.1016/S0065-2164(10)73001-X 20800757

[B54] Rahmer R. Heravi K. M. Altenbuchner J. (2015). Construction of a super-competent *Bacillus subtilis* 168 using the PmtlA-comKS inducible cassette. *Front. Microbiol.* 6:1431. 10.3389/fmicb.2015.01431 26732353 PMC4685060

[B55] Reardon-Robinson M. E. Ton-That H. (2016). Disulfide-bond-forming pathways in Gram-positive bacteria. *J. Bacteriol.* 198 746–754. 10.1128/JB.00769-15 26644434 PMC4810614

[B56] Reuß D. R. Altenbuchner J. Mäder U. Rath H. Ischebeck T. Sappa P. K. (2017). Large-scale reduction of the *Bacillus subtilis* genome: consequences for the transcriptional network, resource allocation, and metabolism. *Genome Res.* 27 289–299. 10.1101/gr.215293.116 27965289 PMC5287234

[B57] Ro D. K. Paradise E. M. Quellet M. Fisher K. J. Newman K. L. Ndungu J. M. (2006). Production of the antimalarial drug precursor artemisinic acid in engineered yeast. *Nature* 440 940–943. 10.1038/nature04640 16612385

[B58] Saller M. J. Otto A. Berrelkamp-lahpor G. A. Becher D. Hecker M. Driessen A. J. M. (2011). *Bacillus subtilis* YqjG is required for genetic competence development. *Proteomics* 11 270–282. 10.1002/pmic.201000435 21204254

[B59] Schallmey M. Singh A. Ward O. P. (2004). Developments in the use of *Bacillus* species for industrial production. *Can. J. Microbiol.* 50 1–17. 10.1139/w03-076 15052317

[B60] Schwanhäusser B. Busse D. Li N. Dittmar G. Schuchhardt J. Wolf J. (2011). Global quantification of mammalian gene expression control. *Nature* 473 337–342. 10.1038/nature10098 21593866

[B61] Suárez R. A. Stülke J. Van Dijl J. M. (2019). Less is more: toward a genome-reduced *Bacillus* cell factory for “difficult Proteins”. *ACS Synth. Biol.* 8 99–108. 10.1021/acssynbio.8b00342 30540431 PMC6343112

[B62] Szybalski W. (1974). “*In vivo* and *in vitro* initiation of transcription,” in *Control of Gene Expression*, eds Kohn A. Shatkay A. (Boston, MA: Springer), 23–24. 10.1007/978-1-4684-3246-6_3 4837680

[B63] Tjalsma H. Antelmann H. Jongbloed J. D. H. Braun P. G. Darmon E. Dorenbos R. (2004). Proteomics of protein secretion by *Bacillus subtilis*: separating the ‘Secrets’of the secretome. *Microbiol. Mol. Biol. Rev.* 68 207–233. 10.1128/MMBR.68.2.207-233.2004 15187182 PMC419921

[B64] Tjalsma H. Bolhuis A. van Roosmalen M. L. Wiegert T. Schumann W. Broekhuizen C. P. (1998). Functional analysis of the secretory precursor processing machinery of *Bacillus subtilis*: identification of a eubacterial homolog of archaeal and eukaryotic signal peptidases. *Genes Dev.* 12 2318–2331. 10.1101/gad.12.15.2318 9694797 PMC317044

[B65] Tsirigotaki A. De Geyter J. Šoštaric N. Economou A. Karamanou S. (2016). Protein export through the bacterial Sec pathway. *Nat. Rev. Microbiol.* 15 21–36. 10.1038/nrmicro.2016.161 27890920

[B66] Tusnády G. E. Simon I. (2001). The HMMTOP transmembrane topology prediction server. *Bioinformatics* 17 849–850. 10.1093/bioinformatics/17.9.849 11590105

[B67] Tyanova S. Temu T. Cox J. (2016a). The MaxQuant computational platform for mass spectrometry-based shotgun proteomics. *Nat. Protoc.* 11 2301–2319. 10.1038/nprot.2016.136 27809316

[B68] Tyanova S. Temu T. Sinitcyn P. Carlson A. Hein M. Y. Geiger T. (2016b). The Perseus computational platform for comprehensive analysis of (prote)omics data. *Nat. Methods* 13 731–740. 10.1038/nmeth.3901 27348712

[B69] van den Berg S. Bonarius H. P. J. van Kessel K. P. M. Elsinga G. S. Kooi N. Westra H. (2015). A human monoclonal antibody targeting the conserved *Staphylococcal* antigen IsaA protects mice against *Staphylococcus aureus* bacteremia. *Int. J. Med. Microbiol.* 305 55–64. 10.1016/j.ijmm.2014.11.002 25466204

[B70] van Dijl J. M. Hecker M. (2013). *Bacillus subtilis*: from soil bacterium to super-secreting cell factory. *Microb. Cell Fact.* 12:3. 10.1186/1475-2859-12-3 23311580 PMC3564730

[B71] van Wely K. H. M. Swaving J. Freudl R. Driessen A. J. M. (2001). Translocation of proteins across the cell envelope of Gram-positive bacteria. *FEMS Microbiol. Rev.* 25 437–454. 10.1111/j.1574-6976.2001.tb00586.x 11524133

[B72] Venetz J. E. Del Medico L. Wölfle A. Schächle P. Bucher Y. Appert D. (2019). Chemical synthesis rewriting of a bacterial genome to achieve design flexibility and biological functionality. *Proc. Natl. Acad. Sci. U.S.A.* 116 8070–8079. 10.1073/pnas.1818259116 30936302 PMC6475421

[B73] von Heijne G. (1998). Life and death of a signal peptide. *Nature* 396 111–113. 10.1080/08940889508602814 9823886

[B74] von Heijne G. Abrahmsèn L. (1989). Species-specific variation in signal peptide design. *FEBS Lett.* 244 439–446. 10.1016/0014-5793(89)80579-4 2646153

[B75] Westers L. Westers H. Quax W. J. (2004). *Bacillus subtilis* as cell factory for pharmaceutical proteins: a biotechnological approach to optimize the host organism. *Biochim. Biophys. Acta Mol. Cell Res.* 1694 299–310. 10.1016/j.bbamcr.2004.02.011 15546673

[B76] Wiśniewski J. R. Rakus D. (2014). Multi-enzyme digestion FASP and the ‘Total Protein Approach’-based absolute quantification of the *Escherichia coli* proteome. *J. Proteomics* 109 322–331. 10.1016/j.jprot.2014.07.012 25063446

[B77] Zweers J. C. Barák I. Becher D. Driessen A. J. Hecker M. Kontinen V. P. (2008). Towards the development of *Bacillus subtilis* as a cell factory for membrane proteins and protein complexes. *Microb. Cell Fact.* 7:10. 10.1186/1475-2859-7-10 18394159 PMC2323362

