# Supplementary material for: Membrane Modulation of Super-Secreting “midiBacillus” Expressing the Major Staphylococcus aureus Antigen – A Mass-Spectrometry-Based Absolute Quantification Approach
Source: Front Bioeng Biotechnol. 2020 Feb 28;8:143. doi: 10.3389/fbioe.2020.00143 (PMC7059095; doi:10.3389/fbioe.2020.00143)
Supplement: Supplementary file 1 [file Data_Sheet_1.PDF]

Membrane modulation of super-secreting “midi*Bacillus*” expressing the major *Staphylococcus aureus* antigen – a mass-spectrometry based absolute quantification approach.

Minia Antelo-Varela[1], Rocío Aguilar Suárez[2], Jürgen Bartel[1], Margarita Bernal-Cabas[2], Tim Stobernack[2], Thomas Sura[1], Jan Maarten van Dijl[2], Sandra Maaß[1], Dörte Becher[1*].

Supplementary figures and tables

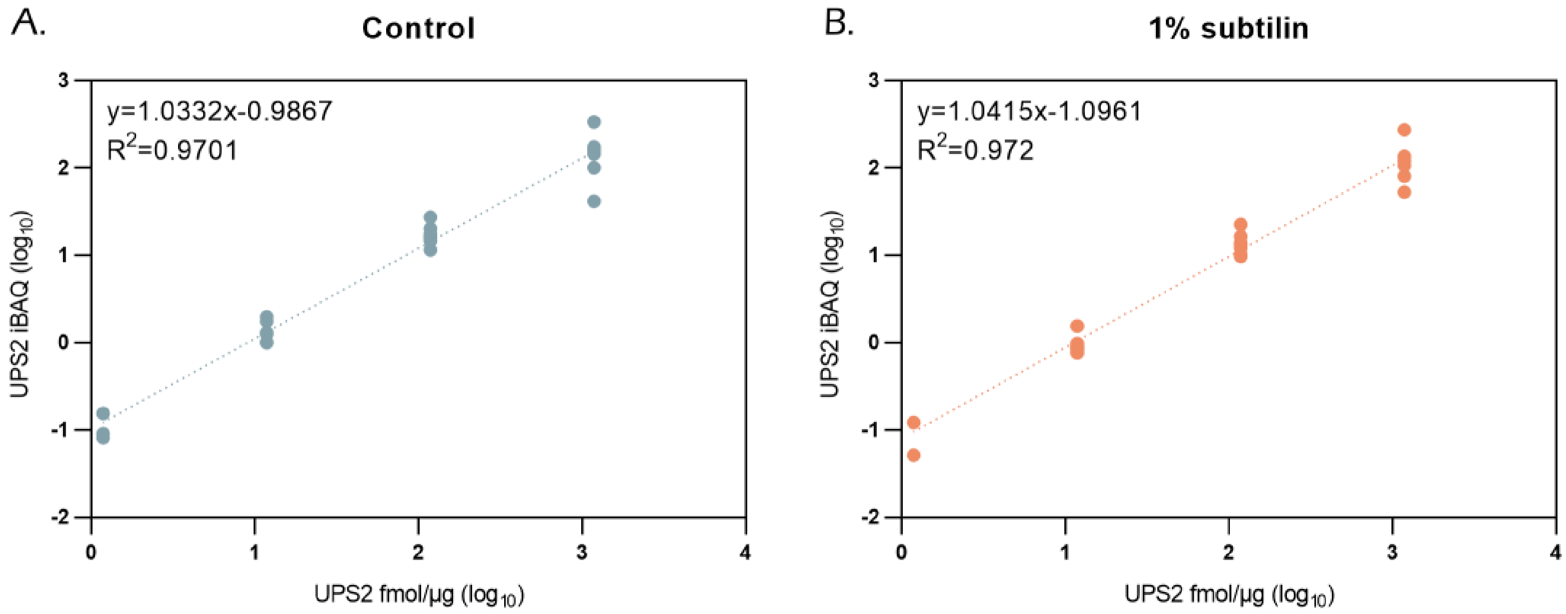

Supplementary Figure S1. Linear calibration curve of UPS2 standards resultant of shotgun MS for (A) control and (B) 1% subtilin induction conditions. iBAQ intensities were plotted against known concentration of UPS2 standards in order to derive a linear regression equation. Slope and intercept for (A) control and (B) 1% subtilin induction conditions were used to calculate absolute protein abundances. Four orders of magnitude were accurately quantified with an r2 of 0.9701 and 0.972 for (A) control and (B) 1% subtilin induction, respectively.

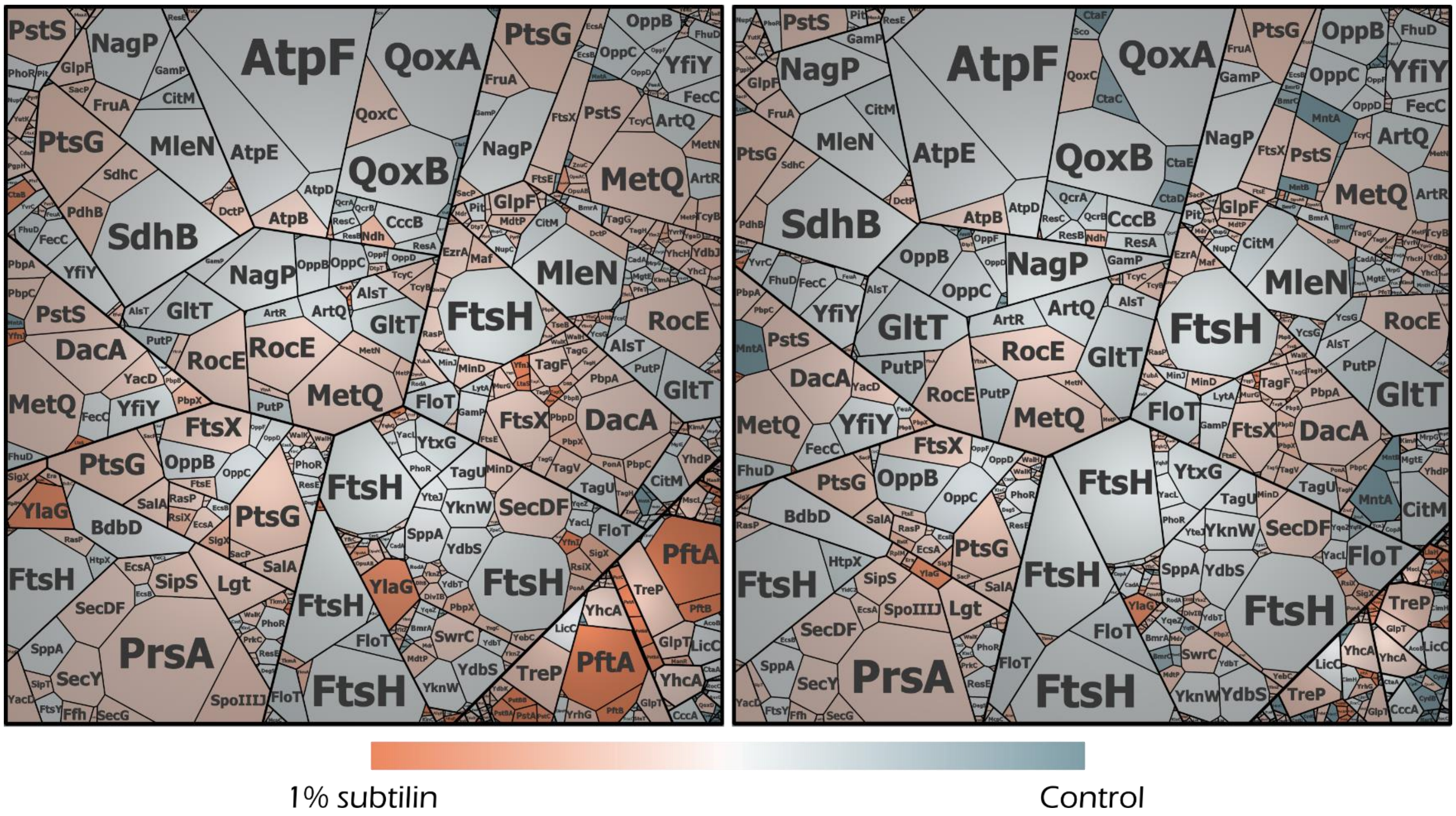

Supplementary Figure S2. Voronoi treemaps illustrating copy numbers/µm2 of membrane proteins in induction and control conditions. Proteins quantified via shotgun MS are displayed as single cells, which are functionally clustered according to the SubtiWiki gene orthology [1]. A protein appearing more than once is included in more than one functional category. Cell size corresponds to protein abundance and colour code indicates abundance in each of the measured conditions: grey – proteins more abundant in control conditions; orange – protein more abundant in induction conditions; white – no difference in protein abundance.

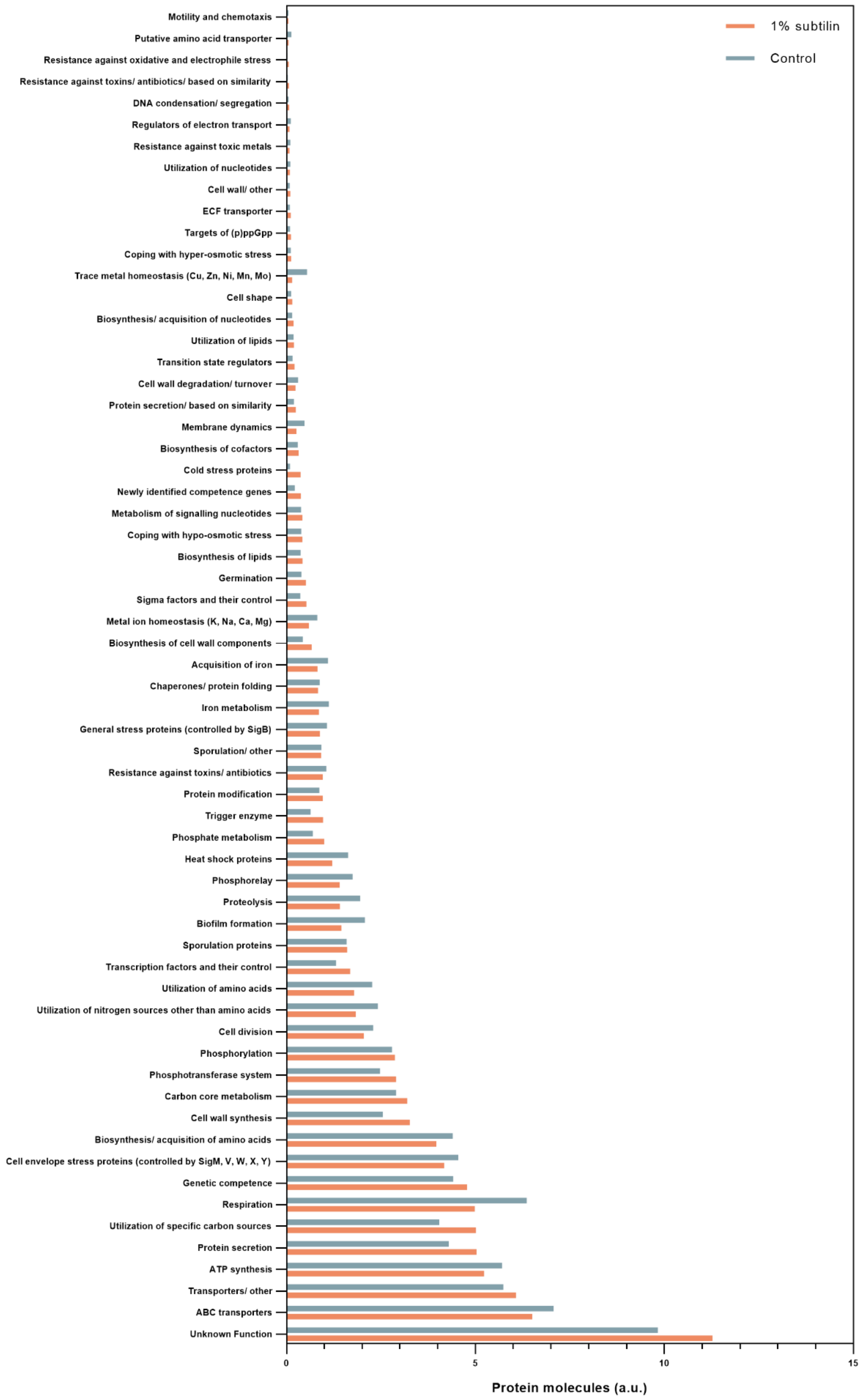

Supplementary Figure S3. Assignment of membrane protein copy number per cell surface to a

specific subcellular function. Data was assigned to a specific function according to SubtiWiki [1] gene categorization. Data is clustered in one single hierarchical level. Different data attribution between tested conditions are indicated by a colour code: grey – control and orange – 1% subtilin induction.

Supplementary Table S1. Strains and plasmids used in this study.

Strain or Plasmid	Characteristics	Reference
B. subtilis IIG-Bs27-47-24	Genome-reduced strain	Reuß *et al*, 2017[2]
B. subtilis ATCC 6633	Subtilin production	ATCC collection
pNZ8900	*amyE*::*spaRK*, Km^R	Bongers *et al*, 2005[3]
pRAG3::IsaA	SURE expression vector; SPxynA; IsaA-His6, Em^R	Aguilar *et al*, 2019[4]

Km^R: Kanamycin resistance
Em^R: Erythromycin resistance

Supplementary Table S1. Table listing the strains and plasmids used in this study and the relevant properties of each of them. Reference column refers to the study in which the respective strain or plasmid was constructed. Antibiotic resistance abbreviations are listed below the table.

Supplementary Table S2. Sequences of MS-quantified peptides of midi*Bacillus* and their optimized SRM acquisition parameters

protein	peptide sequence	Q1 mass	Q3 mass	ion	CE volts
QcrA	FALDPVL**K**	455.778028	763.480365	y7	16.5
QcrA	FALDPVL**K**	455.778028	692.443252	y6	16.5
QcrA	FALDPVL**K**	455.778028	579.359188	y5	16.5
QcrA	FALDPVL**K**	455.778028	464.332245	y4	16.5
QcrA	FALDPVL**K**	455.778028	382.243821	y7	16.5
QcrA	FALDPVL**K**	455.778028	346.725264	y6	16.5
QcrA	FALDPVL**K**	455.778028	290.183232	y5	16.5
QcrA	FALDPVL**K**	455.778028	232.66976	y4	16.5
QcrA	FALDPVLK	451.770928	755.466166	y7	16.5
QcrA	FALDPVLK	451.770928	684.429053	y6	16.5
QcrA	FALDPVLK	451.770928	571.344989	y5	16.5
QcrA	FALDPVLK	451.770928	456.318046	y4	16.5
QcrA	FALDPVLK	451.770928	378.236721	y7	16.5
QcrA	FALDPVLK	451.770928	342.718164	y6	16.5
QcrA	FALDPVLK	451.770928	286.176132	y5	16.5
QcrA	FALDPVLK	451.770928	228.662661	y4	16.5
QcrA	INQVDAWYESEES**R**	868.393507	1281.525755	y10	28.8
QcrA	INQVDAWYESEES**R**	868.393507	1166.498812	y9	28.8
QcrA	INQVDAWYESEES**R**	868.393507	1095.461699	y8	28.8
QcrA	INQVDAWYESEES**R**	868.393507	909.382386	y7	28.8
QcrA	INQVDAWYESEES**R**	868.393507	746.319057	y6	28.8
QcrA	INQVDAWYESEES**R**	868.393507	617.276464	y5	28.8
QcrA	INQVDAWYESEESR	863.389373	1271.517486	y10	28.8
QcrA	INQVDAWYESEESR	863.389373	1156.490543	y9	28.8
QcrA	INQVDAWYESEESR	863.389373	1085.45343	y8	28.8
QcrA	INQVDAWYESEESR	863.389373	899.374117	y7	28.8
QcrA	INQVDAWYESEESR	863.389373	736.310788	y6	28.8
QcrA	INQVDAWYESEESR	863.389373	607.268195	y5	28.8
QcrA	DGTNVPGTPPLAPLDHYEQEV**K**	795.731927	1336.662306	y11	32.4
QcrA	DGTNVPGTPPLAPLDHYEQEV**K**	795.731927	1265.625192	y10	32.4
QcrA	DGTNVPGTPPLAPLDHYEQEV**K**	795.731927	940.461421	y7	32.4
QcrA	DGTNVPGTPPLAPLDHYEQEV**K**	795.731927	949.99054	y17	32.4
QcrA	DGTNVPGTPPLAPLDHYEQEV**K**	795.731927	822.429587	y14	32.4
QcrA	DGTNVPGTPPLAPLDHYEQEV**K**	795.731927	256.151932	y4	32.4
QcrA	DGTNVPGTPPLAPLDHYEQEVK	793.060528	1328.648107	y11	32.4
QcrA	DGTNVPGTPPLAPLDHYEQEVK	793.060528	1257.610993	y10	32.4
QcrA	DGTNVPGTPPLAPLDHYEQEVK	793.060528	932.447222	y7	32.4
QcrA	DGTNVPGTPPLAPLDHYEQEVK	793.060528	945.98344	y17	32.4
QcrA	DGTNVPGTPPLAPLDHYEQEVK	793.060528	818.422487	y14	32.4
QcrA	DGTNVPGTPPLAPLDHYEQEVK	793.060528	252.144832	y4	32.4

QcrA	DGFLYLGK	460.75201	805.469801	y7	16.6
QcrA	DGFLYLGK	460.75201	748.448337	y6	16.6
QcrA	DGFLYLGK	460.75201	601.379923	y5	16.6
QcrA	DGFLYLGK	460.75201	488.295859	y4	16.6
QcrA	DGFLYLGK	460.75201	374.727807	y6	16.6
QcrA	DGFLYLGK	456.74491	797.455602	y7	16.6
QcrA	DGFLYLGK	456.74491	740.434138	y6	16.6
QcrA	DGFLYLGK	456.74491	593.365724	y5	16.6
QcrA	DGFLYLGK	456.74491	480.28166	y4	16.6
QcrA	DGFLYLGK	456.74491	370.720707	y6	16.6
YwbN	GFLSGGK	337.191588	616.354437	y6	12.9
YwbN	GFLSGGK	337.191588	469.286023	y5	12.9
YwbN	GFLSGGK	337.191588	356.201959	y4	12.9
YwbN	GFLSGGK	337.191588	308.680856	y6	12.9
YwbN	GFLSGGK	337.191588	235.146649	y5	12.9
YwbN	GFLSGGK	337.191588	178.604617	y4	12.9
YwbN	GFLSGGK	333.184489	608.340238	y6	12.9
YwbN	GFLSGGK	333.184489	461.271824	y5	12.9
YwbN	GFLSGGK	333.184489	348.18776	y4	12.9
YwbN	GFLSGGK	333.184489	304.673757	y6	12.9
YwbN	GFLSGGK	333.184489	231.13955	y5	12.9
YwbN	GFLSGGK	333.184489	174.597518	y4	12.9
YwbN	DQEDTFGR	489.213555	862.392891	y7	17.4
YwbN	DQEDTFGR	489.213555	734.334313	y6	17.4
YwbN	DQEDTFGR	489.213555	605.29172	y5	17.4
YwbN	DQEDTFGR	489.213555	490.264777	y4	17.4
YwbN	DQEDTFGR	489.213555	431.700083	y7	17.4
YwbN	DQEDTFGR	489.213555	367.670795	y6	17.4
YwbN	DQEDTFGR	484.20942	852.384622	y7	17.4
YwbN	DQEDTFGR	484.20942	724.326044	y6	17.4
YwbN	DQEDTFGR	484.20942	595.283451	y5	17.4
YwbN	DQEDTFGR	484.20942	480.256508	y4	17.4
YwbN	DQEDTFGR	484.20942	426.695949	y7	17.4
YwbN	DQEDTFGR	484.20942	362.66666	y6	17.4
YwbN	LNQIPSNSHVSLAK	505.950489	1047.567283	y10	21.4
YwbN	LNQIPSNSHVSLAK	505.950489	950.514519	y9	21.4
YwbN	LNQIPSNSHVSLAK	505.950489	525.348623	y5	21.4
YwbN	LNQIPSNSHVSLAK	505.950489	426.280209	y4	21.4
YwbN	LNQIPSNSHVSLAK	505.950489	580.829312	y11	21.4
YwbN	LNQIPSNSHVSLAK	505.950489	524.28728	y10	21.4
YwbN	LNQIPSNSHVSLAK	503.27909	1039.553084	y10	21.4
YwbN	LNQIPSNSHVSLAK	503.27909	942.50032	y9	21.4
YwbN	LNQIPSNSHVSLAK	503.27909	517.334424	y5	21.4
YwbN	LNQIPSNSHVSLAK	503.27909	418.26601	y4	21.4

YwbN	LNQIPSNSHVSLAK	503.27909	576.822212	y11	21.4
YwbN	LNQIPSNSHVSLAK	503.27909	520.28018	y10	21.4
YwbN	GEYIAQ**R**	423.720819	789.412898	y6	15.5
YwbN	GEYIAQ**R**	423.720819	660.370305	y5	15.5
YwbN	GEYIAQ**R**	423.720819	497.306976	y4	15.5
YwbN	GEYIAQ**R**	423.720819	330.68879	y5	15.5
YwbN	GEYIAQR	418.716684	779.404629	y6	15.5
YwbN	GEYIAQR	418.716684	650.362036	y5	15.5
YwbN	GEYIAQR	418.716684	487.298707	y4	15.5
YwbN	GEYIAQR	418.716684	325.684656	y5	15.5

Supplementary Table S2. Quantified peptides of QcrA and YwbN proteins are listed providing their parent mass (Q1 mass) and fragment masses (Q3 mass). Corresponding collision energy (CE) is also provided. Amino acids containing ^{13}C and ^{15}N are printed in bolt. Transitions highlighted in red were not used for further quantification or analysis purposes.

Supplementary Table S3. Cell dimensions and cell surface area of midi*Bacillus*.

	Induced			Non Induced		
Cell number	Width (µm)	Length (µm)	Surface area (µm2)	Width (µm)	Length (µm)	Surface area (µm2)
1	0.81	10.10	27.82	0.66	9.29	20.58
2	1.10	9.18	35.58	0.61	14.37	28.52
3	1.02	11.58	40.27	0.71	6.66	16.48
4	0.80	6.61	18.65	0.76	7.22	19.02
5	0.69	9.40	21.89	0.96	12.17	39.56
6	0.96	7.46	25.53	0.94	8.24	27.21
7	0.70	8.49	20.08	0.64	5.08	11.55
8	0.92	9.19	29.11	0.59	5.47	11.24
9	0.85	6.71	20.29	0.47	7.20	11.44
10	0.89	9.02	27.74	0.85	11.09	32.05
11	0.97	10.60	35.26	0.75	15.07	37.19
12	0.94	7.27	24.35	0.71	9.54	22.92
13	0.66	11.98	26.16	0.96	10.93	36.04
14	0.88	8.72	26.54	0.56	11.99	22.24
15	0.86	14.20	40.92	0.94	14.86	46.57
16	0.95	9.58	31.57	0.87	6.44	20.11
17	0.66	7.17	16.20	0.93	9.01	28.97
18	0.65	10.80	23.32	1.02	6.25	23.22
19	0.71	5.80	14.55	0.65	9.10	19.85
20	0.78	11.18	29.32	0.58	6.58	13.04
21	0.59	7.85	15.66	0.84	22.24	61.15
22	0.94	8.46	27.88	0.73	5.64	14.66
23	0.58	8.41	16.38	1.18	7.86	33.52
24	1.34	13.50	62.39	1.14	7.80	32.13
25	0.82	12.83	35.02	0.79	10.81	28.60
26	0.83	7.84	22.52	0.81	5.54	16.19
27	0.96	8.75	29.45	1.00	15.49	51.61
28	0.86	10.41	30.61	1.05	12.02	43.05
29	1.08	7.73	29.73	1.37	13.91	65.50
30	0.91	6.12	20.14	0.82	4.24	13.08
31	1.48	18.80	94.00	0.92	5.47	18.40
32	1.28	14.43	63.21	1.05	14.25	50.67
33	1.18	11.29	46.02	1.13	16.48	62.70
34	1.19	17.69	70.31	1.04	12.68	44.99
35	1.28	16.92	72.90	0.87	5.45	17.28
36	1.33	12.44	57.69	0.95	9.53	31.24
37	1.09	9.72	37.07	1.15	6.07	26.20
38	1.05	13.32	47.36	0.86	8.75	25.93
39	0.97	11.83	38.99	0.96	10.35	34.08
40	1.14	9.26	37.19	0.80	6.38	18.08

	Induced			Non Induced		
Cell number	Width (µm)	Length (µm)	Surface area (µm2)	Width (µm)	Length (µm)	Surface area (µm2)
41	1.41	15.33	74.28	1.19	16.96	67.92
42	1.15	9.19	37.50	1.28	14.78	64.61
43	1.10	11.56	43.83	1.37	13.27	62.76
44	1.28	11.34	50.76	1.19	12.95	52.68
45	0.98	12.76	42.09	1.16	15.75	61.91
46	1.31	13.42	60.47	1.00	4.24	16.50
47	1.07	15.70	56.35	1.08	12.72	46.85
48	0.90	9.10	28.32	1.25	11.33	49.57
49	1.09	13.44	49.80	1.31	25.24	109.01
50	1.15	9.69	39.31	1.04	12.59	44.44
51	1.25	13.95	59.92	1.09	7.94	30.96
52	1.38	16.80	78.85	1.04	14.75	51.78
53	0.91	7.87	25.01	1.26	13.96	60.23
54	1.12	14.27	54.00	0.91	13.46	41.15
55	1.00	11.59	39.40	1.05	13.27	47.16
56	1.23	5.26	25.16	1.28	15.25	66.20
57	1.04	8.73	31.85	0.87	7.09	21.76
58	1.05	11.06	39.89	1.10	11.37	42.94
59	1.17	15.34	60.69	0.76	8.15	21.40
60	1.52	14.89	78.26	1.21	20.11	80.81
61	0.93	11.35	35.77	0.57	9.87	18.85
62	1.13	17.72	67.11	0.79	13.80	36.23
63	1.34	16.73	76.32	0.77	5.80	15.89
64	1.61	12.72	72.63	0.82	6.47	18.71
65	0.90	15.02	44.80	0.65	5.40	12.32
66	1.61	19.64	107.66	1.29	9.75	44.79
67	1.59	12.35	69.71	0.92	14.57	44.90
68	1.31	25.58	110.88	0.88	10.59	31.71
69	1.35	12.97	60.93	1.01	18.12	60.50
70	0.67	16.13	35.61	0.86	9.00	26.79
71	1.45	10.62	55.18	0.92	14.71	45.31
72	1.01	12.00	41.12	0.75	10.94	27.47
73	1.39	13.04	63.08	0.98	15.63	50.87
74	0.88	11.67	34.70	1.59	11.24	64.14
75	0.70	13.25	30.72	0.94	18.16	56.61
76	0.87	7.29	22.45	1.02	14.51	49.63
77	0.99	23.44	75.64	1.41	17.49	83.89
78	0.73	8.32	20.66	1.03	10.26	36.43
79	1.52	18.56	96.08	1.29	17.57	76.16
80	1.39	13.25	63.76	1.24	14.71	62.34
81	0.90	16.04	47.68	1.10	12.33	46.23

	Induced			Non Induced		
Cell number	Width (μm)	Length (μm)	Surface area (μm2)	Width (μm)	Length (μm)	Surface area (μm2)
82	1.07	13.10	47.62	1.08	14.08	51.18
83	1.13	14.87	56.98	1.21	10.50	44.40
84	1.24	16.65	69.62	1.03	7.99	29.28
85	0.91	14.55	44.01	1.68	12.56	74.98
86	1.48	18.87	94.68	1.49	17.13	87.25
87	1.59	20.04	108.15	1.28	14.33	62.81
88	1.20	12.80	52.84	0.89	7.57	23.53
89	1.38	10.38	50.79	0.62	5.63	12.11
90	1.04	13.85	48.56	1.57	9.97	56.92
91	1.23	20.10	82.64	1.04	13.93	48.82
92	1.99	18.50	128.25	0.83	14.14	39.16
93	1.67	6.49	42.69	0.97	20.53	65.49
94	1.59	17.90	97.48	0.95	6.24	21.42
95	1.86	11.71	79.06	1.45	12.57	64.09
96	1.19	10.68	44.41	0.96	5.95	20.95
97	1.69	20.36	117.23	1.89	15.77	104.98
98	1.35	15.26	70.41	0.83	12.45	34.74
99	1.37	11.88	56.80	1.88	21.77	139.81
100	1.49	16.72	85.35	1.22	11.43	48.59
101	0.67	5.89	13.91	1.61	11.37	65.53
102	1.18	10.55	43.30	1.01	5.79	21.48
103	0.90	10.48	32.01	0.70	8.82	20.97
104	0.86	12.33	35.60	0.80	13.44	35.59
105	1.45	10.37	53.80	1.08	12.94	47.35
106	0.98	16.55	54.00	1.10	16.30	59.90
107	1.01	14.55	49.19	0.95	8.49	28.12
108	1.26	11.49	50.47	1.06	17.91	63.13
109	1.19	9.89	41.47	1.19	19.45	77.23
110	0.78	4.95	14.04	1.35	13.04	61.01
111	1.41	15.58	75.42	1.38	7.77	39.69
112	0.85	13.07	37.35	1.33	19.17	85.88
113	1.12	9.52	37.55	1.23	24.80	100.85
114	0.72	15.83	37.54	1.09	10.44	39.52
115	1.36	11.46	54.78	1.26	14.01	60.69
116	1.45	14.31	72.04	0.84	8.35	24.35
117	1.28	9.82	44.66	1.18	8.00	33.86
118	0.64	4.56	10.42	0.72	18.43	43.44
119	1.27	13.22	57.81	0.86	19.20	54.48
120	0.98	6.30	22.28	1.28	19.12	81.73
121	0.71	8.71	20.88	0.67	12.30	27.50
122	0.90	11.27	34.25	0.90	7.13	22.58

	Induced			Non Induced		
Cell number	Width (µm)	Length (µm)	Surface area (µm2)	Width (µm)	Length (µm)	Surface area (µm2)
123	0.89	17.75	51.82	0.77	10.96	28.55
124	1.14	13.64	52.85	0.84	14.44	40.21
125	0.86	9.86	28.92	0.92	10.85	34.10
126	1.05	12.16	43.52	0.95	7.28	24.53
127	0.85	21.95	60.78	0.99	16.99	55.98
128	0.74	13.83	33.78	1.25	13.33	57.20
129	1.01	19.94	66.25	1.05	11.83	42.67
130	0.86	6.36	19.47	1.09	13.30	49.08
131	0.74	15.52	37.69	0.72	10.01	24.16
132	0.86	17.50	49.86	0.96	13.89	45.02
133	0.99	14.49	47.93	1.03	17.90	61.11
134	0.82	6.54	18.87	1.12	11.35	43.98
135	0.74	6.20	16.09	0.73	11.77	28.55
136	0.93	9.95	31.70	0.73	16.48	39.32
137	0.75	9.68	24.52	1.15	13.43	52.90
138	1.03	8.52	31.01	0.98	16.45	53.69
139	1.05	15.75	55.66	1.13	16.61	62.86
140	0.84	12.05	34.15	1.13	20.44	76.79
141	0.80	9.90	26.93	0.70	11.32	26.46
142	0.85	12.52	35.88	0.79	13.46	35.15
143	0.91	10.08	31.29	1.15	13.45	52.96
144	0.94	17.56	54.84	0.76	8.50	22.07
145	0.94	13.29	41.92	0.73	15.88	38.23
146	1.06	11.25	41.18	0.81	6.11	17.52
147	0.72	11.40	27.28	1.13	8.86	35.41
148	1.06	14.23	50.88	0.87	14.02	40.69
149	0.89	9.56	29.24	1.25	16.05	68.18
150	0.86	12.47	35.99	0.76	7.79	20.39
151	0.77	14.84	37.72	0.73	13.50	32.75
152	0.78	16.17	41.53	0.97	10.15	33.86
153	1.10	15.49	57.14	1.23	12.42	52.91
154	1.18	10.09	41.81	0.79	10.52	27.90
155	0.74	10.74	26.81	0.77	18.63	47.22
156	0.84	10.25	29.38	0.73	18.40	44.03
157	1.09	9.31	35.45	0.85	12.13	34.82
158	1.01	13.78	47.01	0.80	11.20	30.22
159	1.18	11.99	48.61	0.71	12.70	29.74
160	0.82	9.21	25.74	0.82	12.39	33.88
161	1.10	8.92	34.49	0.75	8.75	22.33
162	0.94	9.64	31.19	0.91	11.85	36.34
163	0.68	11.62	26.26	1.43	16.33	79.70

	Induced			Non Induced		
Cell number	Width (µm)	Length (µm)	Surface area (µm2)	Width (µm)	Length (µm)	Surface area (µm2)
164	1.12	10.98	42.48	1.36	11.34	54.26
165	0.86	8.71	25.82	0.86	10.91	31.76
166	0.90	13.53	40.62	1.41	8.62	44.34
167	1.09	14.55	53.32	0.76	24.79	60.92
168	1.06	11.26	40.99	1.41	18.17	86.88
169	0.90	10.85	33.07	1.07	17.95	63.91
170	0.72	12.93	30.73	1.45	11.51	58.99
171	0.87	21.72	62.11	1.00	7.18	25.60
172	0.86	20.16	57.09	0.93	8.01	26.20
173	1.00	20.30	66.63	1.46	11.68	60.28
174	0.76	12.24	31.22	1.20	13.52	55.33
175	0.87	19.42	55.43	1.28	9.37	42.66
176	0.92	16.64	50.90	1.45	10.93	56.60
177	1.08	6.68	26.19	0.88	14.73	43.17
178	0.94	7.98	26.44	1.00	20.09	66.35
179	0.75	12.90	32.09	1.12	7.71	31.15
180	0.97	7.35	25.35	0.97	18.02	57.87
Median	0.99	11.85	40.95	0.97	12.07	40.92
SD	0.27	3.97	21.71	0.26	4.40	21.29

Supplementary Table S3. One hundred and eighty cells of midi*Bacillus* were measured with respect to their cellular dimensions and surface area, assuming rod-shaped *Bacillus* cells to form a cylinder and two half spheres. Median and standard deviation (SD) for the 180 measurements are listed.

Supplementary Table S4. Sequences of MS-quantified AQUA peptides of midi*Bacillus* used in SRM experiment

Protein	Accession number	Peptide sequence	Used for quantification
QcrA	BSU22560	FALDPVLK	×
QcrA	BSU22560	INQVDAWYESEESR	×
QcrA	BSU22560	DGTNVPGTPPLAPLDHYEQEVK	
QcrA	BSU22560	DGFLYLGK	×
YwbN	BSU38260	GFLSGGK	
YwbN	BSU38260	DQEDTFGR	×
YwbN	BSU38260	LNQIPSNSHVSLAK	×
YwbN	BSU38260	GEYIAQR	×

Supplementary Table S4. Quantified AQUA peptides of the two *B. subtilis* proteins used for SRM approach are listed. Peptides used for quantification are indicated in column “used for quantification”.

Supplementary Table S5. Absolute protein amount for membrane proteins of midi*Bacillus* quantified by shotgun MS and calibrated via targeted MS.

			Control					1% subtilin				
Accession number	Locus tag	Mol. Weight [kDa]	ng/µg	fg/µm²	molecules/µm²	molecules/cell	RSD (%)	ng/µg	fg/µm²	molecules/µm²	molecules/cell	RSD (%)
BSU08070	acoB	36.845	0.061	2.09E-06	0.034	1.479	14.71	0.067	3.84E-06	0.063	2.838	17.88
BSU29680	acsA	64.891	0.011	3.57E-07	0.003	0.144	6.73	0.032	1.86E-06	0.017	0.778	17.15
BSU40100	ahpF	54.874	0.011	3.75E-07	0.004	0.178	38.23	0.037	2.13E-06	0.023	1.058	25.59
BSU18120	alsT	50.299	0.226	7.67E-06	0.092	3.982	11.87	0.340	1.95E-05	0.233	10.537	29.24
BSU11390	appB	35.278	0.016	5.40E-07	0.009	0.400	34.85	0.001	7.08E-08	0.001	0.055	11.56
BSU11400	appC	33.419	0.010	3.47E-07	0.006	0.271	29.98	0.005	3.05E-07	0.005	0.248	49.75
BSU11360	appD	36.311	0.002	7.65E-08	0.001	0.055	8.31	0.001	5.12E-08	0.001	0.038	47.17
BSU11370	appF	37.086	0.013	4.46E-07	0.007	0.314	19.94	0.003	1.68E-07	0.003	0.123	49.66
BSU23970	artQ	23.897	0.269	9.13E-06	0.230	9.971	21.01	0.250	1.43E-05	0.361	16.330	21.52
BSU23960	artR	26.949	0.195	6.63E-06	0.148	6.423	26.11	0.170	9.77E-06	0.218	9.861	22.14
BSU05340	aseA	47.321	0.003	9.39E-08	0.001	0.052	42.65	0.003	1.58E-07	0.002	0.091	23.76
BSU36870	atpB	27.054	0.188	6.37E-06	0.142	6.149	19.01	0.300	1.72E-05	0.382	17.280	7.13
BSU36810	atpD	51.42	0.374	1.27E-05	0.149	6.445	25.72	0.401	2.30E-05	0.269	12.160	9.82
BSU36860	atpE	7.0935	0.293	9.94E-06	0.844	36.582	28.87	0.354	2.03E-05	1.726	77.983	9.60
BSU36850	atpF	19.208	1.500	5.10E-05	1.598	69.273	10.90	2.160	1.24E-04	3.885	175.511	4.60
BSU09460	bcaP	49.71	0.007	2.24E-07	0.003	0.118	16.70	0.010	5.64E-07	0.007	0.309	20.26
BSU33470	bdbC	15.714	0.009	3.06E-07	0.012	0.509	26.62	0.016	8.94E-07	0.034	1.547	11.15
BSU33480	bdbD	24.905	0.500	1.70E-05	0.411	17.806	4.96	0.696	3.99E-05	0.966	43.625	5.64
BSU34820	bmrA	64.518	0.132	4.48E-06	0.042	1.813	7.80	0.188	1.08E-05	0.100	4.538	9.14
BSU09710	bmrC	65.111	0.094	3.20E-06	0.030	1.282	63.38	0.027	1.55E-06	0.014	0.647	1.41
BSU09720	bmrD	76.304	0.058	1.96E-06	0.015	0.671	11.46	0.021	1.21E-06	0.010	0.430	13.89
BSU29600	braB	46.608	0.018	6.23E-07	0.008	0.349	18.96	0.045	2.61E-06	0.034	1.523	21.56
BSU33490	cadA	75.036	0.111	3.77E-06	0.030	1.312	16.48	0.165	9.45E-06	0.076	3.425	18.04
BSU25190	cccA	12.766	0.055	1.86E-06	0.088	3.801	30.45	0.046	2.65E-06	0.125	5.655	19.23
BSU35270	cccB	11.928	0.090	3.06E-06	0.154	6.696	26.81	0.116	6.66E-06	0.336	15.201	18.47
BSU01750	cdaA	30.546	0.036	1.22E-06	0.024	1.043	5.98	0.067	3.82E-06	0.075	3.399	18.42

Accession number	Locus tag	Mol. Weight [kDa]	Control					1% subtilin				
			ng/µg	fg/µm²	molecules/µm²	molecules/cell	RSD (%)	ng/µg	fg/µm²	molecules/µm²	molecules/cell	RSD (%)
BSU16540	cdsA	29.866	0.015	5.21E-07	0.011	0.456	19.05	0.020	1.16E-06	0.023	1.058	32.07
BSU38770	cimH	48.339	0.085	2.88E-06	0.036	1.558	22.64	0.077	4.41E-06	0.055	2.484	23.66
BSU16930	cinA	45.537	0.004	1.22E-07	0.002	0.070	19.15	0.019	1.09E-06	0.014	0.654	20.98
BSU07610	citM	45.83	0.466	1.58E-05	0.208	9.025	4.16	0.332	1.90E-05	0.250	11.295	10.56
BSU07580	citS	59.89	0.001	3.30E-08	0.000	0.014	61.23	0.002	1.26E-07	0.001	0.057	14.97
BSU36590	clsA	55.865	0.017	5.68E-07	0.006	0.266	12.95	0.030	1.70E-06	0.018	0.828	8.67
BSU23760	coaA	36.639	0.002	6.45E-08	0.001	0.046	32.25	0.004	2.48E-07	0.004	0.184	30.53
BSU25590	comEA	21.769	0.120	4.08E-06	0.113	4.892	36.30	0.298	1.71E-05	0.473	21.384	40.78
BSU25570	comEC	86.7	0.001	4.30E-08	0.000	0.013	42.77	0.006	3.30E-07	0.002	0.104	55.84
BSU24730	comGA	40.458	0.070	2.37E-06	0.035	1.527	35.60	0.199	1.14E-05	0.170	7.660	63.94
BSU24720	comGB	39.762	0.043	1.47E-06	0.022	0.965	24.44	0.122	6.97E-06	0.106	4.769	67.99
BSU24710	comGC	10.85	0.057	1.94E-06	0.108	4.679	25.10	0.096	5.50E-06	0.305	13.799	39.55
BSU24700	comGD	16.008	0.003	1.17E-07	0.004	0.191	35.95	0.012	6.83E-07	0.026	1.160	62.26
BSU24690	comGE	13.271	0.004	1.27E-07	0.006	0.250	13.29	0.015	8.60E-07	0.039	1.763	36.41
BSU24680	comGF	14.281	0.005	1.56E-07	0.007	0.285	41.93	0.017	1.00E-06	0.042	1.909	35.53
BSU24670	comGG	14.54	0.010	3.36E-07	0.014	0.603	4.13	0.033	1.87E-06	0.077	3.492	53.04
BSU33500	copA	85.911	0.081	2.74E-06	0.019	0.833	41.98	0.050	2.84E-06	0.020	0.901	4.24
BSU39810	csbC	50.14	0.002	5.26E-08	0.001	0.027	30.12	0.003	1.90E-07	0.002	0.103	53.02
BSU33020	cssS	52.097	0.027	9.14E-07	0.011	0.458	3.59	0.040	2.32E-06	0.027	1.210	5.91
BSU14870	ctaA	34.084	0.040	1.34E-06	0.024	1.029	58.90	0.057	3.29E-06	0.058	2.629	28.04
BSU14880	ctaB	33.945	0.018	6.14E-07	0.011	0.472	28.57	0.100	5.76E-06	0.102	4.617	8.52
BSU14890	ctaC	40.094	0.212	7.20E-06	0.108	4.689	2.64	0.057	3.26E-06	0.049	2.210	19.28
BSU14900	ctaD	69.027	0.203	6.89E-06	0.060	2.604	25.01	0.052	2.97E-06	0.026	1.172	11.15
BSU14910	ctaE	23.266	0.094	3.18E-06	0.082	3.567	27.64	0.032	1.82E-06	0.047	2.124	7.42
BSU14920	ctaF	12.62	0.032	1.09E-06	0.052	2.263	33.73	0.007	3.93E-07	0.019	0.847	2.02
BSU14930	ctaG	34.069	0.012	4.21E-07	0.007	0.323	29.88	0.004	2.08E-07	0.004	0.166	9.67
BSU38760	cydA	52.292	0.062	2.10E-06	0.024	1.047	16.48	0.013	7.48E-07	0.009	0.389	25.42
BSU38750	cydB	37.861	0.071	2.40E-06	0.038	1.655	25.89	0.016	9.14E-07	0.015	0.657	6.43

			Control					1% subtilin				
Accession number	Locus tag	Mol. Weight [kDa]	ng/µg	fg/µm²	molecules/µm²	molecules/cell	RSD (%)	ng/µg	fg/µm²	molecules/µm²	molecules/cell	RSD (%)
BSU38740	cydC	62.805	0.038	1.29E-06	0.012	0.534	5.80	0.006	3.69E-07	0.004	0.160	29.44
BSU38730	cydD	64.509	0.029	9.87E-07	0.009	0.400	24.00	0.009	4.93E-07	0.005	0.208	15.07
BSU00100	dacA	48.636	0.834	2.84E-05	0.351	15.217	11.21	1.384	7.94E-05	0.983	44.409	20.31
BSU00290	darA	11.967	0.001	3.21E-08	0.002	0.070	**45.05**	0.003	1.93E-07	0.010	0.438	27.66
BSU04470	dctP	45.441	0.131	4.44E-06	0.059	2.548	13.62	0.282	1.62E-05	0.215	9.697	7.95
BSU04450	dctS	59.941	0.001	5.01E-08	0.001	0.022	12.12	0.001	5.62E-08	0.001	0.026	**53.93**
BSU35500	degS	44.957	0.049	1.65E-06	0.022	0.957	20.31	0.070	4.01E-06	0.054	2.424	14.93
BSU19180	des	40.708						0.002	8.89E-08	0.001	0.059	**36.32**
BSU19190	desK	42.672	0.001	5.07E-08	0.001	0.031	23.73	0.001	8.13E-08	0.001	0.052	6.29
BSU09120	dgcK	40.692	0.005	1.58E-07	0.002	0.101	13.48	0.005	3.10E-07	0.005	0.207	23.86
BSU15240	divIB	30.166	0.038	1.30E-06	0.026	1.122	4.81	0.071	4.09E-06	0.082	3.692	11.67
BSU00620	divIC	14.721	0.004	1.38E-07	0.006	0.245	**35.27**	0.009	5.16E-07	0.021	0.953	**36.22**
BSU38510	dltB	46.636	0.015	5.17E-07	0.007	0.290	16.64	0.044	2.51E-06	0.032	1.464	10.09
BSU12920	dppA	30.158	0.001	3.51E-08	0.001	0.030	25.44	0.001	6.64E-08	0.001	0.060	10.81
BSU03670	dtpT	53.249	0.036	1.21E-06	0.014	0.593	11.64	0.063	3.62E-06	0.041	1.852	17.26
BSU22030	dynA	137.38	0.048	1.63E-06	0.007	0.309	6.51	0.108	6.19E-06	0.027	1.225	13.46
BSU10040	ecsA	27.722	0.070	2.37E-06	0.052	2.234	15.19	0.117	6.73E-06	0.146	6.607	13.89
BSU10050	ecsB	47.299	0.053	1.79E-06	0.023	0.989	10.43	0.080	4.60E-06	0.059	2.646	8.55
BSU38280	efeU	52.387	0.019	6.31E-07	0.007	0.315	2.23	0.049	2.79E-06	0.032	1.450	21.73
BSU25290	era	34.074	0.017	5.86E-07	0.010	0.449	14.10	0.046	2.61E-06	0.046	2.086	22.03
BSU29610	ezrA	64.995	0.265	8.99E-06	0.083	3.610	5.49	0.458	2.63E-05	0.244	11.001	23.39
BSU11340	fabF	44.004	0.002	6.97E-08	0.001	0.041	29.16	0.006	3.34E-07	0.005	0.207	21.41
BSU11720	fabI	27.873	0.008	2.79E-07	0.006	0.261	4.63	0.041	2.38E-06	0.051	2.319	4.67
BSU37180	fadF	79.186	0.093	3.17E-06	0.024	1.045	13.33	0.003	1.95E-07	0.001	0.067	25.40
BSU07520	fecC	35.02	0.207	7.03E-06	0.121	5.243	6.26	0.258	1.48E-05	0.254	11.479	11.72
BSU07500	fecE	35.218	0.004	1.24E-07	0.002	0.092	22.91	0.006	3.32E-07	0.006	0.257	23.21
BSU07490	fecF	29.458	0.004	1.44E-07	0.003	0.128	12.91	0.008	4.63E-07	0.009	0.428	30.79
BSU01630	feuA	35.108	0.046	1.56E-06	0.027	1.157	10.22	0.036	2.09E-06	0.036	1.621	10.49

			Control					1% subtilin				
Accession number	Locus tag	Mol. Weight [kDa]	ng/µg	fg/µm²	molecules/µm²	molecules/cell	RSD (%)	ng/µg	fg/µm²	molecules/µm²	molecules/cell	RSD (%)
BSU15980	ffh	49.54	0.087	2.96E-06	0.036	1.560	16.83	0.166	9.50E-06	0.116	5.219	16.87
BSU33310	fhuB	36.462	0.003	1.04E-07	0.002	0.075	27.27	0.004	2.31E-07	0.004	0.172	25.83
BSU33290	fhuC	29.954	0.008	2.67E-07	0.005	0.233	26.59	0.013	7.21E-07	0.014	0.655	5.54
BSU33320	fhuD	34.426	0.189	6.41E-06	0.112	4.863	7.43	0.135	7.73E-06	0.135	6.113	17.64
BSU33300	fhuG	36.154	0.005	1.73E-07	0.003	0.125	65.63	0.006	3.49E-07	0.006	0.263	39.86
BSU31010	floT	55.993	0.575	1.96E-05	0.210	9.115	24.99	0.431	2.47E-05	0.266	12.023	11.01
BSU09600	fluC	12.448	0.001	4.88E-08	0.002	0.102	28.33	0.002	1.38E-07	0.007	0.301	35.50
BSU14400	fruA	67.183	0.306	1.04E-05	0.093	4.041	6.17	0.543	3.12E-05	0.279	12.615	11.48
BSU35260	ftsE	25.619	0.044	1.48E-06	0.035	1.510	18.39	0.086	4.91E-06	0.115	5.217	7.39
BSU00690	ftsH	70.936	2.647	8.99E-05	0.763	33.094	7.98	2.862	1.64E-04	1.394	62.978	9.94
BSU15150	ftsL	13.073	0.001	4.29E-08	0.002	0.086	26.78	0.001	6.48E-08	0.003	0.135	30.75
BSU14850	ftsW	43.731	0.015	5.09E-07	0.007	0.304	23.28	0.030	1.73E-06	0.024	1.075	20.07
BSU35250	ftsX	33.099	0.336	1.14E-05	0.208	8.999	4.69	0.595	3.41E-05	0.621	28.050	13.34
BSU15950	ftsY	36.342	0.091	3.10E-06	0.051	2.226	23.31	0.127	7.26E-06	0.120	5.438	15.06
BSU02350	gamP	68.144	0.322	1.09E-05	0.097	4.194	6.08	0.427	2.45E-05	0.217	9.786	21.14
BSU35690	ggaA	53.148	0.000	1.30E-08	0.000	0.006	45.64	0.028	1.63E-06	0.018	0.835	18.33
BSU02440	glnK	51.599	0.007	2.34E-07	0.003	0.119	10.23	0.004	2.26E-07	0.003	0.119	34.93
BSU09280	glpF	28.735	0.120	4.09E-06	0.086	3.718	28.99	0.204	1.17E-05	0.245	11.083	31.70
BSU02140	glpT	49.801	0.158	5.38E-06	0.065	2.821	52.42	0.318	1.82E-05	0.220	9.954	13.73
BSU18450	gltA	168.77	0.006	2.04E-07	0.001	0.032	27.14	0.042	2.44E-06	0.009	0.393	9.71
BSU02340	gltP	44.614	0.016	5.41E-07	0.007	0.316	21.90	0.011	6.08E-07	0.008	0.371	38.07
BSU10220	gltT	45.919	0.987	3.35E-05	0.440	19.059	2.86	0.820	4.70E-05	0.617	27.861	10.13
BSU40070	gntP	46.655	0.020	6.88E-07	0.009	0.385	13.89	0.033	1.89E-06	0.024	1.103	34.52
BSU38210	gtcA	14.349	0.001	1.81E-08	0.001	0.033	30.39	0.001	3.36E-08	0.001	0.064	52.62
BSU10140	hemY	51.202	0.035	1.20E-06	0.014	0.610	17.39	0.036	2.07E-06	0.024	1.102	8.87
BSU13490	htpX	32.865	0.184	6.24E-06	0.114	4.960	26.20	0.115	6.60E-06	0.121	5.460	3.60
BSU33000	htrB	48.717	0.004	1.39E-07	0.002	0.074	9.01	0.015	8.41E-07	0.010	0.470	46.74
BSU39390	hutM	51.624	0.008	2.78E-07	0.003	0.141	18.77					

			Control					1% subtilin				
Accession number	Locus tag	Mol. Weight [kDa]	ng/µg	fg/µm²	molecules/µm²	molecules/cell	RSD (%)	ng/µg	fg/µm²	molecules/µm²	molecules/cell	RSD (%)
BSU31460	kapB	14.668						0.000	1.67E-08	0.001	0.031	37.47
BSU04320	kimA	66.771	0.054	1.84E-06	0.017	0.719	11.04	0.101	5.78E-06	0.052	2.357	17.02
BSU13990	kinA	69.171	0.001	3.87E-08	0.000	0.015	26.31	0.001	4.97E-08	0.000	0.020	64.66
BSU31450	kinB	47.812	0.006	2.03E-07	0.003	0.111	11.90	0.007	3.76E-07	0.005	0.214	1.26
BSU14490	kinC	48.846	0.021	7.10E-07	0.009	0.380	4.07	0.028	1.63E-06	0.020	0.906	20.98
BSU13530	kinE	85.512	0.001	2.41E-08	0.000	0.007	78.44	0.002	1.38E-07	0.001	0.044	45.07
BSU03060	lctP	57.603	0.037	1.25E-06	0.013	0.568	21.80	0.010	5.56E-07	0.006	0.263	13.25
BSU25510	lepA	68.586	0.069	2.33E-06	0.020	0.886	13.24	0.156	8.96E-06	0.079	3.554	10.47
BSU34990	lgt	30.619	0.251	8.54E-06	0.168	7.279	14.36	0.478	2.74E-05	0.539	24.362	20.68
BSU33100	liaF	27.024	0.005	1.77E-07	0.004	0.171	72.84	0.007	3.94E-07	0.009	0.396	46.18
BSU33120	liaH	25.698						0.012	7.12E-07	0.017	0.754	67.49
BSU33090	liaS	40.704	0.004	1.49E-07	0.002	0.095	22.33	0.007	4.10E-07	0.006	0.274	29.10
BSU38580	licC	48.533	0.216	7.35E-06	0.091	3.954	17.57	0.305	1.75E-05	0.217	9.809	15.25
BSU07710	ltaS	74.233	0.008	2.69E-07	0.002	0.095	54.78	0.088	5.03E-06	0.041	1.845	11.48
BSU35640	lytA	11.228	0.026	8.79E-07	0.047	2.045	41.18	0.020	1.15E-06	0.062	2.786	60.48
BSU28930	lytS	64.854	0.006	2.18E-07	0.002	0.088	7.53	0.013	7.19E-07	0.007	0.302	13.97
BSU31580	maeN	47.824	0.002	7.86E-08	0.001	0.043	4.36	0.008	4.59E-07	0.006	0.261	25.90
BSU28050	maf	21.295	0.035	1.18E-06	0.033	1.442	0.77	0.083	4.75E-06	0.134	6.068	33.74
BSU31520	malK	58.929	0.013	4.38E-07	0.004	0.194	13.23	0.023	1.30E-06	0.013	0.599	18.13
BSU12000	manR	75.35	0.028	9.46E-07	0.008	0.328	18.66	0.099	5.70E-06	0.046	2.059	22.57
BSU13950	mcpC	72.03	0.054	1.84E-06	0.015	0.668	37.44	0.057	3.28E-06	0.027	1.238	35.27
BSU03070	mdr	55.689	0.037	1.27E-06	0.014	0.596	14.26	0.068	3.90E-06	0.042	1.905	2.69
BSU32880	mdtP	57.686	0.082	2.79E-06	0.029	1.264	34.13	0.193	1.11E-05	0.115	5.213	24.58
BSU38490	menA	33.838	0.027	9.04E-07	0.016	0.698	13.19	0.043	2.49E-06	0.044	2.000	25.59
BSU32750	metN	37.885	0.114	3.86E-06	0.061	2.658	8.46	0.240	1.38E-05	0.219	9.878	23.78
BSU32740	metP	23.756	0.025	8.41E-07	0.021	0.924	43.44	0.060	3.43E-06	0.087	3.923	5.92
BSU32730	metQ	30.355	0.674	2.29E-05	0.454	19.690	1.21	1.183	6.78E-05	1.346	60.798	15.39
BSU13300	mgtE	50.844	0.097	3.29E-06	0.039	1.688	12.83	0.083	4.78E-06	0.057	2.556	13.32

			Control					1% subtilin				
Accession number	Locus tag	Mol. Weight [kDa]	ng/µg	fg/µm²	molecules/µm²	molecules/cell	RSD (%)	ng/µg	fg/µm²	molecules/µm²	molecules/cell	RSD (%)
BSU05500	mhqP	13.276	0.001	3.53E-08	0.002	0.070	59.05	0.008	4.47E-07	0.020	0.916	26.12
BSU27990	minD	29.407	0.075	2.54E-06	0.052	2.257	7.60	0.136	7.83E-06	0.160	7.240	22.16
BSU35220	minJ	43.666	0.075	2.56E-06	0.035	1.532	11.69	0.112	6.42E-06	0.089	4.000	9.93
BSU23560	mleN	50.211	1.215	4.13E-05	0.495	21.458	11.55	1.775	1.02E-04	1.221	55.170	16.07
BSU30770	mntA	33.418	0.215	7.30E-06	0.132	5.700	12.28	0.045	2.56E-06	0.046	2.083	17.01
BSU30760	mntB	27.881	0.059	2.02E-06	0.044	1.891	32.72	0.011	6.38E-07	0.014	0.623	10.23
BSU30750	mntC	47.944	0.034	1.15E-06	0.015	0.629	5.99	0.006	3.36E-07	0.004	0.191	10.88
BSU04360	mntH	45.685	0.047	1.60E-06	0.021	0.912	4.32	0.028	1.62E-06	0.021	0.962	8.07
BSU29730	motP	30.143	0.003	1.14E-07	0.002	0.099	41.28	0.011	6.36E-07	0.013	0.574	36.37
BSU29720	motS	27.595	0.003	1.11E-07	0.002	0.105	24.81	0.007	4.00E-07	0.009	0.394	15.06
BSU08425	mprF	96.321	0.028	9.53E-07	0.006	0.258	18.98	0.049	2.80E-06	0.018	0.792	10.30
BSU28010	mreD	19.816	0.004	1.26E-07	0.004	0.165	20.13	0.009	5.02E-07	0.015	0.689	22.55
BSU31600	mrpA	89.53	0.032	1.09E-06	0.007	0.317	31.67	0.042	2.41E-06	0.016	0.732	21.03
BSU31620	mrpC	12.285	0.003	9.70E-08	0.005	0.206	31.72	0.006	3.20E-07	0.016	0.709	9.65
BSU31630	mrpD	53.476	0.014	4.78E-07	0.005	0.233	9.61	0.022	1.28E-06	0.014	0.653	50.97
BSU31640	mrpE	18.38	0.007	2.49E-07	0.008	0.353	12.35	0.005	3.14E-07	0.010	0.465	97.03
BSU31650	mrpF	10.244	0.002	6.62E-08	0.004	0.169	28.59	0.003	1.72E-07	0.010	0.456	6.51
BSU31660	mrpG	13.626	0.014	4.90E-07	0.022	0.940	12.32	0.016	9.02E-07	0.040	1.801	12.19
BSU36360	mscL	17.136	0.028	9.65E-07	0.034	1.470	7.20	0.063	3.60E-06	0.127	5.718	16.09
BSU30270	msmE	48.237	0.016	5.33E-07	0.007	0.288	10.16	0.007	3.79E-07	0.005	0.214	4.56
BSU03980	mtlA	50.184	0.105	3.56E-06	0.043	1.851	27.87	0.238	1.37E-05	0.164	7.406	12.64
BSU07340	mtrA	48.905	0.008	2.58E-07	0.003	0.138	12.57	0.018	1.03E-06	0.013	0.575	18.70
BSU15220	murG	39.936	0.044	1.51E-06	0.023	0.986	21.44	0.096	5.52E-06	0.083	3.763	2.82
BSU30050	murJ	59.471	0.010	3.38E-07	0.003	0.148	25.39	0.018	1.04E-06	0.011	0.476	37.81
BSU01680	murP	47.6	0.003	9.66E-08	0.001	0.053	22.45	0.002	8.93E-08	0.001	0.051	30.84
BSU07700	nagP	48.58	1.047	3.56E-05	0.441	19.110	9.94	1.407	8.07E-05	1.000	45.196	9.72
BSU37280	narG	139.1	0.015	5.15E-07	0.002	0.097	13.64					
BSU37270	narH	55.472	0.003	9.14E-08	0.001	0.043	81.91					

			Control					1% subtilin				
Accession number	Locus tag	Mol. Weight [kDa]	ng/µg	fg/µm²	molecules/µm²	molecules/cell	RSD (%)	ng/µg	fg/µm²	molecules/µm²	molecules/cell	RSD (%)
BSU37250	narI	25.296	0.006	2.05E-07	0.005	0.212	6.28					
BSU37320	narK	42.925	0.023	7.82E-07	0.011	0.476	31.24					
BSU02750	natA	27.878	0.003	1.16E-07	0.003	0.109	19.00	0.009	5.00E-07	0.011	0.488	20.64
BSU02760	natB	42.792	0.007	2.33E-07	0.003	0.142	20.73	0.016	9.20E-07	0.013	0.585	7.39
BSU02730	natK	35.076	0.004	1.24E-07	0.002	0.092	2.46	0.005	2.59E-07	0.004	0.201	64.09
BSU12290	ndh	41.953	0.054	1.82E-06	0.026	1.134	12.38	0.167	9.60E-06	0.138	6.225	18.53
BSU33420	nhaK	75.227	0.016	5.43E-07	0.004	0.189	33.21	0.019	1.08E-06	0.009	0.390	55.23
BSU02950	niaP	43.708	0.006	2.09E-07	0.003	0.125	6.56	0.005	3.01E-07	0.004	0.187	36.77
BSU03420	nin	14.997	0.073	2.48E-06	0.100	4.324	34.77	0.202	1.16E-05	0.465	21.021	41.72
BSU36520	nrgB	12.822	0.001	4.99E-08	0.002	0.102	32.81	0.001	6.83E-08	0.003	0.145	41.78
BSU03430	nucA	16.486	0.091	3.08E-06	0.112	4.875	33.67	0.222	1.27E-05	0.465	20.987	42.52
BSU39410	nupC	42.495	0.105	3.56E-06	0.051	2.189	17.99	0.131	7.49E-06	0.106	4.794	1.56
BSU39020	nupG	43.719	0.024	8.01E-07	0.011	0.478	30.61	0.028	1.60E-06	0.022	0.998	35.93
BSU31550	nupO	56.299	0.001	4.21E-08	0.000	0.020	29.13					
BSU31570	nupQ	33.738	0.001	1.90E-08	0.000	0.015	19.25					
BSU11440	oppB	34.074	0.440	1.50E-05	0.264	11.459	0.41	0.389	2.23E-05	0.395	17.837	19.57
BSU11450	oppC	33.621	0.351	1.19E-05	0.214	9.259	13.03	0.313	1.79E-05	0.321	14.522	18.95
BSU11460	oppD	39.395	0.135	4.60E-06	0.070	3.045	3.76	0.146	8.39E-06	0.128	5.794	22.90
BSU11470	oppF	34.748	0.059	2.02E-06	0.035	1.518	11.35	0.080	4.57E-06	0.079	3.579	16.65
BSU02980	opuAA	46.468	0.009	3.19E-07	0.004	0.179	43.97	0.044	2.55E-06	0.033	1.491	20.24
BSU02990	opuAB	30.247	0.021	7.22E-07	0.014	0.623	31.36	0.049	2.80E-06	0.056	2.519	40.32
BSU03000	opuAC	32.215	0.018	6.22E-07	0.012	0.504	27.46	0.040	2.28E-06	0.043	1.922	26.23
BSU33830	opuCA	43.247	0.004	1.42E-07	0.002	0.086	56.16	0.001	4.04E-08	0.001	0.025	26.32
BSU33810	opuCC	34.216	0.004	1.21E-07	0.002	0.092	18.13	0.001	7.09E-08	0.001	0.056	29.50
BSU30070	opuD	56.122	0.022	7.48E-07	0.008	0.348	15.08	0.022	1.26E-06	0.014	0.611	40.93
BSU40970	parA	27.543	0.019	6.48E-07	0.014	0.614	27.43	0.038	2.17E-06	0.048	2.148	23.93
BSU25000	pbpA	80.141	0.329	1.12E-05	0.084	3.637	8.89	0.550	3.15E-05	0.237	10.705	8.47
BSU15160	pbpB	78.947	0.079	2.69E-06	0.021	0.890	4.84	0.131	7.51E-06	0.057	2.587	6.44

			Control					1% subtilin				
Accession number	Locus tag	Mol. Weight [kDa]	ng/µg	fg/µm²	molecules/µm²	molecules/cell	RSD (%)	ng/µg	fg/µm²	molecules/µm²	molecules/cell	RSD (%)
BSU04140	pbpC	74.405	0.234	7.94E-06	0.064	2.786	12.27	0.489	2.80E-05	0.227	10.251	10.74
BSU31490	pbpD	70.425	0.109	3.69E-06	0.032	1.367	16.98	0.279	1.60E-05	0.137	6.189	10.64
BSU10110	pbpF	79.264	0.009	3.06E-07	0.002	0.101	3.61	0.014	7.98E-07	0.006	0.274	10.51
BSU13980	pbpH	79.347	0.029	9.85E-07	0.007	0.324	8.14	0.064	3.70E-06	0.028	1.268	13.24
BSU16950	pbpX	43.857	0.058	1.95E-06	0.027	1.163	4.52	0.182	1.04E-05	0.143	6.478	15.90
BSU06370	pbuG	46.191	0.015	5.08E-07	0.007	0.287	**44.45**	0.028	1.61E-06	0.021	0.946	5.16
BSU29990	pbuO	45.419	0.009	2.95E-07	0.004	0.169	10.05	0.017	9.67E-07	0.013	0.579	18.37
BSU14590	pdhB	35.474	0.136	4.63E-06	0.079	3.407	17.66	0.327	1.88E-05	0.319	14.403	12.63
BSU13850	pfeT	68.565	0.044	1.50E-06	0.013	0.572	10.97	0.076	4.36E-06	0.038	1.728	9.25
BSU28910	pftA	15.711	0.007	2.32E-07	0.009	0.386	**64.43**	0.476	2.73E-05	1.048	47.329	23.58
BSU28900	pftB	23.949	0.007	2.25E-07	0.006	0.246	**78.83**	0.114	6.53E-06	0.164	7.417	29.02
BSU25330	pgpH	79.053	0.136	4.61E-06	0.035	1.522	8.39	0.228	1.31E-05	0.100	4.497	4.83
BSU16920	pgsA	21.338	0.029	9.83E-07	0.028	1.202	**38.22**	0.054	3.12E-06	0.088	3.973	30.90
BSU29100	phoR	65.121	0.213	7.25E-06	0.067	2.906	6.33	0.280	1.61E-05	0.148	6.709	13.16
BSU12840	pit	35.26	0.053	1.81E-06	0.031	1.341	**36.03**	0.074	4.22E-06	0.072	3.254	**51.65**
BSU18070	plsY	20.966	0.119	4.06E-06	0.117	5.050	14.81	0.163	9.37E-06	0.269	12.153	8.18
BSU22320	ponA	99.561	0.114	3.87E-06	0.023	1.015	10.36	0.275	1.58E-05	0.095	4.311	11.59
BSU18830	pps	97.191	0.000	1.68E-08	0.000	0.005	15.34	0.004	2.33E-07	0.001	0.065	19.26
BSU15770	prkC	71.866	0.083	2.83E-06	0.024	1.028	8.27	0.160	9.19E-06	0.077	3.478	16.67
BSU09950	prsA	32.509	1.397	4.74E-05	0.879	38.100	8.71	2.404	1.38E-04	2.555	115.417	8.42
BSU02290	psd	29.689	0.010	3.35E-07	0.007	0.295	16.61	0.023	1.33E-06	0.027	1.216	11.04
BSU02270	pssA	19.613						0.009	5.17E-07	0.016	0.717	29.67
BSU24970	pstA	31.436	0.011	3.90E-07	0.007	0.324	26.20	0.072	4.14E-06	0.079	3.587	20.28
BSU24960	pstBA	30.002	0.013	4.36E-07	0.009	0.380	8.43	0.073	4.18E-06	0.084	3.792	8.60
BSU24950	pstBB	29.198	0.010	3.50E-07	0.007	0.313	**33.93**	0.074	4.27E-06	0.088	3.976	7.49
BSU24980	pstC	33.193	0.009	2.98E-07	0.005	0.234	28.57	0.048	2.78E-06	0.050	2.277	8.74
BSU24990	pstS	31.683	0.314	1.07E-05	0.203	8.797	5.94	0.612	3.51E-05	0.667	30.131	10.75
BSU36250	ptkA	24.619	0.001	3.11E-08	0.001	0.033	21.31	0.008	4.66E-07	0.011	0.515	13.44

			Control					1% subtilin				
Accession number	Locus tag	Mol. Weight [kDa]	ng/µg	fg/µm²	molecules/µm²	molecules/cell	RSD (%)	ng/µg	fg/µm²	molecules/µm²	molecules/cell	RSD (%)
BSU13890	ptsG	75.524	1.049	3.56E-05	0.284	12.316	2.18	2.358	1.35E-04	1.079	48.735	9.63
BSU06430	purK	42.319	0.015	4.98E-07	0.007	0.307	4.74	0.024	1.35E-06	0.019	0.871	20.90
BSU02230	purT	42.093	0.002	5.58E-08	0.001	0.035	2.99	0.006	3.21E-07	0.005	0.207	20.47
BSU03220	putP	51.403	0.445	1.51E-05	0.177	7.684	11.11	0.291	1.67E-05	0.195	8.822	17.43
BSU15480	pyrP	45.452	0.016	5.47E-07	0.007	0.314	11.90	0.038	2.17E-06	0.029	1.302	27.17
BSU22560	qcrA	18.736	0.076	2.57E-06	0.083	3.578	20.32	0.037	2.10E-06	0.067	3.046	4.52
BSU22550	qcrB	25.488	0.076	2.59E-06	0.061	2.651	4.79	0.063	3.61E-06	0.085	3.852	18.07
BSU22540	qcrC	28.161	0.041	1.39E-06	0.030	1.287	10.44	0.032	1.84E-06	0.039	1.778	14.07
BSU38170	qoxA	35.908	1.895	6.44E-05	1.080	46.797	10.03	2.614	1.50E-04	2.515	113.616	9.87
BSU38160	qoxB	73.837	2.435	8.27E-05	0.675	29.251	19.90	3.295	1.89E-04	1.541	69.636	18.76
BSU38150	qoxC	22.671	0.209	7.11E-06	0.189	8.187	54.81	0.362	2.08E-05	0.552	24.933	73.87
BSU38140	qoxD	13.687	0.010	3.33E-07	0.015	0.635	44.40	0.021	1.22E-06	0.054	2.418	22.85
BSU00870	radA	49.482	0.017	5.94E-07	0.007	0.313	10.76	0.027	1.54E-06	0.019	0.847	31.32
BSU16560	rasP	46.497	0.110	3.73E-06	0.048	2.095	6.08	0.187	1.07E-05	0.139	6.284	9.42
BSU23150	resA	20.008	0.088	3.00E-06	0.090	3.910	9.17	0.074	4.25E-06	0.128	5.774	1.95
BSU23140	resB	61.76	0.136	4.62E-06	0.045	1.951	9.49	0.116	6.67E-06	0.065	2.936	8.94
BSU23130	resC	43.69	0.137	4.67E-06	0.064	2.787	19.88	0.116	6.67E-06	0.092	4.154	12.92
BSU23110	resE	66.77	0.177	6.02E-06	0.054	2.353	10.27	0.185	1.06E-05	0.096	4.327	5.85
BSU23050	ribU	20.553	0.001	4.74E-08	0.001	0.060	96.39	0.004	2.05E-07	0.006	0.271	24.89
BSU37760	rocC	51.73	0.032	1.09E-06	0.013	0.550	29.77	0.047	2.71E-06	0.032	1.425	21.79
BSU40330	rocE	51.634	0.847	2.88E-05	0.336	14.557	10.79	1.468	8.42E-05	0.982	44.371	8.86
BSU38120	rodA	43.274	0.042	1.44E-06	0.020	0.868	15.51	0.063	3.64E-06	0.051	2.290	32.32
BSU01490	rplM	16.375	0.016	5.30E-07	0.020	0.846	13.87	0.043	2.44E-06	0.090	4.055	7.66
BSU23090	rsiX	41.294	0.045	1.52E-06	0.022	0.960	15.45	0.142	8.14E-06	0.119	5.364	11.00
BSU38050	sacP	49.396	0.052	1.76E-06	0.022	0.932	22.78	0.116	6.64E-06	0.081	3.655	6.34
BSU38410	sacX	49.024	0.001	4.95E-08	0.001	0.026	67.14	0.003	2.00E-07	0.002	0.111	39.67
BSU01540	salA	38.638	0.148	5.02E-06	0.078	3.389	12.29	0.290	1.66E-05	0.259	11.703	5.13
BSU06650	sapB	25.709	0.006	2.20E-07	0.005	0.224	16.89	0.008	4.81E-07	0.011	0.509	22.61

Accession number	Locus tag	Mol. Weight [kDa]	Control					1% subtilin				
			ng/µg	fg/µm²	molecules/µm²	molecules/cell	RSD (%)	ng/µg	fg/µm²	molecules/µm²	molecules/cell	RSD (%)
BSU21750	sco	21.703	0.036	1.22E-06	0.034	1.470	10.31	0.024	1.36E-06	0.038	1.710	15.56
BSU28430	sdhB	28.418	1.237	4.20E-05	0.891	38.620	5.85	1.661	9.53E-05	2.020	91.243	29.64
BSU28450	sdhC	22.931	0.154	5.24E-06	0.138	5.962	44.12	0.270	1.55E-05	0.406	18.354	30.10
BSU27650	secDF	81.653	1.016	3.45E-05	0.254	11.031	3.57	1.677	9.62E-05	0.710	32.058	4.97
BSU33630	secG	7.9957	0.022	7.62E-07	0.057	2.487	27.49	0.043	2.46E-06	0.186	8.382	10.53
BSU01360	secY	47.242	0.386	1.31E-05	0.167	7.240	25.04	0.630	3.62E-05	0.461	20.821	18.27
BSU29805	sftA	106.82	0.010	3.50E-07	0.002	0.086	5.91	0.037	2.12E-06	0.012	0.540	6.51
BSU23100	sigX	23.223	0.028	9.48E-07	0.025	1.066	20.73	0.096	5.51E-06	0.143	6.461	4.39
BSU23310	sipS	21.047	0.128	4.35E-06	0.125	5.401	7.18	0.247	1.42E-05	0.406	18.350	9.33
BSU14410	sipT	21.854	0.033	1.11E-06	0.031	1.331	7.03	0.060	3.43E-06	0.095	4.276	9.95
BSU16800	spoIIIE	87.18	0.042	1.41E-06	0.010	0.422	9.24	0.074	4.22E-06	0.029	1.318	11.85
BSU41040	spoIIIJ	29.521	0.300	1.02E-05	0.208	9.016	33.60	0.648	3.72E-05	0.759	34.278	26.41
BSU29530	sppA	36.673	0.272	9.26E-06	0.152	6.589	2.72	0.378	2.17E-05	0.356	16.099	21.35
BSU12860	steT	47.148	0.027	9.31E-07	0.012	0.516	33.29	0.053	3.06E-06	0.039	1.763	15.35
BSU16480	swrB	19.119	0.005	1.55E-07	0.005	0.211	6.93	0.007	4.16E-07	0.013	0.592	58.69
BSU06710	swrC	113.5	0.557	1.89E-05	0.100	4.349	13.97	0.964	5.53E-05	0.294	13.262	8.61
BSU35750	tagA	29.499	0.011	3.67E-07	0.007	0.325	12.38	0.030	1.73E-06	0.035	1.594	18.15
BSU35760	tagB	44.554	0.023	7.69E-07	0.010	0.450	9.20	0.045	2.57E-06	0.035	1.568	3.92
BSU35720	tagF	88.062	0.308	1.05E-05	0.072	3.104	4.11	0.558	3.20E-05	0.219	9.890	13.97
BSU35710	tagG	32.184	0.051	1.75E-06	0.033	1.417	11.29	0.096	5.51E-06	0.103	4.655	22.26
BSU35700	tagH	59.243	0.085	2.90E-06	0.030	1.280	5.59	0.166	9.51E-06	0.097	4.366	16.84
BSU35530	tagO	39.362	0.002	8.12E-08	0.001	0.054	31.28	0.025	1.46E-06	0.022	1.009	29.16
BSU35650	tagU	34.586	0.188	6.39E-06	0.111	4.823	9.58	0.269	1.54E-05	0.269	12.138	9.95
BSU35520	tagV	43.211	0.103	3.51E-06	0.049	2.120	13.05	0.231	1.32E-05	0.184	8.334	15.09
BSU05990	tatCY	28.667	0.009	2.90E-07	0.006	0.264	51.83	0.017	9.99E-07	0.021	0.949	11.93
BSU03600	tcyB	26.388	0.055	1.87E-06	0.043	1.845	12.08	0.126	7.21E-06	0.165	7.438	8.09
BSU03590	tcyC	27.748	0.066	2.24E-06	0.049	2.103	7.06	0.128	7.33E-06	0.159	7.191	14.67
BSU09130	tcyP	48.982	0.009	2.95E-07	0.004	0.157	44.21	0.009	5.39E-07	0.007	0.299	16.70

			Control					1% subtilin				
Accession number	Locus tag	Mol. Weight [kDa]	ng/µg	fg/µm²	molecules/µm²	molecules/cell	RSD (%)	ng/µg	fg/µm²	molecules/µm²	molecules/cell	RSD (%)
BSU36260	tkmA	26.64	0.013	4.32E-07	0.010	0.423	12.86	0.039	2.21E-06	0.050	2.258	13.35
BSU03440	tlpC	61.783	0.000	1.56E-08	0.000	0.007	**39.18**	0.001	8.50E-08	0.001	0.037	**41.22**
BSU07800	treP	49.999	0.382	1.30E-05	0.156	6.770	5.10	0.863	4.95E-05	0.596	26.943	12.56
BSU05920	tsaB	25.243	0.000	4.50E-09	0.000	0.005	**40.76**	0.003	1.47E-07	0.004	0.159	12.21
BSU22380	tseB	17.896	0.012	3.92E-07	0.013	0.572	10.64	0.032	1.86E-06	0.063	2.829	13.61
BSU35560	tuaF	25.134	0.002	7.70E-08	0.002	0.080	22.02	0.006	3.71E-07	0.009	0.401	6.73
BSU31150	uppP	30.345	0.067	2.26E-06	0.045	1.948	**36.85**	0.093	5.35E-06	0.106	4.793	15.97
BSU40390	walH	52.201	0.048	1.64E-06	0.019	0.819	10.15	0.090	5.16E-06	0.060	2.692	9.18
BSU40400	walK	70.033	0.074	2.51E-06	0.022	0.935	3.54	0.135	7.77E-06	0.067	3.018	9.86
BSU00250	xpaC	23.959	0.015	4.94E-07	0.012	0.539	11.91	0.018	1.02E-06	0.026	1.156	4.03
BSU00570	yabM	57.356	0.001	2.74E-08	0.000	0.012	**40.42**	0.002	1.22E-07	0.001	0.058	**41.22**
BSU00720	yacD	34.096	0.157	5.32E-06	0.094	4.075	4.83	0.295	1.69E-05	0.299	13.500	15.23
BSU00890	yacL	40.858	0.093	3.18E-06	0.047	2.029	10.17	0.125	7.16E-06	0.106	4.770	20.27
BSU01460	ybaE	32.208	0.015	5.14E-07	0.010	0.417	17.76	0.033	1.90E-06	0.035	1.601	**35.81**
BSU01470	ybaF	29.784	0.009	2.98E-07	0.006	0.261	11.32	0.010	5.81E-07	0.012	0.531	**51.83**
BSU01580	ybaR	51.173	0.003	8.88E-08	0.001	0.045	29.57	0.013	7.39E-07	0.009	0.393	27.23
BSU01590	ybaS	34.714	0.002	6.70E-08	0.001	0.050	**62.58**	0.008	4.35E-07	0.008	0.341	24.24
BSU02170	ybfB	45.235	0.001	2.52E-08	0.000	0.015	23.69					
BSU02190	ybfF	35.309	0.052	1.77E-06	0.030	1.307	7.07	0.087	4.96E-06	0.085	3.824	8.10
BSU01450	ybxA	31.458	0.009	2.93E-07	0.006	0.243	7.51	0.017	9.58E-07	0.018	0.829	24.41
BSU02560	ycbM	35.28	0.000	6.81E-09	0.000	0.005	14.32	0.001	3.66E-08	0.001	0.028	13.51
BSU02570	ycbN	34.503	0.001	4.78E-08	0.001	0.036	14.76	0.004	2.45E-07	0.004	0.193	5.97
BSU02920	yceF	29.167	0.007	2.53E-07	0.005	0.227	**37.27**	0.014	8.17E-07	0.017	0.762	19.60
BSU03020	ycgA	50.552	0.020	6.64E-07	0.008	0.343	26.41	0.013	7.51E-07	0.009	0.404	21.68
BSU03240	ycgQ	33.207	0.009	3.02E-07	0.005	0.237	7.74	0.016	9.01E-07	0.016	0.738	16.44
BSU03250	ycgR	32.49						0.001	5.08E-08	0.001	0.043	**36.02**
BSU03370	yckA	25.229	0.006	1.88E-07	0.004	0.195	21.88	0.013	7.58E-07	0.018	0.818	20.86
BSU03940	ycnI	22.113	0.032	1.10E-06	0.030	1.302	18.23	0.051	2.91E-06	0.079	3.578	28.58

			Control					1% subtilin				
Accession number	Locus tag	Mol. Weight [kDa]	ng/µg	fg/µm²	molecules/µm²	molecules/cell	RSD (%)	ng/µg	fg/µm²	molecules/µm²	molecules/cell	RSD (%)
BSU03950	ycnJ	59.794	0.021	6.99E-07	0.007	0.305	27.10	0.024	1.36E-06	0.014	0.617	32.52
BSU03970	ycnL	12.975	0.002	7.09E-08	0.003	0.143	16.67	0.004	2.37E-07	0.011	0.496	45.05
BSU04060	ycsG	42.134	0.129	4.38E-06	0.063	2.716	2.87	0.084	4.80E-06	0.069	3.102	12.74
BSU03540	ycxB	22.034	0.001	3.59E-08	0.001	0.042	5.23	0.008	4.33E-07	0.012	0.534	13.97
BSU02710	yczC	13.95	0.001	3.57E-08	0.002	0.067	24.21	0.004	2.34E-07	0.010	0.456	10.65
BSU04310	ydaN	77.207	0.001	2.95E-08	0.000	0.010	31.57	0.003	1.95E-07	0.002	0.069	37.63
BSU04340	ydaP	63.138	0.000	8.96E-09	0.000	0.004	17.88	0.001	6.54E-08	0.001	0.028	10.18
BSU04480	ydbI	40.073	0.039	1.32E-06	0.020	0.863	45.18	0.082	4.72E-06	0.071	3.205	7.64
BSU04490	ydbJ	34.276	0.045	1.52E-06	0.027	1.155	27.91	0.129	7.39E-06	0.130	5.866	4.90
BSU04500	ydbK	28.926	0.022	7.47E-07	0.016	0.674	35.22	0.050	2.85E-06	0.059	2.680	34.81
BSU04590	ydbS	18.122	0.149	5.07E-06	0.169	7.305	26.20	0.178	1.02E-05	0.340	15.364	8.57
BSU04600	ydbT	56.827	0.100	3.39E-06	0.036	1.559	11.73	0.135	7.74E-06	0.082	3.704	6.00
BSU04610	ydcA	22.544	0.001	3.38E-08	0.001	0.039	5.97	0.003	1.73E-07	0.005	0.209	28.14
BSU05190	ydeG	44.047	0.005	1.61E-07	0.002	0.095	28.59	0.007	3.92E-07	0.005	0.242	18.69
BSU05280	ydeO	31.428	0.070	2.38E-06	0.046	1.980	8.38	0.122	6.98E-06	0.134	6.042	5.72
BSU05410	ydfH	43.985	0.001	2.38E-08	0.000	0.014	45.79	0.003	1.49E-07	0.002	0.092	26.43
BSU05470	ydfM	32.854	0.002	7.64E-08	0.001	0.061	17.24	0.009	5.10E-07	0.009	0.422	6.44
BSU05620	ydgF	50.789	0.031	1.04E-06	0.012	0.534	36.27	0.038	2.21E-06	0.026	1.181	35.66
BSU06380	yebC	30.618	0.039	1.32E-06	0.026	1.129	15.77	0.073	4.16E-06	0.082	3.701	8.25
BSU06400	yebE	20.666	0.019	6.35E-07	0.018	0.802	6.70	0.030	1.74E-06	0.051	2.297	11.77
BSU08460	yfhA	35.955	0.002	6.04E-08	0.001	0.044	30.28					
BSU08440	yfiY	36.304	0.415	1.41E-05	0.234	10.130	15.66	0.483	2.77E-05	0.460	20.764	14.25
BSU08450	yfiZ	35.135	0.007	2.23E-07	0.004	0.165	1.97	0.010	5.89E-07	0.010	0.456	12.66
BSU08000	yfjQ	37.845	0.005	1.66E-07	0.003	0.115	10.69	0.009	5.16E-07	0.008	0.371	7.41
BSU07940	yfkC	31.918	0.013	4.40E-07	0.008	0.360	11.49	0.026	1.48E-06	0.028	1.263	18.92
BSU07860	yfkL	42.168	0.004	1.38E-07	0.002	0.086	17.89	0.009	4.95E-07	0.007	0.319	10.76
BSU07570	yflS	51.431	0.005	1.64E-07	0.002	0.083	44.75	0.008	4.85E-07	0.006	0.257	12.15
BSU07460	yfmI	45.606	0.002	6.20E-08	0.001	0.035	9.88	0.005	2.86E-07	0.004	0.171	26.87

			Control					1% subtilin				
Accession number	Locus tag	Mol. Weight [kDa]	ng/µg	fg/µm²	molecules/µm²	molecules/cell	RSD (%)	ng/µg	fg/µm²	molecules/µm²	molecules/cell	RSD (%)
BSU07450	yfmJ	36.662						0.004	2.29E-07	0.004	0.170	32.01
BSU07400	yfmO	41.45	0.016	5.28E-07	0.008	0.332	9.96	0.037	2.12E-06	0.031	1.388	15.34
BSU07380	yfmQ	16.865	0.045	1.52E-06	0.054	2.348	18.19	0.060	3.41E-06	0.122	5.506	5.25
BSU07320	yfnC	43.488	0.008	2.56E-07	0.004	0.154	67.00	0.024	1.39E-06	0.019	0.868	23.83
BSU07260	yfnI	73.314	0.019	6.62E-07	0.005	0.236	7.56	0.126	7.24E-06	0.059	2.686	8.36
BSU08690	ygaD	65.767	0.033	1.10E-06	0.010	0.439	11.49	0.080	4.57E-06	0.042	1.891	60.21
BSU08700	ygaE	40.734	0.035	1.17E-06	0.017	0.752	22.86	0.068	3.91E-06	0.058	2.614	16.18
BSU08890	ygaO	17.773	0.009	3.00E-07	0.010	0.441	33.35	0.014	8.31E-07	0.028	1.272	12.87
BSU08740	ygzB	13.192	0.002	5.84E-08	0.003	0.116	36.39	0.002	1.31E-07	0.006	0.270	27.64
BSU09970	yhaJ	19.64						0.001	6.55E-08	0.002	0.091	20.59
BSU09900	yhaP	45.475	0.033	1.11E-06	0.015	0.637	7.94	0.066	3.78E-06	0.050	2.262	10.53
BSU09890	yhaQ	33.858	0.008	2.56E-07	0.005	0.197	22.84	0.011	6.25E-07	0.011	0.502	13.64
BSU09000	yhbJ	22.347	1.602	5.44E-05	1.466	63.563	13.93	2.755	1.58E-04	4.258	192.370	9.63
BSU09010	yhcA	58.341	0.332	1.13E-05	0.117	5.052	15.84	0.518	2.97E-05	0.307	13.852	20.41
BSU09030	yhcC	13.958	0.123	4.17E-06	0.180	7.806	11.71	0.193	1.11E-05	0.478	21.604	20.73
BSU09070	yhcG	26.52	0.004	1.36E-07	0.003	0.134	24.96	0.011	6.40E-07	0.015	0.657	17.46
BSU09080	yhcH	34.47	0.049	1.65E-06	0.029	1.252	10.92	0.105	6.05E-06	0.106	4.775	5.63
BSU09090	yhcI	34.881	0.045	1.53E-06	0.026	1.142	14.82	0.082	4.70E-06	0.081	3.669	32.37
BSU09550	yhdP	49.855	0.148	5.03E-06	0.061	2.633	12.20	0.278	1.59E-05	0.192	8.692	16.55
BSU09640	yhdY	42.537	0.006	1.96E-07	0.003	0.120	30.00	0.013	7.50E-07	0.011	0.480	26.65
BSU09790	yheB	42.908	0.066	2.23E-06	0.031	1.354	4.72	0.127	7.30E-06	0.102	4.628	11.88
BSU10160	yhgE	84.093	0.050	1.70E-06	0.012	0.526	4.56	0.098	5.62E-06	0.040	1.818	18.91
BSU23890	yidC2	30.747	0.029	9.82E-07	0.019	0.833	24.25	0.033	1.87E-06	0.037	1.656	43.29
BSU11180	yitZ	20.023	0.005	1.61E-07	0.005	0.209	16.08	0.011	6.07E-07	0.018	0.824	10.72
BSU11640	yjbQ	67.467	0.008	2.63E-07	0.002	0.102	10.61	0.026	1.51E-06	0.013	0.609	25.20
BSU11900	yjcL	43.321	0.007	2.36E-07	0.003	0.142	18.86	0.004	2.39E-07	0.003	0.150	15.46
BSU12880	ykcB	76.341	0.001	3.26E-08	0.000	0.011	19.21	0.002	1.23E-07	0.001	0.044	12.76
BSU13000	ykfD	37.285	0.002	6.31E-08	0.001	0.044	10.38	0.001	8.00E-08	0.001	0.058	29.18

Accession number	Locus tag	Mol. Weight [kDa]	Control					1% subtilin				
			ng/µg	fg/µm²	molecules/µm²	molecules/cell	RSD (%)	ng/µg	fg/µm²	molecules/µm²	molecules/cell	RSD (%)
BSU14340	yknW	24.175	0.166	5.63E-06	0.140	6.078	10.72	0.226	1.30E-05	0.323	14.612	9.53
BSU14360	yknY	25.272	0.004	1.21E-07	0.003	0.125	23.19	0.006	3.55E-07	0.008	0.382	9.35
BSU14370	yknZ	42.124	0.026	8.89E-07	0.013	0.551	**39.52**	0.062	3.54E-06	0.051	2.288	11.95
BSU13430	ykoX	24.94	0.006	2.13E-07	0.005	0.223	12.99	0.016	9.07E-07	0.022	0.990	5.67
BSU14440	ykpB	33.572						0.002	1.05E-07	0.002	0.085	27.85
BSU14210	ykuT	29.956	0.005	1.76E-07	0.004	0.153	12.91	0.015	8.73E-07	0.018	0.793	8.93
BSU14770	ylaG	68.388	0.156	5.29E-06	0.047	2.018	25.38	0.881	5.06E-05	0.445	20.114	5.75
BSU15340	ylmA	29.699						0.001	3.44E-08	0.001	0.032	27.38
BSU15400	ylmG	10.307	0.005	1.84E-07	0.011	0.465	26.73	0.014	7.97E-07	0.047	2.105	**31.70**
BSU17910	yneF	8.2993	0.259	8.80E-06	0.639	27.685	6.56	0.465	2.67E-05	1.937	87.517	9.72
BSU17950	yneJ	18.126	0.011	3.82E-07	0.013	0.550	29.40	0.022	1.27E-06	0.042	1.901	16.26
BSU17960	yneK	16.989	0.036	1.22E-06	0.043	1.868	3.27	0.045	2.59E-06	0.092	4.149	2.07
BSU18190	yngC	21.926	0.021	7.12E-07	0.020	0.847	30.68	0.051	2.91E-06	0.080	3.605	12.55
BSU19340	yocR	48.333	0.007	2.44E-07	0.003	0.132	15.26	0.018	1.04E-06	0.013	0.585	19.39
BSU18370	yoeA	51.305	0.019	6.59E-07	0.008	0.335	19.12	0.032	1.81E-06	0.021	0.961	10.05
BSU19390	yojN	32.579	0.004	1.23E-07	0.002	0.099	8.54	0.014	7.76E-07	0.014	0.648	10.19
BSU19140	yozB	20.498	0.029	9.78E-07	0.029	1.245	23.79	0.042	2.40E-06	0.070	3.182	29.01
BSU22840	yphC	48.769	0.004	1.46E-07	0.002	0.078	13.30	0.025	1.45E-06	0.018	0.811	20.55
BSU22810	yphF	27.747	0.010	3.47E-07	0.008	0.327	10.31	0.012	6.90E-07	0.015	0.677	19.63
BSU22510	ypjC	31.96	0.008	2.56E-07	0.005	0.209	29.64	0.010	5.84E-07	0.011	0.497	16.44
BSU25390	yqeZ	46.462	0.078	2.65E-06	0.034	1.491	**32.00**	0.068	3.92E-06	0.051	2.294	6.72
BSU25370	yqfB	15.77	0.011	3.68E-07	0.014	0.610	5.51	0.004	2.11E-07	0.008	0.365	**52.61**
BSU25110	yqfU	32.518	0.019	6.56E-07	0.012	0.527	15.24	0.026	1.51E-06	0.028	1.265	10.98
BSU25050	yqgA	15.353	0.002	7.50E-08	0.003	0.128	14.00	0.004	2.26E-07	0.009	0.401	30.53
BSU25040	yqgB	28.512	0.018	6.10E-07	0.013	0.559	5.75	0.033	1.88E-06	0.040	1.794	10.77
BSU24870	yqgP	56.461	0.018	5.97E-07	0.006	0.276	15.77	0.040	2.31E-06	0.025	1.113	7.96
BSU24840	yqgS	73.211	0.041	1.40E-06	0.012	0.499	11.04	0.071	4.05E-06	0.033	1.506	18.11
BSU24500	yqhP	14.567	0.016	5.58E-07	0.023	1.001	28.59	0.018	1.05E-06	0.043	1.955	24.77

Accession number	Locus tag	Mol. Weight [kDa]	Control					1% subtilin				
			ng/µg	fg/µm²	molecules/µm²	molecules/cell	RSD (%)	ng/µg	fg/µm²	molecules/µm²	molecules/cell	RSD (%)
BSU24490	yqhQ	35.942	0.020	6.77E-07	0.011	0.492	12.12	0.044	2.53E-06	0.042	1.914	17.32
BSU23950	yqjA	37.031	0.018	5.95E-07	0.010	0.419	2.27	0.026	1.50E-06	0.024	1.100	9.83
BSU26860	yraO	46.727	0.002	5.58E-08	0.001	0.031	10.89	0.002	1.05E-07	0.001	0.061	34.64
BSU27700	yrbF	9.7723	0.605	2.05E-05	1.266	54.871	13.25	0.857	4.92E-05	3.030	136.908	11.55
BSU27200	yrhG	28.488	0.037	1.25E-06	0.026	1.142	25.96	0.096	5.48E-06	0.116	5.232	35.04
BSU27100	yrhP	23.389	0.003	9.59E-08	0.002	0.107	23.32	0.016	9.29E-07	0.024	1.081	9.63
BSU26440	yrkO	46.431	0.001	2.03E-08	0.000	0.011	10.81	0.001	7.92E-08	0.001	0.046	28.51
BSU27630	yrvD	12.234	0.036	1.21E-06	0.060	2.585	22.85	0.055	3.15E-06	0.155	6.995	25.01
BSU28570	yshE	15.012	0.012	4.02E-07	0.016	0.699	15.12	0.021	1.19E-06	0.048	2.148	12.94
BSU29520	yteJ	19.119	0.046	1.55E-06	0.049	2.119	12.07	0.070	4.01E-06	0.126	5.705	20.08
BSU30010	ythP	26.506	0.003	1.09E-07	0.002	0.108	32.17	0.007	4.18E-07	0.009	0.429	45.89
BSU30000	ythQ	45.047	0.002	7.60E-08	0.001	0.044	15.83	0.004	2.32E-07	0.003	0.140	54.30
BSU30680	ytjA	8.491	0.001	2.55E-08	0.002	0.078	24.77	0.001	8.27E-08	0.006	0.265	1.76
BSU30530	ytnA	50.327	0.052	1.78E-06	0.021	0.923	19.66	0.107	6.14E-06	0.073	3.318	21.74
BSU29780	ytxG	15.442	0.156	5.30E-06	0.207	8.954	20.10	0.145	8.33E-06	0.325	14.682	13.68
BSU31020	yuaF	20.585	0.010	3.56E-07	0.010	0.451	28.89	0.011	6.07E-07	0.018	0.803	57.95
BSU31160	yubA	43.61	0.066	2.26E-06	0.031	1.350	15.05	0.116	6.64E-06	0.092	4.144	26.83
BSU31110	yubF	10.011	0.049	1.67E-06	0.101	4.358	24.47	0.139	7.95E-06	0.478	21.611	21.36
BSU32080	yuiB	11.728	0.003	9.00E-08	0.005	0.200	47.89	0.005	2.64E-07	0.014	0.612	45.64
BSU32060	yuiD	17.182	0.011	3.59E-07	0.013	0.545	15.63	0.023	1.30E-06	0.046	2.061	19.66
BSU32040	yuiF	45.937	0.004	1.34E-07	0.002	0.076	36.81	0.023	1.30E-06	0.017	0.769	35.64
BSU32110	yumC	36.841	0.000	1.26E-08	0.000	0.009	73.86	0.004	2.26E-07	0.004	0.167	14.23
BSU32380	yunE	29.097	0.002	6.13E-08	0.001	0.055	41.70	0.002	1.37E-07	0.003	0.128	54.93
BSU32940	yusV	30.306	0.009	3.01E-07	0.006	0.259	26.86	0.018	1.04E-06	0.021	0.931	40.11
BSU32200	yutJ	39.604	0.013	4.58E-07	0.007	0.302	16.51	0.027	1.58E-06	0.024	1.082	7.38
BSU32180	yutK	42.284	0.070	2.38E-06	0.034	1.468	18.49	0.151	8.65E-06	0.123	5.567	10.21
BSU31500	yuxK	15.728	0.017	5.74E-07	0.022	0.953	27.56	0.020	1.14E-06	0.043	1.964	30.88
BSU33870	yvbI	25.753	0.009	2.96E-07	0.007	0.300	39.91	0.022	1.25E-06	0.029	1.320	7.81

Accession number	Locus tag	Mol. Weight [kDa]	Control					1% subtilin				
			ng/µg	fg/µm²	molecules/µm²	molecules/cell	RSD (%)	ng/µg	fg/µm²	molecules/µm²	molecules/cell	RSD (%)
BSU33360	yvgJ	70.745	0.000	1.44E-08	0.000	0.005	24.30	0.008	4.71E-07	0.004	0.181	9.90
BSU35280	yvjA	29.984	0.014	4.88E-07	0.010	0.425	21.94	0.008	4.84E-07	0.010	0.439	25.72
BSU34980	yvoD	33.602	0.004	1.43E-07	0.003	0.111	37.15	0.010	5.97E-07	0.011	0.484	16.41
BSU33140	yvqJ	45.896	0.001	2.53E-08	0.000	0.014	29.03	0.001	5.14E-08	0.001	0.030	23.36
BSU33160	yvrA	48.762	0.001	3.43E-08	0.000	0.018	22.35	0.002	1.43E-07	0.002	0.080	28.22
BSU33180	yvrC	34.239	0.119	4.03E-06	0.071	3.072	13.87	0.136	7.82E-06	0.137	6.210	17.21
BSU33210	yvrG	67.798	0.014	4.79E-07	0.004	0.185	6.94	0.025	1.44E-06	0.013	0.580	14.78
BSU33260	yvrN	44.343	0.028	9.59E-07	0.013	0.564	35.43	0.052	3.01E-06	0.041	1.844	15.29
BSU33270	yvrO	25.477	0.003	1.08E-07	0.003	0.110	32.08	0.013	7.20E-07	0.017	0.769	55.15
BSU33330	yvsH	50.258	0.004	1.21E-07	0.001	0.063	29.37					
BSU37530	ywhC	24.703	0.039	1.33E-06	0.032	1.407	12.55	0.068	3.91E-06	0.095	4.307	9.96
BSU37230	ywjA	64.562	0.050	1.70E-06	0.016	0.689	14.66	0.053	3.02E-06	0.028	1.273	10.77
BSU37040	ywkB	33.641	0.064	2.19E-06	0.039	1.697	37.26	0.121	6.94E-06	0.124	5.614	19.98
BSU36610	ywnC	13.799	0.070	2.38E-06	0.104	4.508	21.14	0.152	8.74E-06	0.381	17.235	25.58
BSU36570	ywnG	18.464	0.093	3.17E-06	0.103	4.479	30.03	0.164	9.42E-06	0.307	13.874	31.27
BSU36500	ywoB	17.305	0.142	4.84E-06	0.168	7.299	17.23	0.333	1.91E-05	0.664	29.998	12.28
BSU36480	ywoD	48.654	0.004	1.46E-07	0.002	0.078	31.08	0.004	2.29E-07	0.003	0.128	63.35
BSU36450	ywoG	43.08						0.001	5.83E-08	0.001	0.037	37.10
BSU36280	ywqA	106.03						0.013	7.19E-07	0.004	0.185	107.57
BSU38780	yxkI	63.382	0.007	2.22E-07	0.002	0.091	30.68	0.013	7.18E-07	0.007	0.308	16.18
BSU38690	yxlC	11.996	0.002	5.95E-08	0.003	0.129	45.84	0.004	2.40E-07	0.012	0.545	29.10
BSU38790	yxzE	6.8355	0.004	1.44E-07	0.013	0.551	36.76					
BSU40980	yyaB	16.894	0.005	1.81E-07	0.006	0.279	30.94	0.005	2.66E-07	0.009	0.429	12.01
BSU40520	yybS	34.534	0.014	4.65E-07	0.008	0.352	5.42	0.019	1.11E-06	0.019	0.877	24.99
BSU40490	yycA	73.592	0.003	1.14E-07	0.001	0.040	14.89	0.004	2.31E-07	0.002	0.085	47.99
BSU40480	yycB	43.184	0.008	2.80E-07	0.004	0.169	14.65	0.007	4.07E-07	0.006	0.256	25.98
BSU40150	yydI	23.882	0.003	9.14E-08	0.002	0.100	17.41	0.004	2.46E-07	0.006	0.280	9.91
BSU40140	yydJ	27.505	0.002	5.65E-08	0.001	0.054	40.74	0.003	1.55E-07	0.003	0.153	50.99

Accession number	Locus tag	Mol. Weight [kDa]	Control ng/µg	Control fg/µm²	Control molecules/µm²	Control molecules/cell	Control RSD (%)	1% subtilin ng/µg	1% subtilin fg/µm²	1% subtilin molecules/µm²	1% subtilin molecules/cell	1% subtilin RSD (%)
BSU02850	znuA	35.66	0.023	7.70E-07	0.013	0.564	24.60	0.007	3.74E-07	0.006	0.285	24.00
BSU02860	znuC	26.319	0.013	4.58E-07	0.010	0.455	30.10	0.034	1.92E-06	0.044	1.987	13.94

Supplementary Table S5. All membrane proteins quantified in this study via shotgun-MS and calibrated by targeted-MS. Protein concentration is specified as mass fraction (ng/µg crude membrane extract), as mass per total membrane area (fg/µm2), as membrane protein copy number per total area (molecules/µm2) and as molecules per cell (molecules/cell). Column “RSD (%)” lists the percental relative standard deviation of proteins obtained by biological replicates in shotgun MS. Proteins with a standard deviation higher than 31% (printed in red) were not considered as reliably quantified.

Supplementary Table S6. Membrane protein abundances significantly changed during production stress.

					Induced			Non Induced		
Accession number	Locus Tag	Regulation	LOG(P-value)	Fold-change	Average Normalized	molecules/μm^2	RSD (%)	Average Normalized	molecules/μm^2	RSD (%)
BSU28910	pftA	↑	2.71	56.1	-1.83	1.048	23.58	-3.57	0.009	64.43
BSU35690	ggaA	↑	3.66	52.2	-3.58	0.018	18.33	-5.29	0.000	45.64
BSU28900	pftB	↑	2.24	13.4	-2.63	0.164	29.02	-3.76	0.006	78.83
BSU05920	tsaB	↑	3.36	13.3	-4.29	0.004	12.21	-5.42	0.000	40.76
BSU33360	yvgJ	↑	4.17	12.8	-4.24	0.004	9.90	-5.34	0.000	24.30
BSU32110	yumC	↑	1.98	8.43	-4.27	0.004	14.23	-5.20	0.000	73.86
BSU07710	ltaS	↑	2.52	7.97	-3.23	0.041	11.48	-4.13	0.002	54.78
BSU35530	tagO	↑	2.86	7.02	-3.50	0.022	29.16	-4.35	0.001	31.28
BSU36250	ptkA	↑	3.42	5.87	-3.78	0.011	13.44	-4.55	0.001	21.31
BSU05500	mhqP	↑	1.85	5.48	-3.54	0.020	26.12	-4.28	0.002	59.05
BSU18830	pps	↑	3.38	5.34	-4.68	0.001	19.26	-5.41	0.000	15.34
BSU24950	pstBB	↑	2.84	4.89	-2.89	0.088	7.49	-3.58	0.007	33.93
BSU18450	gltA	↑	2.98	4.73	-3.90	0.009	9.71	-4.58	0.001	27.14
BSU03540	ycxB	↑	4.14	4.64	-3.77	0.012	13.97	-4.43	0.001	5.23
BSU07260	yfnI	↑	4.81	4.23	-3.06	0.059	8.36	-3.69	0.005	7.56
BSU24970	pstA	↑	2.73	4.16	-2.94	0.079	20.28	-3.56	0.007	26.20
BSU22840	yphC	↑	3.06	3.82	-3.59	0.018	20.55	-4.17	0.002	13.30
BSU27100	yrhP	↑	3.26	3.80	-3.46	0.024	9.63	-4.04	0.002	23.32
BSU14770	ylaG	↑	2.87	3.79	-2.19	0.445	5.75	-2.77	0.047	25.38
BSU32040	yuiF	↑	1.79	3.77	-3.63	0.017	35.64	-4.21	0.002	36.81
BSU14880	ctaB	↑	2.87	3.71	-2.83	0.102	8.52	-3.40	0.011	28.57
BSU24960	pstBA	↑	4.13	3.71	-2.92	0.084	8.60	-3.48	0.009	8.43
BSU24980	pstC	↑	2.81	3.69	-3.14	0.050	8.74	-3.70	0.005	28.57
BSU16930	cinA	↑	2.81	3.46	-3.68	0.014	20.98	-4.22	0.002	19.15
BSU11720	fabI	↑	5.27	3.30	-3.13	0.051	4.67	-3.65	0.006	4.63
BSU01580	ybaR	↑	2.02	3.25	-3.91	0.009	27.23	-4.42	0.001	29.57
BSU02980	opuAA	↑	1.90	3.23	-3.33	0.033	20.24	-3.83	0.004	43.97

Accession number	Locus Tag	Regulation	LOG(P-value)	Fold-change	Induced			Non Induced		
					Average Normalized	molecules/μm²	RSD (%)	Average Normalized	molecules/μm²	RSD (%)
BSU04340	ydaP	↑	2.96	2.84	-5.04	0.001	10.18	-5.50	0.000	17.88
BSU25570	comEC	↑	1.12	2.79	-4.53	0.002	55.84	-4.97	0.000	42.77
BSU01590	ybaS	↑	1.32	2.77	-3.97	0.008	24.24	-4.41	0.001	62.58
BSU05470	ydfM	↑	2.99	2.61	-3.87	0.009	6.44	-4.28	0.001	17.24
BSU02710	yczC	↑	2.41	2.58	-3.84	0.010	10.65	-4.25	0.002	24.21
BSU05410	ydfH	↑	1.46	2.54	-4.54	0.002	26.43	-4.94	0.000	45.79
BSU04310	ydaN	↑	1.48	2.54	-4.68	0.002	37.63	-5.08	0.000	31.57
BSU24680	comGF	↑	1.41	2.52	-3.23	0.042	35.53	-3.63	0.007	41.93
BSU24690	comGE	↑	1.79	2.52	-3.27	0.039	36.41	-3.67	0.006	13.29
BSU00290	darA	↑	1.27	2.45	-3.86	0.010	27.66	-4.25	0.002	45.05
BSU19390	yojN	↑	3.39	2.43	-3.68	0.014	10.19	-4.07	0.002	8.54
BSU33270	yvrO	↑	1.08	2.40	-3.65	0.017	55.15	-4.03	0.003	32.08
BSU07320	yfnC	↑	1.06	2.36	-3.56	0.019	23.83	-3.93	0.004	67.00
BSU29805	sftA	↑	3.93	2.34	-3.76	0.012	6.51	-4.13	0.002	5.91
BSU12000	manR	↑	2.06	2.32	-3.19	0.046	22.57	-3.55	0.008	18.66
BSU23100	sigX	↑	2.60	2.28	-2.68	0.143	4.39	-3.04	0.025	20.73
BSU40100	ahpF	↑	1.41	2.26	-3.48	0.023	25.59	-3.83	0.004	38.23
BSU31580	maeN	↑	2.08	2.21	-4.09	0.006	25.90	-4.43	0.001	4.36
BSU29730	motP	↑	0.99	2.20	-3.76	0.013	36.37	-4.10	0.002	41.28
BSU02230	purT	↑	2.73	2.20	-4.18	0.005	20.47	-4.52	0.001	2.99
BSU11640	yjbQ	↑	2.07	2.19	-3.72	0.013	25.20	-4.06	0.002	10.61
BSU33000	htrB	↑	1.07	2.12	-3.86	0.010	46.74	-4.19	0.002	9.01
BSU03440	tlpC	↑	1.17	2.10	-4.94	0.001	41.22	-5.26	0.000	39.18
BSU02560	ycbM	↑	2.42	2.08	-5.04	0.001	13.51	-5.36	0.000	14.32
BSU23090	rsiX	↑	2.57	2.08	-2.77	0.119	11.00	-3.08	0.022	15.45
BSU16950	pbpX	↑	2.63	2.05	-2.69	0.143	15.90	-3.00	0.027	4.52
BSU12290	ndh	↑	2.31	2.02	-2.70	0.138	18.53	-3.01	0.026	12.38
BSU02570	ycbN	↑	2.64	2.00	-4.21	0.004	5.97	-4.51	0.001	14.76
BSU29680	acsA	↑	2.36	1.99	-3.61	0.017	17.15	-3.91	0.003	6.73

					Induced			Non Induced		
Accession number	Locus Tag	Regulation	LOG(P-value)	Fold-change	Average Normalized	molecules/μm^2	RSD (%)	Average Normalized	molecules/μm^2	RSD (%)
BSU36260	tkmA	↑	2.43	1.98	-3.14	0.050	13.35	-3.44	0.010	12.86
BSU24670	comGG	↑	1.06	1.97	-2.99	0.077	53.04	-3.28	0.014	4.13
BSU04490	ydbJ	↑	1.81	1.93	-2.73	0.130	4.90	-3.01	0.027	27.91
BSU04610	ydcA	↑	1.66	1.93	-4.19	0.005	28.14	-4.47	0.001	5.97
BSU14210	ykuT	↑	2.74	1.93	-3.59	0.018	8.93	-3.88	0.004	12.91
BSU35560	tuaF	↑	1.99	1.89	-3.89	0.009	6.73	-4.17	0.002	22.02
BSU38510	dltB	↑	2.48	1.89	-3.33	0.032	10.09	-3.60	0.007	16.64
BSU11340	fabF	↑	1.33	1.88	-4.18	0.005	21.41	-4.46	0.001	29.16
BSU09070	yhcG	↑	1.55	1.85	-3.68	0.015	17.46	-3.95	0.003	24.96
BSU31110	yubF	↑	1.50	1.84	-2.17	0.478	21.36	-2.43	0.101	24.47
BSU22380	tseB	↑	2.56	1.83	-3.04	0.063	13.61	-3.31	0.013	10.64
BSU35750	tagA	↑	2.14	1.81	-3.30	0.035	18.15	-3.55	0.007	12.38
BSU01490	rplM	↑	2.47	1.79	-2.89	0.090	7.66	-3.14	0.020	13.87
BSU07460	yfmI	↑	1.55	1.75	-4.27	0.004	26.87	-4.51	0.001	9.88
BSU25290	era	↑	1.66	1.71	-3.18	0.046	22.03	-3.41	0.010	14.10
BSU31490	pbpD	↑	2.01	1.69	-2.70	0.137	10.64	-2.93	0.032	16.98
BSU38280	efeU	↑	1.77	1.69	-3.34	0.032	21.73	-3.56	0.007	2.23
BSU02750	natA	↑	1.34	1.66	-3.81	0.011	20.64	-4.03	0.003	19.00
BSU13430	ykoX	↑	2.32	1.65	-3.50	0.022	5.67	-3.72	0.005	12.99
BSU19340	yocR	↑	1.42	1.63	-3.73	0.013	19.39	-3.95	0.003	15.26
BSU14590	pdhB	↑	1.64	1.58	-2.34	0.319	12.63	-2.54	0.079	17.66
BSU22320	ponA	↑	2.27	1.57	-2.86	0.095	11.59	-3.06	0.023	10.36
BSU07400	yfmO	↑	1.74	1.54	-3.35	0.031	15.34	-3.54	0.008	9.96
BSU02290	psd	↑	1.73	1.54	-3.41	0.027	11.04	-3.60	0.007	16.61
BSU03600	tcyB	↑	2.24	1.50	-2.62	0.165	8.09	-2.80	0.043	12.08
BSU07800	treP	↑	2.27	1.47	-2.06	0.596	12.56	-2.23	0.156	5.10
BSU13890	ptsG	↑	2.74	1.46	-1.81	1.079	9.63	-1.97	0.284	2.18
BSU23110	resE	↓	2.31	0.68	-2.86	0.096	5.85	-2.69	0.054	10.27
BSU29780	ytxG	↓	1.61	0.61	-2.33	0.325	13.68	-2.12	0.207	20.10

					Induced			Non Induced		
Accession number	Locus Tag	Regulation	LOG(P-value)	Fold-change	Average Normalized	molecules/μm²	RSD (%)	Average Normalized	molecules/μm²	RSD (%)
BSU23970	artQ	↓	1.40	0.61	-2.29	0.361	21.52	-2.07	0.230	21.01
BSU38770	cimH	↓	1.27	0.59	-3.11	0.055	23.66	-2.88	0.036	22.64
BSU25390	yqeZ	↓	1.35	0.59	-3.13	0.051	6.72	-2.90	0.034	32.00
BSU40980	yyaB	↓	1.44	0.58	-3.86	0.009	12.01	-3.63	0.006	30.94
BSU11450	oppC	↓	1.90	0.58	-2.34	0.321	18.95	-2.10	0.214	13.03
BSU23960	artR	↓	1.28	0.57	-2.51	0.218	22.14	-2.27	0.148	26.11
BSU11440	oppB	↓	2.23	0.57	-2.25	0.395	19.57	-2.00	0.264	0.41
BSU25190	cccA	↓	1.29	0.56	-2.75	0.125	19.23	-2.50	0.088	30.45
BSU13300	mgtE	↓	2.32	0.56	-3.09	0.057	13.32	-2.84	0.039	12.83
BSU23140	resB	↓	2.90	0.56	-3.03	0.065	8.94	-2.77	0.045	9.49
BSU23130	resC	↓	1.83	0.56	-2.88	0.092	12.92	-2.62	0.064	19.88
BSU40480	yycB	↓	1.46	0.55	-4.10	0.006	25.98	-3.84	0.004	14.65
BSU23150	resA	↓	3.42	0.55	-2.73	0.128	1.95	-2.47	0.090	9.17
BSU10220	gltT	↓	3.19	0.54	-2.05	0.617	10.13	-1.78	0.440	2.86
BSU22550	qcrB	↓	2.23	0.53	-2.91	0.085	18.07	-2.64	0.061	4.79
BSU02950	niaP	↓	1.36	0.53	-4.24	0.004	36.77	-3.97	0.003	6.56
BSU36520	nrgB	↓	1.04	0.52	-4.36	0.003	41.78	-4.07	0.002	32.81
BSU01630	feuA	↓	2.90	0.52	-3.28	0.036	10.49	-3.00	0.027	10.22
BSU22540	qcrC	↓	2.72	0.51	-3.25	0.039	14.07	-2.95	0.030	10.44
BSU31010	floT	↓	1.96	0.50	-2.41	0.266	11.01	-2.11	0.210	24.99
BSU13000	ykfD	↓	1.72	0.48	-4.74	0.001	29.18	-4.42	0.001	10.38
BSU07610	citM	↓	3.71	0.46	-2.44	0.250	10.56	-2.11	0.208	4.16
BSU33320	fhuD	↓	2.75	0.46	-2.71	0.135	17.64	-2.38	0.112	7.43
BSU13990	kinA	↓	0.93	0.44	-5.26	0.000	64.66	-4.91	0.000	26.31
BSU03020	ycgA	↓	1.73	0.44	-3.89	0.009	21.68	-3.54	0.008	26.41
BSU21750	sco	↓	2.77	0.43	-3.26	0.038	15.56	-2.90	0.034	10.31
BSU33500	copA	↓	1.64	0.42	-3.54	0.020	4.24	-3.17	0.019	41.98
BSU03220	putP	↓	2.79	0.42	-2.55	0.195	17.43	-2.18	0.177	11.11
BSU04060	ycsG	↓	3.65	0.42	-3.00	0.069	12.74	-2.63	0.063	2.87

					Induced			Non Induced		
Accession number	Locus Tag	Regulation	LOG(P-value)	Fold-change	Average Normalized	molecules/μm²	RSD (%)	Average Normalized	molecules/μm²	RSD (%)
BSU02340	gltP	↓	1.52	0.42	-3.94	0.008	**38.07**	-3.57	0.007	21.90
BSU13490	htpX	↓	2.24	0.42	-2.76	0.121	3.60	-2.38	0.114	26.20
BSU11900	yjcL	↓	2.64	0.39	-4.32	0.003	15.46	-3.91	0.003	18.86
BSU04450	dctS	↓	1.33	0.39	-5.13	0.001	**53.93**	-4.73	0.001	12.12
BSU04360	mntH	↓	4.09	0.39	-3.51	0.021	8.07	-3.10	0.021	4.32
BSU35280	yvjA	↓	2.15	0.38	-3.86	0.010	25.72	-3.44	0.010	21.94
BSU31640	mrpE	↓	0.89	0.36	-3.96	0.010	**97.03**	-3.52	0.008	12.35
BSU02440	glnK	↓	1.98	0.36	-4.44	0.003	**34.93**	-3.99	0.003	10.23
BSU01680	murP	↓	2.02	0.35	-4.80	0.001	30.84	-4.35	0.001	22.45
BSU22560	qcrA	↓	2.97	0.32	-3.01	0.067	4.52	-2.52	0.083	20.32
BSU11400	appC	↓	1.30	0.31	-4.15	0.005	**49.75**	-3.64	0.006	29.98
BSU30270	msmE	↓	4.29	0.28	-4.16	0.005	4.56	-3.60	0.007	10.16
BSU09720	bmrD	↓	3.92	0.24	-3.86	0.010	13.89	-3.24	0.015	11.46
BSU11360	appD	↓	1.88	0.23	-4.95	0.001	**47.17**	-4.32	0.001	8.31
BSU14910	ctaE	↓	3.07	0.23	-3.17	0.047	7.42	-2.52	0.082	27.64
BSU33810	opuCC	↓	2.71	0.22	-4.76	0.001	29.50	-4.10	0.002	18.13
BSU09710	bmrC	↓	1.98	0.21	-3.68	0.014	1.41	-3.01	0.030	**63.38**
BSU25370	yqfB	↓	2.37	0.20	-3.97	0.008	**52.61**	-3.28	0.014	5.51
BSU14930	ctaG	↓	3.04	0.20	-4.27	0.004	9.67	-3.57	0.007	29.88
BSU38730	cydD	↓	3.18	0.20	-4.18	0.005	15.07	-3.47	0.009	24.00
BSU02850	znuA	↓	3.01	0.19	-4.05	0.006	24.00	-3.32	0.013	24.60
BSU03060	lctP	↓	3.59	0.17	-4.08	0.006	13.25	-3.32	0.013	21.80
BSU14890	ctaC	↓	4.07	0.17	-3.15	0.049	19.28	-2.39	0.108	2.64
BSU14900	ctaD	↓	3.52	0.17	-3.43	0.026	11.15	-2.66	0.060	25.01
BSU38750	cydB	↓	3.52	0.15	-3.68	0.015	6.43	-2.85	0.038	25.89
BSU14920	ctaF	↓	3.24	0.14	-3.57	0.019	2.02	-2.72	0.052	**33.73**
BSU38760	cydA	↓	3.28	0.14	-3.91	0.009	25.42	-3.05	0.024	16.48
BSU30770	mntA	↓	4.22	0.13	-3.18	0.046	17.01	-2.31	0.132	12.28
BSU11370	appF	↓	2.19	0.13	-4.45	0.003	**49.66**	-3.57	0.007	19.94

					Induced			Non Induced		
Accession number	Locus Tag	Regulation	LOG(P-value)	Fold-change	Average Normalized	molecules/μm²	RSD (%)	Average Normalized	molecules/μm²	RSD (%)
BSU30760	mntB	↓	3.24	0.13	-3.70	0.014	10.23	-2.80	0.044	32.72
BSU33830	opuCA	↓	2.36	0.12	-5.10	0.001	26.32	-4.18	0.002	56.16
BSU30750	mntC	↓	4.93	0.11	-4.21	0.004	10.88	-3.26	0.015	5.99
BSU38740	cydC	↓	3.81	0.11	-4.30	0.004	29.44	-3.34	0.012	5.80
BSU11390	appB	↓	3.68	0.05	-4.76	0.001	11.56	-3.48	0.009	34.85
BSU37180	fadF	↓	4.72	0.02	-4.68	0.001	25.40	-3.05	0.024	13.33
BSU02270	pssA	ON				0.016	29.67			
BSU03250	ycgR	ON				0.001	36.02			
BSU07450	yfmJ	ON				0.004	32.01			
BSU08460	yfhA	OFF							0.001	30.28
BSU09970	yhaJ	ON				0.002	20.59			
BSU14440	ykpB	ON				0.002	27.85			
BSU15340	ylmA	ON				0.001	27.38			
BSU19180	des	ON				0.001	36.32			
BSU31460	kapB	ON				0.001	37.47			
BSU31550	nupO	OFF							0.000	29.13
BSU31570	nupQ	OFF							0.000	19.25
BSU33120	liaH	ON				0.017	67.49			
BSU33330	yvsH	OFF							0.001	29.37
BSU36280	ywqA	ON				0.004	107.57			
BSU36450	ywoG	ON				0.001	37.10			
BSU37250	narI	OFF							0.005	6.28
BSU37270	narH	OFF							0.001	81.91
BSU37280	narG	OFF							0.002	13.64
BSU37320	narK	OFF							0.011	31.24
BSU38790	yxzE	OFF							0.013	36.76
BSU39390	hutM	OFF							0.003	18.77
BSU02170	ybfB	OFF							0.000	23.69

Supplementary Table S6. Membrane protein abundances significantly changed during production stress. Higher **(↑)** or lower **(↓)** abundance of a specific protein in comparison to control (no induction) is depicted with an arrow. Lower part of the table depicts the proteins that were "ON" or "OFF" in comparison to control. Significance was calculated using a permutation-based FDR approach implemented in Perseus [5]. Column fold-change was calculated by $10^{(\text{average normalized induced} - \text{average normalized non-induced})}$. Column "average normalized" lists the average of molecules/μm^2 normalized by the total sum on column and presented on $\log_{10}$ scale. Column molecules/μm^2 is presented as average for each condition tested. Column "RSD (%)" lists the percental standard deviation of proteins obtained by biological replicates in shotgun MS. Proteins with a standard deviation higher than 31% (printed in red) were not considered as reliably quantified.

Supplementary Table S7. Stoichiometry information for selected proteins

	B. subtilis	Literature	Organism
SecDF:SecG:SecY:SpoIIIJ:YrbF	**1.0** ± 0.1 : **0.3** ± 0.0 : **0.7** ± 0.1 : **1.1** ± 0.3 : **4.3** ± 0.5	1 : 1 : 1 : 1 : 1: 1	*E. coli* [6]

Supplementary Table S7. Stoichiometry composition of known protein complexes was determined using the absolute membrane protein quantification recently published [7] and compared to previous observations (column “literature”). The standard deviation between replicates is also presented in the table. Literature values were extracted from the indicated references as well as the organism in which these studies were performed.

REFERENCES

(1) Mäder, U.; Schmeisky, A. G.; Flórez, L. A.; Stülke, J. SubtiWiki - A comprehensive community resource for the model organism *Bacillus subtilis*. *Nucleic Acids Res.* **2012**, *40* (D1), 1278–1287.

(2) Reuß, D. R.; Altenbuchner, J.; Mäder, U.; Rath, H.; Ischebeck, T.; Sappa, P. K.; Thürmer, A.; Guérin, C.; Nicolas, P.; Steil, L.; *et al*. Large-scale reduction of the *Bacillus subtilis* genome: consequences for the transcriptional network, resource allocation, and metabolism. *Genome Res.* **2017**, *27* (2), 289–299.

(3) Bongers, R. S.; Veening, J.-W.; Van Wieringen, M.; Kuipers, O. P.; Kleerebezem, M. Development and characterization of a subtilin-regulated expression system in *Bacillus subtilis*: strict control of gene expression by addition of subtilin. *Appl. Environ. Microbiol.* **2005**, *71* (12), 8818–8824.

(4) Suárez, R. A.; Stülke, J.; Van Dijl, J. M. Less is more: toward a genome-reduced *Bacillus* cell factory for “difficult proteins.” *ACS Synth. Biol.* **2019**, *8* (1), 99–108.

(5) Tyanova, S.; Temu, T.; Sinitcyn, P.; Carlson, A.; Hein, M. Y.; Geiger, T.; Mann, M.; Cox, J. The Perseus computational platform for comprehensive analysis of (Prote)Omics data. *Nat. Methods* **2016**, *13* (9), 731–740.

(6) Papanastasiou, M.; Aivaliotis, M.; Karamanou, S.; Koukaki, M.; Sardis, M. F.; Orfanoudaki, G.; Kountourakis, N.; Economou, A. The *Escherichia coli* peripheral inner membrane proteome . *Mol. Cell. Proteomics* **2012**, *12* (3), 599–610.

(7) Antelo-Varela, M.; Bartel, J.; Quesada-Ganuza, A.; Appel, K.; Bernal-Cabas, M.; Sura, T.; Otto, A.; Rasmussen, M.; van Dijl, J. M.; Nielsen, A.; *et al*. Ariadne’s thread in the analytical labyrinth of membrane proteins: integration of targeted and shotgun proteomics for global absolute quantification of membrane proteins. *Anal. Chem.* **2019**, *91* (18), 11972–11980.
